# Effect of the Aza-N-Bridge and Push–Pull Moieties: A Comparative Study between BODIPYs and Aza-BODIPYs

**DOI:** 10.1021/acs.joc.1c02525

**Published:** 2022-02-21

**Authors:** Clara Schäfer, Jürgen Mony, Thomas Olsson, Karl Börjesson

**Affiliations:** Department of Chemistry and Molecular Biology, University of Gothenburg, Kemivägen 10, 41296 Gothenburg, Sweden

## Abstract

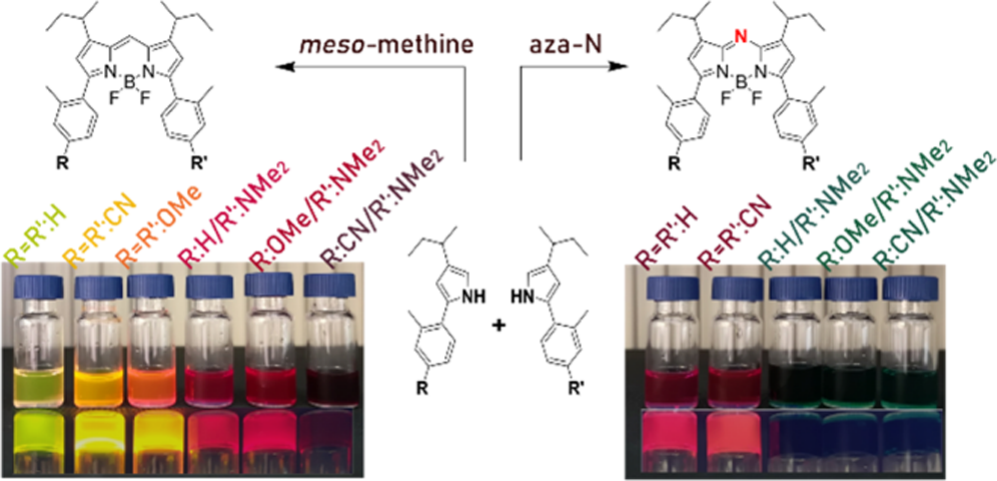

In the field of fluorescent dyes, difluoroboron-dipyrromethenes (BODIPY) have a highly respected position. To predict their photophysical properties prior to synthesis and therefore to successfully design molecules specifically for one’s needs, a solid structure–function understanding based on experimental observations is vital. This work delivers a photophysical evaluation of BODIPY and aza-BODIPY derivatives equipped with different electron-withdrawing/-donating substituents. Using combinatorial chemistry, pyrroles substituted with electron-donating/-withdrawing substituents were condensed together in two different manners, thus providing two sets of molecules. The only difference between the two sets is the bridging unit providing a so far lacking comparison between BODIPYs and aza-BODIPYs structural homologues. Replacing the *meso*-methine bridge with an aza-N bridge results in a red-shifted transition and considerably different, temperature-activated, excited-state relaxation pathways. The effect of electron-donating units on the absorption but not emission for BODIPYs was suppressed compared to aza-BODIPYs. This result could be evident in a substitution pattern-dependent Stokes shift. The outlook of this study is a deeper understanding of the structure–optics relationship of the (aza)-BODIPY-dye class, leading to an improvement in the *de novo* design of tailor-made molecules for future applications.

## Introduction

A detailed understanding of structure–function relationship is the foundation for successfully tuning molecular properties to fit specific applications. For several biological assays or screening experiments as well as in fluorescence microscopy, just to name an example, dyes with near-infrared absorption and emission are preferable due to less interference from autofluorescence and a larger penetration depth.^1,2^ BODIPY dyes and their derivatives have become increasingly popular due to their competitive optical properties like large molar absorption coefficients, sharp fluorescent bands, high fluorescent quantum yields, high photostability, and good biocompatibility.^3^ They are used in fluorescent sensors,^4^ as fluorescent probes^5−7^ in, for instance, microscopy,^1^ and also in the field of optoelectronics^8^ such as donor–acceptor conjugates in solar cells^9^ just to name a few applications for this versatile dye class. They also are easily synthetically modified, which leads to easily tunable photophysical properties.^10−12^

To red-shift the absorption and emission of BODIPYs, the most atom-effective method is to replace the *meso*-methine bridge, which connects the two pyrrole components, with an aza-N bridge. The resulting dye class is known as aza-BODIPYs and was discovered in 1943.^13^ Only in the last couple of years, aza-BODIPYs have attracted increasing interest due to their versatility.^14,15^ This aforementioned small change in the structure can lead to a redshift of about 80 nm, leading to some derivatives absorbing in the red or near-infrared regions of the electromagnetic spectrum.^16−19^ However, a general observation is that aza-BODIPY have lower emission quantum yields compared to BODIPY dyes,^15,19^ making them less desirable in applications where photons are used as a readout signal. In contrast, they show a higher potential to undergo singlet-to-triplet intersystem crossing,^20^ which in combination with their red-shifted absorption leads to new utilizations like photodynamic therapy.^21^

To push the absorption and emission wavelength even further into the near-infrared region of the electromagnetic spectrum, push–pull systems can be used.^17,22−24^ By adding electron-donating groups (push moiety) in the *para-*position of the 3,5-phenyls of the (aza)-BODIPY, the HOMO-energy level is increased, whereas electron-withdrawing groups (pull moiety) in *para-*position of the 1,7 phenyls of the (aza)-BODIPY decreases the LUMO-energy level.^17,24,25^ The decreased energy gap leads to a redshift of absorption and emission.

**Figure 1:**
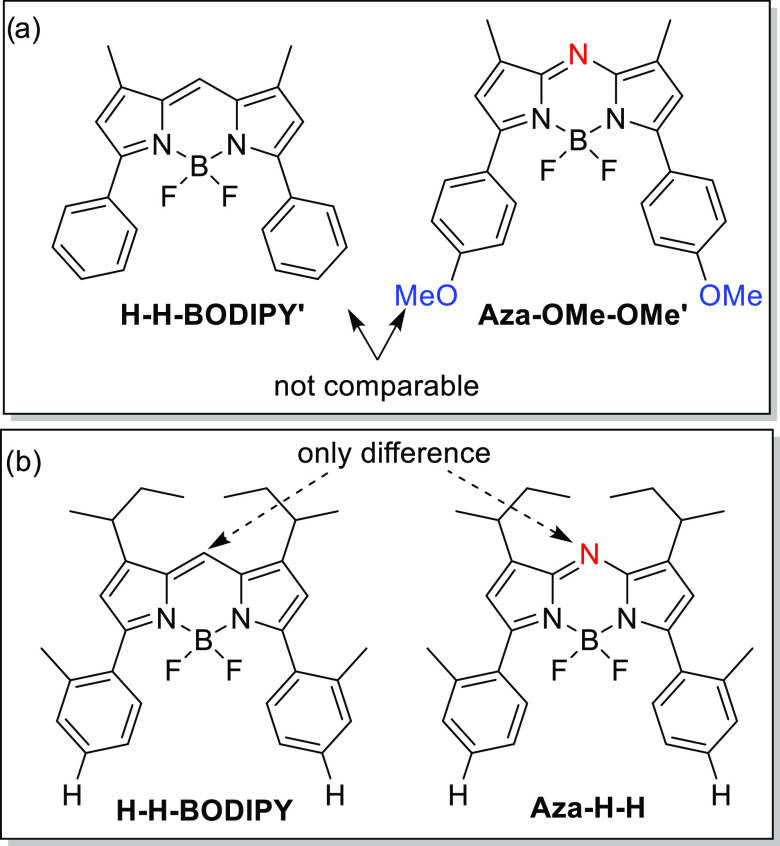
Structure of previous comparative study between aza-BODIPY and BODIPY using two noncomparable molecules (a) and this study using comparable structural homologues (b).

The photophysical features of BODIPY^5,23,26,27^ and aza-BODIPY^28−33^ dye classes have been extensively studied individually; conversely, there is almost no simultaneous comparison of BODIPY and aza-BODIPY structural analogues.^18,19^ Since aza-BODIPYs are a subclass to BODIPY dyes, it seems intriguing that these classes have, to our knowledge, not yet been accurately compared. The origin of the redshift in absorption and emission maxima observed for the aza-BODIPYs compared to the BODIPYs has been investigated; however, the authors used structurally incomparable homologues (Figure 1).^18^ Due to an added push effect to the aza-BODIPY, the reported effect of the aza-N bridge is most likely exaggerated. To accurately study how structural modifications affect the photophysical properties of BODIPYs and aza-BODIPYs, one must study molecules wherein the substitution to the dipyrromethene is the same and the only structural deviation is the aza-N bridge.

Here, we designed, synthesized, and studied structural homologues of BODIPYs and aza-BODIPYs to give detailed information on how the substitution pattern of electron-donating and withdrawing substituents influences the photophysical properties and how these classes differ from another. We start by describing the combinatorial synthesis approach used, which allowed access to two sets of asymmetric push and pull systems with the only difference in-between being the bridging atom, C for BODIPY and N for aza-BODIPY, which allowed us to accurately assess the effect of the substitution. The expected redshift in the absorption was not the only major difference between the two sets found. Although aza-BODIPYs showed a textbook mirror image relationship between the absorption and emission envelopes, the absorption but not emission of BODIPYs was significantly broadened. Further, aza-BODIPYs contain a temperature-activated nonradiative relaxation pathway that did not exist in the BODIPYs. In summary, replacing the *meso*-methine bridge with an aza-N bridge does not only result in a red-shifted transition, but also the whole ground-state structure and excited-state relaxation pathways are significantly perturbed.

## Results and Discussion

To allow for a direct comparison between BODIPY and aza-BODIPY derivatives, both in their unsubstituted forms and when incorporating electron-donating/-accepting substituents, two sets of molecules were designed. The first set was BODIPYs having electron-accepting and/or -donating units in the para-position of the 3,5-phenyls of the BODIPY core. The second was the aza-BODIPY structural equivalents. Importantly, to allow for a direct comparison of the effect of the aza-N-bridge, the meso-methine bridge was left unsubstituted. For better solubility, the molecules were equipped with sec-butyl substituents in positions 1 and 7. Furthermore, the effect of the extended aromatic system in the α position (3 and 5) was evaluated by synthesizing the alkylated BODIPY derivative.

The various differently substituted BODIPY as well as aza-BODIPY derivatives were synthesized from pyrroles. Several α-substituted pyrroles were synthesized (see Scheme S2) using a Suzuki–Miyaura coupling reaction between an alkylated pyrrole boronic acid with arylbromides, carrying a methyl group in ortho position and different electron-withdrawing or -donating substituents in the para position. Since pyrrole boronic acids are known to be unstable under elevated temperatures and basic conditions, the choice of catalyst plays an important role in this reaction.^34−36^ To successfully couple the arylbromides to the pyrrole boronic acid, a palladium precatalyst with a fast generation of the active Pd(0) species under mild conditions^37^ had to be used to avoid deboronation.^38^

**Scheme 1:**
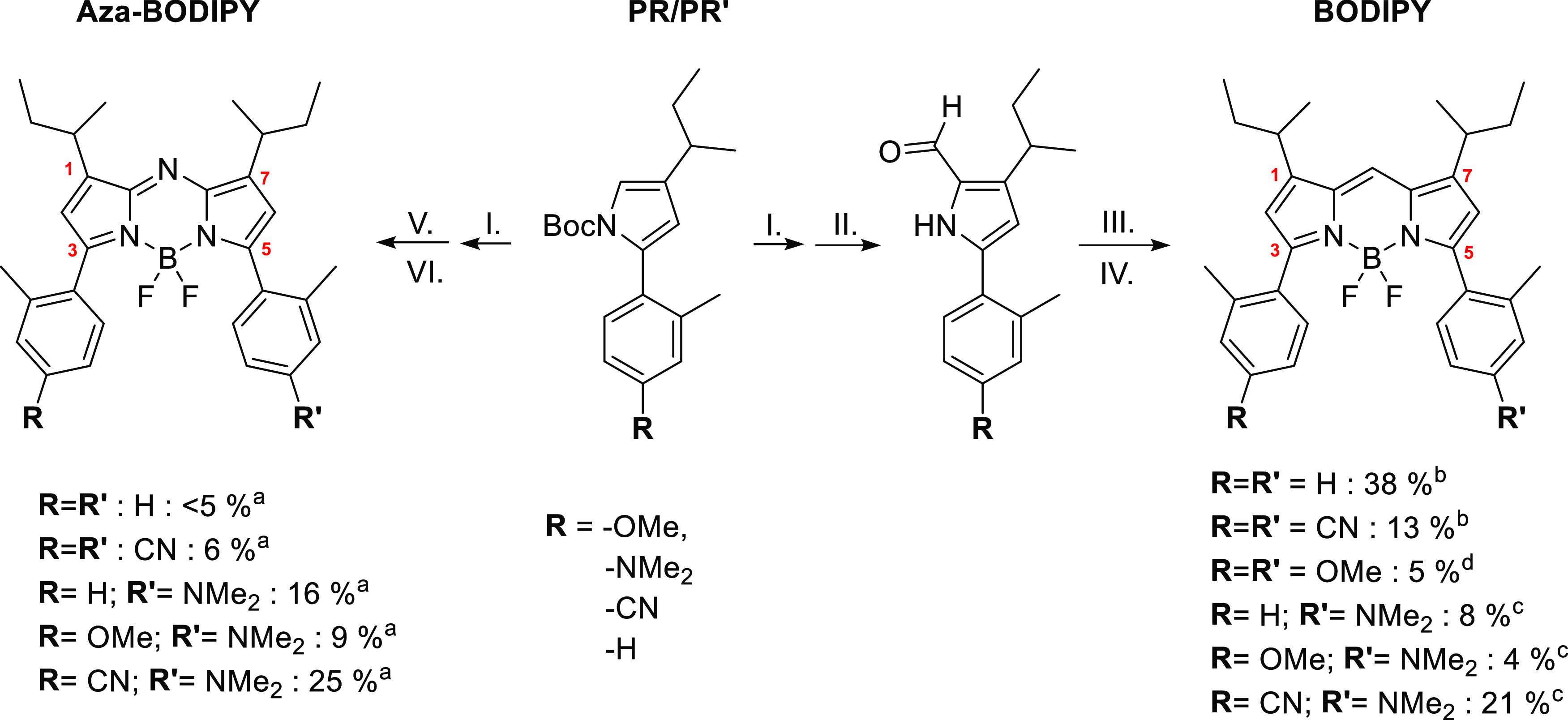
Synthesis Routes from Boc-Protected Pyrroles (PR/PR′) to aza-BODIPYs (Left Route) and BODIPYs (Right Route) (I) NaOMe (25 wt % in MeOH), THF (II) 1. POCl_3_, DMF, DCE 2. NaOAc, H_2_O; (III) **R = R′**: POCl_3_, DCM/**R≠R′**: PR′, AcOH, DCM; (IV) Et_3_N, BF_3_·OEt_2_, DCM; (V) **R = R′**: NaNO_2_, AcOH, Ac_2_O/**R≠R′**: 1. NaNO_2_, AcOH 2. PR′, Ac_2_O; (VI) Et_3_N, BF_3_·OEt_2_, DCM. Yields were obtained over three steps starting with the Boc-protected pyrrole (1. Boc-deprotection, 2. condensation to the aza-dipyrromethene, and 3. chelation to the aza-BODIPY). Yields were obtained over two steps starting with the α-formylated pyrrole (1. condensation to the dipyrromethene and 2. chelation to the BODIPY). Yields were obtained over two steps starting with the Boc-protected pyrrole and the α-formylated pyrrole (1. condensation to the dipyrromethene and 2. chelation to the BODIPY). Compound was obtained as a side product during the synthesis of OMe-NMe_2_-BODIPY.

These pyrroles, after Boc-deprotection, would be subsequently used for condensation to symmetric and asymmetric aza-dipyrromethenes.^39^ These were in the last step chelated using BF_3_·OEt_2_ to obtain the aza-BODIPYs (see Scheme 1, left path). The same pyrroles, after first Boc-deprotection followed by formylation using the Vilsmeier–Haack reaction, would also be used for condensation to symmetric and asymmetric dipyrromethenes, which after chelation with BF_3_·OEt_2_ form the BODIPY derivatives (see Scheme 1, right path).^40^

This synthesis strategy allowed us to create a small library of BODIPYs and aza-BODIPYs from four different pyrroles. The derivatives being the same in regard to the substitution pattern and only different in the connection of the pyrrole units allows for the study of the effect of several substitution patterns on BODIPYs and aza-BODIPYs. Furthermore, to compare these effects, we take the change from the meso-methine bridge to the aza-N bridge into account.

**Figure 2:**
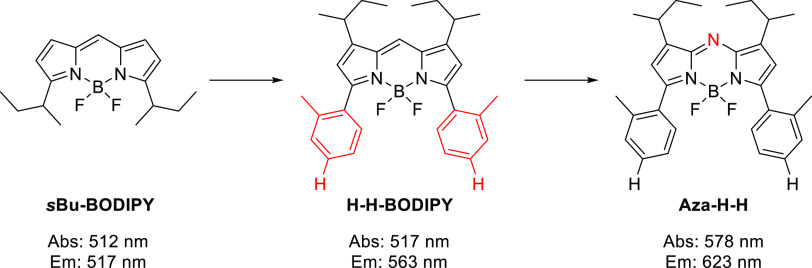
Extension of the aromatic system (sBu-BODIPY to H-H-BODIPY) and going from BODIPY to aza-BODIPY (H-H-BODIPY to Aza-H-H).

To our knowledge, there is no reported study that shows if the redshift of the aza-BODIPY compared to BODIPY is solely from the aza-N bridge or to some extent from an extended aromatic system, which is common in aza-BODIPYs. To assess this, we will start by verifying the influence of the extended aromatic system by comparing an alkylated BODIPY to a BODIPY carrying aromatic substituents in positions 3 and 5 (*s*Bu-BODIPY → H-H-BODIPY; Figure 2). To evaluate the effect on the redshift by the aza-N-bridge, we continue by comparing this extended BODIPY with its aza-BODIPY homologue (H-H-BODIPY → aza-H-H). After verifying the origin of the redshift, we will finally investigate the substitution effects on the photophysical properties of the two sets, as well as compare the differences in the substitution patterns in regard to the bridging unit.

**Figure 3:**
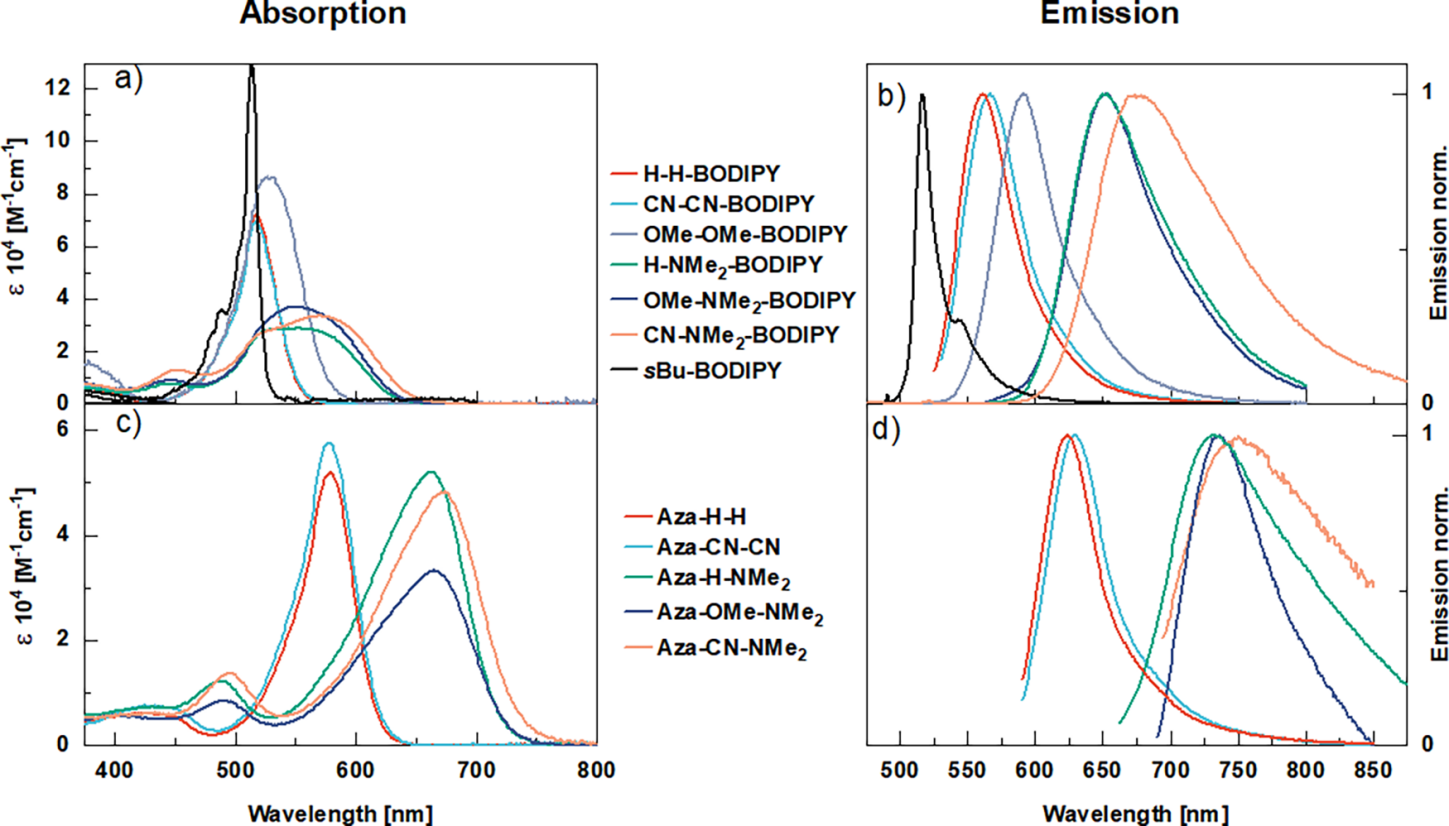
Absorption of BODIPY (a) and aza-BODIPY (c) and emission of BODIPY (b) and aza-BODIPY (d), all dissolved in toluene.

By comparing the absorption and emission maxima of sBu-BODIPY and H-H-BODIPY (Figures 2 and 3a,b), the effect of the extension of the aromatic system can be examined. Almost no change in the absorption maximum, 512–517 nm, was observed, although a broadening of the absorption envelope and a large increase in the Stokes shift could be seen (see Table 1 entries 1 and 2). Changing the *meso*-methine bridge of the H-H-BODIPY to an aza-N bridge results in the aza-BODIPY analogue (Aza-H-H). The absorption is now considerable red-shifted 578 nm (See Table 1 entries 2 and 8, and Figure 3a,c, red line), thus confirming the findings of a prior study on the origin of the redshift of aza-BODIPYs, in which a BODIPY was compared to a structurally similar, yet not the same aza-BODIPY.^18^ Interestingly, the two homologues, H-H-BODIPY and Aza-H-H, display a similar Stokes shift. In summary, structural homologues confirm prior assumptions that the aza-N bridge is solely responsible for the redshift of the absorption in aza-BODIPYs, and the origin of the relatively large Stokes shift seen in aza-BODIPYs is probable due to the presence of the α-phenyl substituents that are so far necessary when synthesizing aza-BODIPYs.^15,41^

**Table 1:** Photophysical Characterization of Synthesized BODIPY Derivatives (Entries 1–7) and Aza-BODIPY Derivatives (Entries 8–11)^g^

	λ_max abs_ [nm]	λ_max em_ [nm]	Stokes shift (cm^–1^)	Φ_f_	τ [ns]	*k*_f_ × 10^8^ [s^–1^]^a^	*k*_NR_ × 10^8^ [s^–1^]	ε × 10^4^ [M^–1^ cm^–1^]
*sec*-Bu BODIPY	512	517	149	1.0^b^	5.09	1.96/1.3	0	11
H-H BODIPY	517	563	1578	1.0^b^	4.53	2.21/1.9	0	9.0
CN-CN BODIPY	517	566	1703	1.0^b^	4.49	2.23/1.6	0	7.0
OMe-OMe BODIPY	527	591	2053	0.94^b^	4.69	2.00/2.4	0.128	8.6
H-NMe_2_ BODIPY	530–553	653	3158	0.46^c^	4.00	1.15/1.2	1.35	2.9
OMe-NMe_2_ BODIPY	546	652	3021	0.58^c^	4.59	1.26/1.4	0.915	3.7
CN-NMe_2_ BODIPY	569	674	2932	0.12^c^	1.59	0.755/0.48^f^	5.53	3.4
Aza H-H	578	623	1299	0.18^c^	1.33	1.35/1.0	6.17	5.2
Aza CN-CN	577	628	1481	0.19^c^	1.23	1.54/1.5	6.59	6.8
Aza H-NMe_2_	663	730	1531	0.01^d^	<*n*^e^			5.2
Aza OMe-NMe_2_	663	736	1549	0.04^d^	<*n*^e^			3.3
Aza CN-NMe_2_	671	750	1743	<0.01^d^	<*n*^e^			4.8

a Values are reported as experimental/theoretical, where the theoretical value was analyzed using the Strickler–Berg relation.

b Fluorescein in 0.1 M NaOH (Φ_f_ = 0.91) was used as a reference compound for Φ_f_ determination^42^ (excitation at 491 nm, refractive index: 1.33).

c Cresyl violet in MeOH at 22 °C (Φ_f_ = 0.54) was used as a reference compound for Φ_f_ determination^42^ (excitation at 560 nm, refractive index: 1.33).

d Oxazine 1 in EtOH (Φ_f_ = 0.11) was used as a reference compound for Φ_f_ determination^42^ (excitation at 646 nm, refractive index: 1.36).

e *n* describes the lowest detectable lifetime of the instrument.

f The low-energy absorption peak was fitted to a Gaussian function and then integrated in the analysis.

g All experiments were done in toluene solution at 22 °C (refractive index for Φ_f_ determination: 1.497).

In the following section, we will investigate and discuss how the photophysical properties of the (aza)-BODIPY are differently affected when increasing or reducing electron density in the conjugated core of the dye. The electron density was manipulated by adding different electron-donating/-withdrawing substituents in the para position of the phenyl ring, where −CN represents the electron-withdrawing (pull), −OMe and −NMe_2_ represent the electron-donating (push) effect, and −H is neither (none). By making both symmetric and unsymmetrical molecules, the photophysical properties of several electron-withdrawing/-donating combinations could be investigated, e.g., none–none, pull–pull, push–none, push–push, and push–pull. The results are first presented for BODIPYs and aza-BODIPYs (see Table 1, Figures 3a–d, S1, and S2) individually and afterward discussed comparatively.

The absorption of H-H-BODIPY, which has neither an electron-withdrawing nor-donating group, has its maximum at 517 nm. By adding electron-withdrawing substituents (CN-CN-BODIPY), the absorption spectrum is not affected at all. On the contrary, when adding weakly electron-donating substituents (OMe-OMe-BODIPY), the absorption is red-shifted by about 10 nm, and the envelope of absorption broadens. By adding a strong electron-donating group in combination with a substituent having either an electron-withdrawing (CN-NMe_2_-BODIPY) or a weakly electron-donating (OMe-NMe_2_-BODIPY) as well as neither electron-withdrawing nor-donating effect (H-NMe_2_-BODIPY), the absorption is red-shifted a further 20 nm. Interestingly, the absorption envelope is now even broader. In fact, the onset of absorption on the high-energy side remains the same for all derivatives, and the redshift is solely a result of broadening toward lower energies. Although the absorption envelope depends strongly on substitution pattern, the envelope of emission of all derivatives is similar (Figure 3b). The emission maximum is affected to a larger extent for the different substitution patterns, following the same trend as the redshift/broadening of absorption. The emission is therefore not a mirror image of the absorption. The absorption maximum of the aza-BODIPYs in comparison is more red-shifted by substituents. The absorption of Aza-H-H is centered at 578 nm. Adding electron-withdrawing substituents (Aza-CN-CN) has no effect on absorption. On the contrary, having a strong electron-donating substituent (NMe_2_) red-shifts the absorption spectra with 86 nm, and a strong electron-donating in combination with an electron-withdrawing substituent (Aza-CN-NMe_2_) results in an additional redshift of 8 nm. The absorption envelope is not affected to a high degree by substitution. The emission spectra show a mirror image relationship to the absorption for all derivatives. The push–pull effect directed over the horizontal axis (as drawn in Figure 2) of these asymmetric molecule shows an unexpectedly minor change in transition energies compared to derivatives having a push–none (Aza-H-NMe_2_) or push–push (Aza-OMe-NMe_2_) substitution pattern. A previous study by Rattanopas et al. compared the effect of electron-donating groups on symmetrical (vertical) push–pull aza-BODIPYs.^17^ Having a fixed pull moiety (CN) in the para position of 1,7 phenyls and varying the electron-donating group in the para position of 3,5-phenyls, they observed a transition energy of λ_max abs_ = 853 nm for NMe_2_ as the electron-donating moiety. Thus, having the push–pull effect directed vertically shows a greater effect on the transition energy compared to having it directed horizontally.

**Figure 4:**
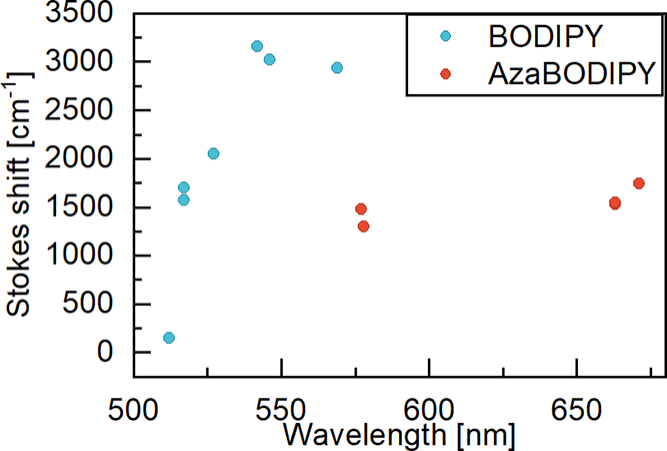
Stokes shift of BODIPY (blue) and aza-BODIPY (red) derivatives plotted against λ_max abs_ of the corresponding compounds.

It is evident that the photophysics of the two sets of molecules does not follow the same trends. The aza-BODIPY derivatives show a red-shifted absorption and emission to the same extent, resulting in a constant Stokes shift (Figure 4). On the contrary, BODIPY derivatives exhibit a more red-shifted emission maxima compared to the absorption, resulting in a substitution pattern-dependent Stokes shift. This increase in Stokes shift has previously been observed for BODIPYs carrying phenyl substituent in the 3 and 5 positions, although not to that great extent.^43^

BODIPYs are known to have very high fluorescence quantum yields (Φ_f_).^44,45^ As expected, the fluorescence quantum yields of *sec*-Bu BODIPY, H-H-BODIPY, CN-CN-BODIPY, and OMe-OMe-BODIPY are close to unity. However, for H-NMe_2_-BODIPY and OMe-NMe_2_-BODIPY, the emission quantum yield is considerably lower, and for CN-NMe_2_-BODIPY, it is a mere 12%. When introducing push and pull substituents, as discussed before, the energy of the electronic transition is lowered. The lower emission quantum yield can therefore partly be explained by the energy gap law that states that the rate of nonradiative relaxation increases exponentially with lowered transition energy.^46^ However, a part of the lower Φ_f_ of the BODIPYs having a strong electron-donating substituent is due to a reduced rate of emission. It is tempting to connect this reduced rate with the lower molar absorptivity for these molecules (as these are related). The low-energy absorption transition was therefore analyzed using the Strickler–Berg relation. A lower radiative rate was also found using this theoretical approach for the phenyl-substituted BODIPYs having strong electron-donating substituents compared to other phenyl-substituted BODIPYs (Table 1 entries 2–7).

**Figure 5:**
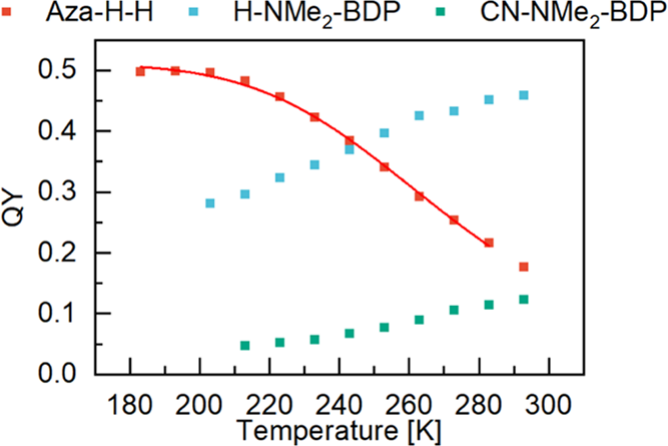
Temperature-dependent fluorescence quantum yield measurements of Aza-H-H (red), H-NMe_2_-BODIPY (blue), and CN-NMe_2_-BODIPY (green). The red line is a fit to eq 1. For the temperature-dependent absorption and emission spectra used to construct this graph, see Figures S3 and S4, respectively.

The aza-BODIPYs have, as expected due to earlier reports,^15,19^ comparatively lower fluorescence quantum yields, starting from 19% for Aza-H-H to below 1% for Aza-CN-NMe_2_. It is interesting to compare Aza-H-H and H-NMe_2_-BODIPY, as these molecules show a similar transition energy but a noticeable difference in the fluorescence quantum yields (19 vs 46%). When considering the rate constants (Table 1), it becomes clear that this reduction comes from an increased nonradiative rate in Aza-H-H. Two processes can be responsible for this increased nonradiative rate: internal conversion (IC) that is, among other things, dependent on the transition energy, and singlet-to-triplet intersystem crossing (ISC). Aza-BODIPYs are known to be more amendable for ISC compared to BODIPYs.^44^ The rate of IC is more sensitive to temperature compared to ISC, and when decreasing the temperature, the effect of IC on the fluorescence quantum yield can be lowered or sometimes even entirely removed. Figure 5 displays the temperature dependence of Φ_f_ for Aza-H-H, and the BODIPY derivatives H-NMe_2_-BODIPY and CN-NMe_2_-BODIPY, all of which absorbing and emitting in a similar energy range. While decreasing the temperature, Φ_f_ first increases but then reaches a plateau value of about 50% at 180–200 K for Aza-H-H. The temperature dependence can be accurately described using a model containing non-temperature-dependent and temperature-dependent nonradiative relaxation processes.
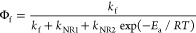
1where *E*_a_ is the activation energy, *R* is the gas constant, and *T* is the temperature. The fitted activation energy equals to about 1700 cm^–1^, which is on the order of magnitude of one vibrational excitation. This leads to the conclusion that the low Φ_f_ does not originate from a single temperature-dependent nonemissive process, such as internal conversion. However, triplet states, generated from ISC would be expected to be long-lived. Transient absorption measurements did not show any absorption features of a triplet state nor any long-lived ground-state bleach (Figure S5). This does not exclude the formation of triplet states, although it suggests that there is either no formation of triplet states or that the lifetime of the triplet states is very short. The two BODIPY derivatives on the other hand exhibit a reduction of the emission quantum yield within the same temperature range. It is thus very clear that the excited-state relaxation pathways differ considerably between BODIPYs and aza-BODIPYs having similar transition energies.

## Conclusions

We have deduced the effect of the aza-N bridge on photophysics by comparing structural (aza)-BODIPY homologues. Two sets of differently substituted (aza)-BODIPYs were synthesized utilizing a combinatorial synthetic approach. This simple yet highly efficient strategy offered us access to a library of BODIPY as well as aza-BODIPY derivatives enabling us to deduce a structure–function relationship for meso-methine bridge compared to aza-N bridge, and asymmetric push–pull systems. Replacing the meso-methine bridge with an aza-N bridge does not only result in a red-shifted transition, but the excited-state relaxation pathways were considerably affected. The effect of the 3,5-phenyl substituents on the absorption properties of BODIPYs was suppressed compared to that of aza-BODIPYs. The result could be evident in a substitution pattern-dependent Stokes shift for 3,5-substituted BODIPYs that was not observed for aza-BODIPYs. Furthermore, a clear temperature-activated nonradiative relaxation pathway was found in an aza-BODIPY but not in BODIPYS having relatively similar transition energies. Finally, we believe that the insights deduced herein on the photophysics of BODIPY derivatives will be beneficial when de novo designing dyes for specific applications.

## Experimental Section

### General Methods

All reactions were carried out under ambient conditions unless stated differently, for example, performed under N_2_ atmosphere. Glassware were oven-dried prior to use. Unless indicated otherwise, common reagents, including arylbromides, 4-bromo-3-methylanisole, XPhosPdG2, solvents, or materials, were obtained from Sigma-Aldrich Chemical Co. and used without further purification. For heating of reactions, metal heating mantles of the appropriate size of a flask were used. Dry solvents for reactions sensitive to moisture and/or oxygen were obtained through a solvent purifying system (MBRAUN SPS-800). Column chromatography was performed using silica gel (VWR 40–63 μm) unless stated otherwise. Flash chromatography was performed on a Teledyne CombiFlash EZ prep using RediSepRf columns, normal-phase silica with a mesh size of 230–400, a particle size of 40–63 μm, and a pore size of 60 Å, unless stated otherwise. ^1^H (^13^C{^1^H}) NMR spectra were recorded on a Varian 400 spectrometer (400 MHz ^1^H; 101 MHz ^13^C{^1^H}) or a BRUKER spectrometer (800 MHz ^1^H; 201 MHz ^13^C) at room temperature using CDCl_3_ (containing tetramethylsilane with 0.00 ppm as an internal reference) or DMSO-*d*_6_ as solvent. Coupling constants (*J* values) are given in hertz (Hz), and chemical shifts are reported in parts per million (ppm). Low-resolution MS was obtained from a GC/MSD System from Agilent 7820A GC System in tandem with an Agilent 5977E mass spectrometer. High-resolution MS was obtained from an Agilent 1290 Infinity LC system equipped with an autosampler in tandem with an Agilent 6520 Accurate Mass Q-TOF LC/MS. Melting points were measured using a BÜCHI Melting Point B-545 instrument. IR spectra were recorded using an INVENIO R instrument from BRUKER.

### Optical Spectroscopy

Absorption spectra were measured using a spectrophotometer (LAMBDA 950, PerkinElmer). Steady-state emission spectra, excitation spectra, and emission lifetimes were measured with a spectrofluorometer (FLS1000, Edinburgh Instrument) and are corrected using the emission correction files provided by the manufacturer. The fluorescence quantum yields were calculated using the relative method, using a standard with known emission quantum yield.^42^ Three different reference compounds were used, appropriate for the transition energy of the compound in question. The reference compounds used are referred to in Table 1. The emission spectra were first converted to the energy scale using the lambda-squared correction when assessing the emission maximum for calculating the Stokes shift. For calculating the temperature-dependent fluorescent quantum yields, the refractive index of toluene was extrapolated from literature values.^47^ For the emission lifetime measurements, the samples were excited by a 510 or 635 nm picosecond pulsed diode laser (Edinburgh Instruments) as indicated. The IRF (instrument response function) was measured using a soap and water solution having a high scattering effect. Temperature-dependent emission measurements were performed using an Oxford Instrument Optistat DN-V cryostat with N_2_ atmosphere. The temperature of the samples was controlled by a MercuryiTC temperature controller. Transient absorption measurements were performed on an Edinburgh Instrument LP 980 spectrometer equipped with an ICCD (Andor). A Spectra-Physics Nd:YAG 532 nm laser (pulse width ∼7 ns) coupled to a Spectra-Physics primoscan optical parametric oscillator (OPO) was used as the pump source.

### Synthesis and Characterization

Synthesis of (1-(*tert*-butoxycarbonyl)-4-(*sec*-butyl)-1*H*-pyrrol-2-yl)boronic acid including all prior intermediates in the route toward the compound, and characterization thereof, can be found in the SI.

### General Procedure of the Suzuki coupling

This reaction was performed under N_2_ atmosphere. The Suzuki coupling of the (1-(*tert*-butoxycarbonyl)-4-(*sec*-butyl)-1*H*-pyrrol-2-yl)boronic acid with arylbromides was executed following the reported procedure.^37^ The products of this reaction will, in the following, be called R-SCP-NBoc, where R stands for the residue in para position on the arylhalide, SCP denotes the Suzuki coupling product, and NBoc stands for the Boc protection of the pyrrole. The procedure is described taking **OMe-SCP-NBoc** as an example.

(1-(*tert*-butoxycarbonyl)-4-(*sec*-butyl)-1*H*-pyrrol-2-yl)boronic acid (267 mg, 1.00 mmol, 1.0 equiv), XPhosPdG2 (15.8 mg, 0.02 mmol, 0.02 mol %), and 4-bromo-3-methylanisole (201 mg, 1.00 mmol, 1.0 equiv), as far as the arylbromide is solid, were added to a vial, which was subsequently evacuated and refilled with N_2_ (3 cycles). THF and K_3_PO_4_ (0.5 M in H_2_O) were bubbled with N_2_ for 4 h. THF (2 mL mmol^–1^), including arylbromide in the case of the latter being liquid, was added to the solids, followed by the addition of K_3_PO_4_-solution (4 mL mmol^–1^). The reaction mixture was stirred until full conversion at room temperature. The conversion was followed via TLC (hexane/EtOAc 10% stained with vanillin) (full conversion after approximately 4 h). The reaction mixture was passed through a Celite plug and eluted with EtOAc (20 mL). Afterward, the phases were separated. The organic phase was dried over Na_2_SO_4_, and the solvent was removed under reduced pressure. The crude product was purified using flash column chromatography (SiO_2_ column, hexane/EtOAc 0–10%).

#### OMe-SCP-NBoc

The product was isolated using flash column chromatography (SiO_2_ column, hexane/EtOAc, 0–10%) as a colorless oily liquid in 72% yield (0.25 g, 0.72 mmol) from 1 mmol of (1-(*tert*-butoxycarbonyl)-4-(*sec*-butyl)-1*H*-pyrrol-2-yl)boronic acid. ^**1**^**H NMR (400 MHz, CDCl**_**3**_**)** δ 7.10 (d, *J* = 8.3 Hz, 1H), 7.07 (dd, *J* = 2.0, 0.9 Hz, 1H), 6.73 (d, *J* = 2.8 Hz, 1H), 6.69 (dd, *J* = 8.3, 2.8 Hz, 1H), 5.92 (d, *J* = 2.0 Hz, 1H), 3.79 (s, 3H), 2.52 (h, *J* = 6.8 Hz, 1H), 2.08 (s, 4H), 1.65–1.41 (m, 1H), 1.27 (s, 9H), 1.18 (d, *J* = 6.8 Hz, 3H), 0.88 (t, *J* = 7.4 Hz, 3H). ^**13**^**C {**^**1**^**H} NMR (101 MHz, CDCl**_**3**_**)** δ 159.1, 149.5, 139.2, 133.3, 131.8, 131.0, 127.8, 116.7, 114.9, 113.8, 110.0, 82.5, 55.2, 33.3, 30.4, 28.2, 20.7, 20.2, 11.9. **IR:** ν_max_/cm^–1^ 2916, 2929, 1734, 1457, 1360, 1338, 1243, 1162, 986, 846, 766. **GC/MS:***m*/*z* calcd for C_21_H_29_NO_3_ (M^+^, 100%): 343.21; found: 343.2. **HRMS: (AP+)***m*/*z* calcd For (M + H)^+^ C_21_H_30_NO_3_: 344.2226; found: 344.2233.

#### NMe_2_-SCP-NBoc

The product was isolated using flash column chromatography (SiO_2_ column, hexane/EtOAc, 0–10%) as a light yellowish oil in 82% yield (0.29 g, 0.82 mmol) from 1 mmol of (1-(*tert*-butoxycarbonyl)-4-(*sec*-butyl)-1*H*-pyrrol-2-yl)boronic acid. ^**1**^**H NMR (400 MHz, CDCl**_**3**_**)** δ 7.08–7.01 (m, 2H), 6.59–6.50 (m, 2H), 5.91 (d, *J* = 2.1 Hz, 1H), 2.93 (s, 6H), 2.51 (h, *J* = 6.9 Hz, 1H), 2.08 (s, 3H), 1.65–1.41 (m, 3H), 1.28 (s, 9H), 1.18 (d, *J* = 6.9 Hz, 3H), 0.88 (t, *J* = 7.4 Hz, 3H). ^**13**^**C {**^**1**^**H} NMR (101 MHz, CDCl**_**3**_**)** δ 150.3, 149.6, 138.2, 134.1, 131.7, 130.7, 123.7, 116.5, 113.6 (represents 2), 109.4, 82.2, 40.7, 33.3, 30.4, 27.6, 20.6, 20.5, 11.9. **IR:** ν_max_/cm^–1^ 2960, 2925, 1733, 1616, 1489, 1457, 1359, 1161, 984, 844, 766. **GC/MS:***m*/*z* calcd for C_22_H_32_N_2_O_2_ (M^+^ - C_5_H_9_O_2_): 256.19; found: 256.1. **HRMS: (AP+)***m*/*z* calcd For (M + H)^+^ C_22_H_33_N_2_O_2_: 357.2497; found: 357.2557.

#### CN–SCP-NBoc

The product was isolated using flash column chromatography (SiO_2_ column, hexane/EtOAc, 0–10%) as a yellow oil in 83% yield (0.84 g, 2.5 mmol) from 3 mmol of (1-(*tert*-butoxycarbonyl)-4-(*sec*-butyl)-1*H*-pyrrol-2-yl)boronic acid. ^**1**^**H NMR (400 MHz, CDCl**_**3**_**)** δ 7.49–7.45 (m, 2H), 7.30 (dd, *J* = 8.3, 0.4 Hz, 1H), 7.11 (dd, *J* = 2.0, 0.9 Hz, 1H), 5.99 (dd, *J* = 2.0, 0.4 Hz, 1H), 2.59–2.49 (m, 1H), 2.16 (s, 3H), 1.64–1.47 (m, 2H), 1.29 (s, 9H), 1.20 (d, *J* = 6.9 Hz, 3H), 0.89 (t, *J* = 7.4 Hz, 3H). ^**13**^**C {**^**1**^**H} NMR (101 MHz, CDCl**_**3**_**)** δ 149.0, 140.3, 139.3, 132.5, 132.3, 131.3, 130.6, 128.9, 119.1, 117.7, 114.3, 111.2, 83.4, 33.3, 30.4, 27.5, 20.7, 19.7. **IR:** ν_max_/cm^–1^ 2962, 2929, 2230, 1772, 1370, 1370, 1322, 1160, 989, 846, 768. **GC/MS:***m*/*z* calcd for C_21_H_26_N_2_O_2_ (M^+^ - C_5_H_9_O_2_): 238.14; found: 238.2. **HRMS: (AP+)***m*/*z* calcd For (M + H)^+^ C_21_H_27_N_2_O_2_: 339.2073; found: 339.2073.

#### H-SCP-NBoc

The product was isolated using flash column chromatography (SiO_2_ column, hexane/EtOAc, 0–10%) as a light yellowish oil in 89% yield (0.56 g, 1.8 mmol) from 2 mmol of (1-(*tert*-butoxycarbonyl)-4-(*sec*-butyl)-1*H*-pyrrol-2-yl)boronic acid. ^**1**^**H NMR (400 MHz, CDCl**_**3**_**)** δ 7.23–7.11 (m, 4H), 7.09 (dd, *J* = 2.0, 0.8 Hz, 1H), 5.94 (dd, *J* = 2.0, 0.4 Hz, 1H), 2.53 (h, *J* = 6.9 Hz, 1H), 2.11 (s, 3H), 1.65–1.42 (m, 2H), 1.21 (s, 9H), 1.19 (d, *J* = 6.9 Hz, 3H), 0.88 (t, *J* = 7.4 Hz, 3H). ^**13**^**C {**^**1**^**H} NMR (101 MHz, CDCl**_**3**_**)** δ 149.5, 137.7, 135.3, 133.4, 131.9, 129.9, 129.1, 127.6, 124.9, 116.8, 113.6, 82.6, 33.3, 30.4, 28.1, 20.7, 19.9, 11.9. **IR:** ν_max_/cm^–1^ 2964, 1930, 1781, 1733, 1457, 1369, 1321, 1251, 1161, 989, 766. **GC/MS:***m*/*z* calcd for C_20_H_27_NO_2_ (M^+^, 100%): 313.20; found: 313.2. **HRMS: (AP+)***m*/*z* calcd For (M + H)^+^ C_20_H_28_NO_2_: 314.2120; found: 314.2111.

### General Procedure for the Vilsmeier–Haack Reaction toward α-Formylated Pyrroles for the BODIPY Synthesis

This reaction was performed under N_2_-atmosphere, following the reported procedure.^45^ The products of this reaction will, in the following, be called R-SCP-NH-αCOH, where R stands for the residue in para position on the arylhalide, SCP denotes the Suzuki coupling product, NH denotes the free Pyrrole NH, and αCOH stands for the formylation in the second α position of the pyrrole. The procedure is described taking **H-SCP-NH-αCOH** as an example. First, the Boc-protected pyrroles were deprotected directly before they were used for the aza-BODIPY synthesis.

H-SCP-NBoc (313 mg, 1.00 mmol, 1.0 equiv) was added to the reaction vessel, which was subsequently evacuated and refilled with N_2_ (3 cycles). The starting material was dissolved in dry THF (5 mL). NaOMe (25 wt % in MeOH, 0.400 mL) was added to the reaction mixture, and the reaction was stirred until full conversion of the starting material (approximately 2 h). H_2_O was added to the reaction mixture, and the phases were separated. The aqueous phase was extracted with diethyl ether (2 × 20 mL), and the combined organic phases were washed with brine (25 mL). The solvent was subsequently removed under reduced pressure. The product H-SCP-NH was obtained as a light yellow oil and was directly used without further purification or analysis.

POCl_3_ (0.11 mL, 1.2 mmol, 1.2 equiv) was added dropwise to DMF (0.093 mL, 1.2 mmol, 1.2 equiv) at 0 °C under N_2_-atmosphere. The mixture was left to warm to room temperature and stirred at that temperature for 15–30 min or until formation of a solid. The formed Vilsmeier reagent was dissolved in DCE (1 mL), and the solution was cooled to 0 °C. H-SCP-NH (0.21 mg, 1.0 mmol, 1.0 equiv) in DCE (1 mL) was added dropwise to the solution over 10–15 min at 0 °C. After the addition, the reaction mixture was refluxed for 30 min. Afterward, the reaction was cooled down to room temperature and NaOAc (0.45 g, 5.5 mmol, 5.5 equiv) dissolved in H_2_O (4 mL) was added to the reaction mixture. Then, the two-phase mixture was refluxed for 30 min. When the reaction was cooled down to room temperature, the aqueous phase was extracted with DCM (3 × 20 mL). The combined organic phase was washed with H_2_O (1 × 20 mL), Na_2_CO_3_ (2 × 20 mL), and brine (2 × 20 mL). Afterward, the washed organic phase was dried over Na_2_SO_4_ and the solvent was removed under reduced pressure. The compound was purified using flash column chromatography (SiO_2_, hexane/EtOAc, 0–20%).

#### H-SCP-NH-αCOH

The compound was obtained as a yellow oil (0.22 g, 0.90 mmol, 90%). ^**1**^**H NMR (400 MHz, CDCl**_**3**_**)** δ 9.62 (s, 1H), 9.54 (s, 1H), 7.44–7.36 (m, 1H), 7.26–7.22 (m, 3H), 6.26 (d, *J* = 2.7 Hz, 1H), 3.05 (h, *J* = 6.9 Hz, 1H), 2.43 (s, 3H), 1.73–1.55 (m, 2H), 1.31 (d, *J* = 6.9 Hz, 3H), 0.90 (t, *J* = 7.4 Hz, 3H). ^**13**^**C {**^**1**^**H} NMR (101 MHz, CDCl**_**3**_**)** δ 177.1, 143.9, 139.4, 135.8, 131.2, 129.0, 128.6, 128.5, 126.1, 109.8, 32.1, 31.7, 22.5, 21.0, 12.3. **IR:** ν_max_/cm^–1^ 3259, 2961, 2926, 1635, 1463, 1379, 1247, 822, 761, 722. **GC/MS:***m*/*z* calcd for C_16_H_19_NO (M^+^, 100%): 241.15; found: 241.2. **HRMS: (ESI+)***m*/*z* calcd For (M + H)^+^ C_16_H_20_NO: 242.15449; found: 242.1542.

#### OMe-SCP-NH-αCOH

The compound was obtained using flash column chromatography (SiO_2_, hexane/EtOAc, 0–20%) as a red-orange oil (91 mg, 0.36 mmol, 72%) starting from 0.5 mmol of OMe-SCP-NBoc. ^**1**^**H NMR (400 MHz, CDCl**_**3**_**)** δ 9.60 (s, 1H), 9.09 (bs, 1H), 7.32 (d, *J* = 8.0 Hz, 1H), 6.81–6.73 (m, 1H), 6.20 (d, *J* = 2.7 Hz, 0H), 3.81 (s, 3H), 3.04 (h, *J* = 6.9 Hz, 1H), 2.42 (s, 3H), 1.72–1.55 (m, 1H), 1.30 (d, *J* = 6.9 Hz, 2H), 0.89 (t, *J* = 7.4 Hz, 3H). ^**13**^**C {**^**1**^**H} NMR (101 MHz, CDCl**_**3**_**)** δ 176.8, 159.6, 144.0, 139.3, 137.4, 129.8, 128.7, 123.7, 116.6, 111.6, 109.3, 55.3, 32.1, 31.7, 22.5, 21.3, 12.3. **IR:** ν_max_/cm^–1^ 3259, 2960, 2926, 1635, 1464, 1378, 1244, 1074, 1044, 815. **GC/MS:***m*/*z* calcd for C_17_H_21_NO_2_ (M^+^, 100%): 271.16; found: 271.1. **HRMS: (ESI+)***m*/*z* calcd For (M + H)^+^ C_17_H_22_NO_2_: 272.16505; found: 272.1650.

#### CN–SCP-NH-αCOH

The compound was obtained using flash column chromatography (SiO_2_, hexane/EtOAc, 0–20%) as an orange-yellow oil (0.48 g, 1.8 mmol, 90%) starting from 2.0 mmol of CN–SCP-NBoc. ^**1**^**H NMR (400 MHz, CDCl**_**3**_**)** δ 9.69 (s, 1H), 9.22 (bs, 1H), 7.58–7.47 (m, 3H), 6.35 (dd, *J* = 2.8, 0.5 Hz, 1H), 3.06 (h, *J* = 6.9 Hz, 1H), 2.49 (s, 3H), 1.76–1.54 (m, 2H), 1.32 (d, *J* = 6.9 Hz, 3H), 0.89 (t, *J* = 7.4 Hz, 3H). ^**13**^**C {**^**1**^**H} NMR (101 MHz, CDCl**_**3**_**)** δ 177.7, 143.5, 136.8, 136.5, 135.4, 134.7, 129.8, 128.9, 118.5, 111.8, 110.8, 32.0, 31.6, 22.5, 21.1, 12.3. **IR:** ν_max_/cm^–1^ 3272, 2960, 2925, 2229, 1653, 1636, 1248, 820. **GC/MS:***m*/*z* calcd for C_17_H_18_N_2_O (M^+^, 100%): 266.14; found: 266.1. **HRMS: (AP+)***m*/*z* calcd For (M + H)^+^ C_17_H_19_N_2_O: 267.1498; found: 267.1513.

### General Procedure for the Synthesis of Aza-BODIPY Derivatives

The aza- BODIPY derivatives were synthesized following the reported procedure.^39,48^ Taking Aza-H-NMe_2_ as an example, first, the Boc-protected pyrroles were deprotected directly before they were used for the aza-BODIPY synthesis.

H-SCP-NBoc (0.19 g, 0.60 mmol, 1.0 equiv) was added to the reaction vessel, which was subsequently evacuated and refilled with N_2_ (3 cycles). The starting material was dissolved in dry THF (3 mL). NaOMe (25 wt % in MeOH, 0.25 mL) was added to the reaction mixture, and the reaction was stirred until full conversion of the starting material (approximately 2 h). H_2_O was added to the reaction mixture, and the phases were separated. The aqueous phase was extracted with diethyl ether (2 × 20 mL), and the combined organic phases were washed with brine (25 mL). The solvent was subsequently removed under reduced pressure. The product H-SCP-NH was obtained as a light yellow oil and was directly used without further purification or analysis.

NMe_2_-SCP-NBoc (0.22 g, 0.60 mmol, 1.0 equiv) was added to the reaction vessel, which was subsequently evacuated and refilled with N_2_ (3 cycles). The starting material was dissolved in dry THF (3 mL). NaOMe (25 wt % in MeOH, 0.25 mL) was added to the reaction mixture, and the reaction was stirred until full conversion of the starting material (approximately 2 h). H_2_O was added to the reaction mixture, and the phases were separated. The aqueous phase was extracted with diethyl ether (2 × 20 mL), and the combined organic phases were washed with brine (25 mL). The solvent was subsequently removed under reduced pressure. The product NMe_2_-SCP-NH was obtained as a light yellow oil and was directly used without further purification or analysis.

H-SCP-NH was put in a vial together with NaNO_2_ (41 mg, 0.60 mmol, 1.0 equiv). The vial was evacuated and subsequently refilled with N_2_ (3 cycles). Acetic acid (6 mL) was added to the reaction vessel, and the mixture was stirred for 45 minutes at room temperature. NMe_2_-SCP-NH in acetic anhydride (2.4 mL) was added to the reaction mixture, followed by an immediate color change. The reaction mixture was stirred for 30 min at room temperature and then for 30 min at 80 °C. After cooling down the reaction, cold saturated NaHCO_3_ (300 mL) was added slowly to the reaction mixture. The quenched reaction mixture was extracted with DCM (3 × 100 mL). The combined organic layers were dried over Na_2_SO_4_, and the solvent was removed under reduced pressure.

The resulting dark purple solid was dissolved in DCE (10 mL). Et_3_N (2.5 mL) was added at 0 °C, and the mixture was stirred for 15 min at that temperature, followed by the addition of BF_3_·OEt_2_ (2.4 mL) at 0 °C. The reaction mixture was left to warm to r.t. and stirred overnight at ambient temperature. Afterward, the mixture was heated to 80 °C and stirred for 30 min. After cooling to room temperature, H_2_O (20 mL) was added to the reaction mixture and the two-layered mixture was stirred for 2 h. The reaction mixture was extracted with DCM (3 × 20 mL mmol^–1^). The combined organic layers were washed with H_2_O (1 × 50 mL mmol^–1^) and NaHCO_3_ (2 × 50 mL mmol^–1^) and then dried over Na_2_SO_4_. The solvent was removed under reduced pressure.

#### Aza-H-NMe_2_

The crude product was purified using flash column chromatography (column: interchim F0025, 24 g, 15 μm spherical SiO_2_) using cyclohexane/EtOAc, 0–10% as eluent. The purified product was afforded as a blue-black solid (52 mg, 0.098 mmol, 16%). ^**1**^**H NMR (400 MHz, CDCl**_**3**_**)** δ 7.70 (d, *J* = 8.5 Hz, 1H), 7.49 (d, *J* = 7.4 Hz, 1H), 7.29–7.13 (m, 3H), 6.55–6.47 (m, 2H), 6.37 (s, 1H), 6.20 (s, 1H), 3.18–3.03 (m, 2H), 2.95 (s, 6H), 2.37 (s, 3H), 2.30 (s, 3H), 1.82–1.61 (m, 4H), 1.32 (dd, *J* = 6.9, 0.8 Hz, 6H), 0.95 (tdd, *J* = 7.4, 4.2, 1.8 Hz, 6H). ^**13**^**C {**^**1**^**H} NMR (101 MHz, CDCl**_**3**_**)** δ 162.9, 155.6, 154.0, 151.5, 150.8, 145.7, 143.2, 138.4, 136.7, 133.0, 132.1, 129.9, 128.9, 125.0, 122.1, 119.5, 118.4, 113.7, 110.4, 109.2, 40.0, 33.0, 32.9, 30.9, 30.5, 22.0, 21.2, 20.8, 20.5, 12.2, 12.1. **HRMS: (ESI+)***m*/*z* calcd For (M + H)^+^ C_32_H_40_BF_2_N_4_: 528.3350; found: 528.3348. **IR:** ν_max_/cm^–1^ 2959, 2923, 2865, 1601, 1500, 1471, 1384, 1293, 1109, 1046, 743. **Mp:** 156–158 °C.

#### Aza-OMe-NMe_2_

The product was isolated as a blue-black solid by column chromatography on SiO_2_, hexane/EtOAc, 5–20%, and a second column on SiO_2_, cyclohexane/EtOAc, 10% (22 mg, 0.039 mmol, 9%) starting with 0.43 mmol of OMe-SCP-NBoc as the first pyrrole added and 0.43 mmol of NMe_2_-SCP-NBoc as the second pyrrole added. ^**1**^**H NMR (400 MHz, CDCl**_**3**_**)** δ 7.66 (d, *J* = 8.7 Hz, 1H), 7.49 (d, *J* = 8.2 Hz, 1H), 6.75–6.70 (m, 2H), 6.55–6.48 (m, 2H), 6.34 (s, 1H), 6.19 (s, 1H), 3.78 (s, 3H), 3.14–3.03 (m, 2H), 2.97 (s, 6H), 2.35 (s, 3H), 2.29 (s, 3H), 1.80–1.63 (m, 4H), 1.33–1.23 (m, 12H). ^**13**^**C {**^**1**^**H} NMR (201 MHz, CDCl**_**3**_**)** δ 162.2, 160.3, 156.6, 153.7, 151.6, 151.3, 145.6, 143.7, 138.7, 138.4, 132.1, 131.7, 125.6, 121.9, 119.9, 119.3, 115.9, 113.9, 110.7, 109.4, 55.4, 40.3, 33.2, 31.0, 30.9, 30.8, 30.6, 29.9, 22.1, 21.4, 21.3, 21.2, 21.1, 21.1, 12.4, 12.3. **HRMS:** (ESI+) *m*/*z* calcd For (M + H)^+^ C_33_H_42_BF_2_N_4_O: 558.3456; found: 558.3448. **IR:** ν_max_/cm^–1^ 2959, 2924, 1604, 1540, 1507, 1481, 1395, 1296, 1244, 1114, 1058, 749. **Mp:** 131–134 °C.

#### Aza-CN-NMe_2_

The product was isolated as a blue-black solid by column chromatography on SiO_2_, hexane/EtOAc, 5–20%, and a second column on SiO_2_, cyclohexane/EtOAc, 10% (0.14 g, 0.25 mmol, 25%) starting with 1.0 mmol of CN–SCP-NBoc as the first pyrrole added and 1.0 mmol NMe_2_-SCP-NBoc as the second pyrrole added. ^**1**^**H NMR (800 MHz, CDCl**_**3**_**)** δ 7.78 (d, *J* = 8.8 Hz, 1H), 7.61 (d, *J* = 7.9 Hz, 1H), 7.50 (d, *J* = 1.7 Hz, 1H), 7.47 (dd, *J* = 7.9, 1.7 Hz, 1H), 6.56 (dd, *J* = 8.8, 2.7 Hz, 1H), 6.53 (d, *J* = 2.7 Hz, 1H), 6.47 (s, 1H), 6.17 (s, 1H), 3.17–3.07 (m, 2H), 3.02 (s, 6H), 2.40 (s, 3H), 2.34 (s, 3H), 1.82–1.66 (m, 4H), 1.34 (dd, *J* = 7.0, 3.9 Hz, 6H), 1.00–0.93 (m, 6H). ^**13**^**C {**^**1**^**H} NMR (201 MHz, CDCl**_**3**_**)** δ 165.5, 155.5, 152.2, 150.4, 149.6, 147.0, 142.7, 139.2, 138.7, 138.4, 133.4, 132.6, 131.2, 128.9, 123.5, 119.2, 119.0, 117.1, 114.1, 112.4, 109.4, 40.2, 33.3, 33.2, 33.2, 33.1, 31.2, 31.0, 30.6, 30.4, 29.9, 22.4, 21.6, 21.5, 21.0, 20.8, 20.5, 12.4, 12.2. **HRMS:** (ESI+) *m*/*z* calcd For (M + H)^+^ C_33_H_39_BF_2_N_5_: 553.3303; found: 553.3286. **IR:** ν_max_/cm^–1^ 2959, 2923, 2232, 1600, 1539, 1500, 1482, 1457, 1393, 1300, 1203, 1114, 1057. **Mp:** 169–171 °C.

#### Aza-CN-CN

The product was isolated as a purple-green shining solid by flash column chromatography (interchim F0025, 24 g, 15 μm spherical SiO_2_) using cyclohexane/EtOAc, 0–10% as an eluent (15 mg, 0.028 mmol, 6%) starting with 0.50 mmol of CN–SCP-NBoc as the first pyrrole added and 0.50 mmol CN–SCP-NBoc as the second pyrrole added. ^**1**^**H NMR (800 MHz, CDCl**_**3**_**)** δ 7.54 (d, *J* = 7.9 Hz, 2H), 7.52 (s, 2H), 7.49 (dd, *J* = 7.9, 1.7 Hz, 2H), 6.31 (s, 2H), 3.16 (hd, *J* = 7.0, 2.8 Hz, 2H), 2.33 (s, 6H), 1.82–1.69 (m, 4H), 1.36 (d, *J* = 7.0 Hz, 7H), 0.98 (td, *J* = 7.4, 3.2 Hz, 7H). ^**13**^**C {**^**1**^**H} NMR (201 MHz, CDCl**_**3**_**)** δ 158.6, 156.1, 145.1, 138.3, 136.6, 133.7, 130.5, 129.2, 120.0, 118.7, 113.6, 33.5, 30.9, 30.8, 29.9, 21.2, 21.1, 20.4, 12.3. **HRMS:** (ESI+) *m*/*z* calcd For (M + H)^+^ C_32_H_33_BF_2_N_5_: 535.2833; found: 535.2829. **IR:** ν_max_/cm^–1^ 2959, 2923, 2229, 1539, 1516, 1482, 1457, 1399, 1301, 1113, 1058, 743. **Mp:** 208–210 °C.

#### Aza-H-H

The product was isolated as a purple-green shining solid by flash column chromatography (interchim F0025, 24 g, 15 μm spherical SiO_2_) using cyclohexane/EtOAc, 0–10% as an eluent, and an additional preparative TLC on SiO_2_, cyclohexane/EtOAc, 6% (13 mg, 0.027 mmol, 3%) starting from 0.90 mmol of H-SCP-NBoc as the first pyrrole added and 0.90 mmol of H-SCP-NBoc as the second pyrrole added. ^**1**^**H NMR (400 MHz, CDCl**_**3**_**)** δ 7.45 (d, *J* = 7.6 Hz, 2H), 7.28–7.22 (m, 2H), 7.21–7.13 (m, 4H), 6.24 (s, 2H), 3.10 (h, *J* = 7.0 Hz, 2H), 2.27 (s, 6H), 1.77–1.67 (m, 4H), 1.31 (d, *J* = 7.0 Hz, 6H), 0.94 (td, *J* = 7.4, 1.7 Hz, 6H). ^**13**^**C {**^**1**^**H} NMR (101 MHz, CDCl**_**3**_**)** δ 160.2, 154.2, 144.5, 136.4, 132.1, 129.7, 129.5, 125.2, 120.0, 33.1, 31.4, 30.7, 20.9, 12.1. **HRMS:** (ESI+) *m*/*z* calcd For (M + H)^+^ C_30_H_35_BF_2_N_3_: 485.2928; found: 485.2923. **IR:** ν_max_/cm^–1^ 2959, 2923, 1539, 1516, 1482, 1457, 1399, 1301, 1113, 1058, 743. **Mp:** 90–92 °C

### General Procedure for the Synthesis of Unsymmetrical BODIPY Derivatives

This reaction was performed under N_2_ atmosphere. Taking **H-NMe**_**2**_**-BODIPY** as an example, NMe_2_-SCP-NBoc (0.29 g, 0.80 mmol, 1.0 equiv) was added to the reaction vessel, which was subsequently evacuated and refilled with N_2_ (3 cycles). The starting material was dissolved in dry THF (3 mL). NaOMe (25 wt % in MeOH, 0.25 mL) was added to the reaction mixture, and the reaction was stirred until full conversion of the starting material (approximately 2 h). H_2_O was added to the reaction mixture, and the phases were separated. The aqueous phase was extracted with diethyl ether (2 × 20 mL), and the combined organic phases were washed with brine (25 mL). The solvent was subsequently removed under reduced pressure. The product NMe_2_-SCP-NH was obtained as a light yellow oil and was used directly without further purification.

H-SCP-NH-αCOH (0.19 g, 0.80 mmol, 1.0 equiv) was dissolved in DCM (2 mL), and the solution was cooled down to 0 °C. A catalytic amount of acetic acid was added to the cooled solution, and afterward, the reaction mixture was stirred at 0 °C for 5 min. NMe_2_-SCP-NH in DCM (2 mL) was added to the reaction mixture at 0 °C, accompanied by an immediate change in color. The reaction was thereafter stirred for 10 min at 0 °C, then warmed to room temperature, and stirred at that temperature for 4 h. Afterward, the reaction was cooled down to 0 °C. Et_3_N (1.2 mL) was added to the reaction mixture, which was then stirred for 15 min before BF_3_·OEt_2_ (1.0 mL) was added dropwise. The reaction was then warmed to room temperature where it was stirred for 15 min. Afterward, the reaction was cooled down once again to 0 °C, where the addition of first Et_3_N (1.2 mL) and the BF_3_·OEt_2_ (1.0 mL) was repeated in the same manner as before. After the second addition of BF_3_·OEt_2_, the solution was stirred at room temperature overnight. The solution was passed through a silica plug after using hexane/EtOAc (20–100%) as an eluent. The resulting solution was evaporated onto Celite and purified.

#### H-NMe_2_-BODIPY

The product was isolated as a purple solid after purification via column chromatography (3 ×) on SiO_2_ using cyclohexane/EtOAc (5–10%) and one preparative TLC on SiO_2_, cyclohexane/EtOAc, 6% (32 mg, 0.061 mmol, 8%). ^**1**^**H NMR (400 MHz, CDCl**_**3**_**)** δ 7.45 (dd, *J* = 8.0, 6.0 Hz, 2H), 7.31 (s, 1H), 7.25–7.11 (m, 3H), 6.53 (dd, *J* = 8.5, 2.7 Hz, 1H), 6.50 (d, *J* = 2.7 Hz, 1H), 6.22 (s, 1H), 6.16 (s, 1H), 2.93 (s, 6H), 2.92–2.82 (m, 2H), 2.26 (s, 3H), 2.24 (s, 3H), 1.67 (p, *J* = 7.4 Hz, 4H), 1.31 (dd, *J* = 6.9, 1.6 Hz, 6H), 0.95 (tdd, *J* = 7.5, 2.1, 1.4 Hz, 6H). ^**13**^**C {**^**1**^**H} NMR (101 MHz, CDCl**_**3**_**)** δ 159.8, 156.0, 151.9, 150.7, 150.1, 137.4, 136.8, 133.9, 133.4, 132.5, 131.1, 130.0, 129.6, 128.5, 124.9, 120.9, 120.6, 118.7, 116.7, 113.4, 109.1, 40.2, 32.9, 31.4, 31.23, 29.7, 22.3, 22.1, 21.2, 20.3, 12.2. **HRMS:** (ESI+) *m*/*z* calcd For (M + H)^+^ C_33_H_41_BF_2_N_3_: 527.3398; found: 527.3397. **IR:** ν_max_/cm^–1^ 2960, 2921, 1594, 1502, 1481, 1200, 1185, 1133, 1117. **Mp:** 124–126 °C.

#### OMe-NMe_2_-BODIPY

The product was isolated as a purple solid after purification via column chromatography (3 ×) on SiO_2_ using cyclohexane/EtOAc (0–10%) and one preparative TLC on SiO_2_, cyclohexane/EtOAc, 6% (12 mg, 0.021 mmol, 4%) starting with 0.57 mmol of OMe-SCP-NH-αCOH as the formylated pyrrole and 0.57 mmol of NMe_2_-SCP-NH as the pyrrole. ^**1**^**H NMR (400 MHz, CDCl**_**3**_**)** δ 7.45 (d, *J* = 8.4 Hz, 1H), 7.43–7.38 (m, 1H), 7.28 (s, 1H), 6.71 (dt, *J* = 4.4, 2.4 Hz, 2H), 6.54 (dd, *J* = 8.4, 2.8 Hz, 1H), 6.50 (d, *J* = 2.8 Hz, 1H), 6.20 (s, 1H), 6.15 (s, 1H), 3.77 (s, 3H), 2.93 (s, 6H), 2.92–2.81 (m, 2H), 2.26 (s, 3H), 2.23 (s, 3H), 1.66 (p, *J* = 7.4 Hz, 1H), 1.29 (d, *J* = 6.9 Hz, 6H), 0.93 (tt, *J* = 7.4, 1.6 Hz, 6H). ^**13**^**C {**^**1**^**H} NMR (101 MHz, CDCl**_**3**_**)** δ 159.6, 156.3, 150.7, 150.3, 138.4, 137.4, 131.3, 131.1, 125.8, 120.7, 118.5, 117.1, 115.3, 113.4, 110.3, 109.1, 55.1, 40.2, 32.8, 32.8, 31.3, 31.3, 31.2, 31.2, 29.7, 22.2, 22.2, 22.2, 22.1, 21.2, 20.7, 12.2, 12.2. **HRMS:** (ESI+) *m*/*z* calcd For (M + H)^+^ C_34_H_43_BF_2_N_3_O: 557.3504; found: 557.3500. **IR:** ν_max_/cm^–1^ 2960, 2922, 1594, 1506, 1486, 1457, 1396, 1187, 1133. **Mp:** 127–130 °C.

#### OMe-OMe-BODIPY

The symmetrical OMe-OMe BODIPY was here isolated as a side product. The compound was isolated as a pink solid after purification via column chromatography (2 ×) on SiO_2_ using cyclohexane/EtOAc (0–10%) as an eluent (8.1 mg, 0.015 mmol, 5%) starting with 0.57 mmol of OMe-SCP-NH-αCOH as the formylated pyrrole and 0.57 mmol of NMe_2_-SCP-NH as the pyrrole. ^**1**^**H NMR (400 MHz, CDCl**_**3**_**)** δ 7.40 (d, *J* = 9.4 Hz, 2H), 7.33 (s, 1H), 6.76–6.66 (m, 4H), 6.21–6.15 (m, 2H), 2.89 (h, *J* = 6.9 Hz, 2H), 2.24 (s, 6H), 1.72–1.60 (m, 4H), 1.30 (d, *J* = 6.8 Hz, 6H), 0.94 (td, *J* = 7.4, 1.4 Hz, 6H). ^**13**^**C {**^**1**^**H} NMR (101 MHz, CDCl**_**3**_**)** δ 159.8, 157.7, 151.4, 138.3, 133.2, 131.2, 125.4, 121.5, 117.8, 115.4, 110.3, 55.1, 32.9, 31.2, 22.2, 20.7, 12.2. **HRMS:** (AP+) *m*/*z* calcd For (M + Na)^+^ C_33_H_39_BF_2_N_2_O_2_Na: 566.3007; found: 566.3021. **IR:** ν_max_/cm^–1^ 2960, 2923, 1595, 1485, 1462, 1398, 1296, 1247, 1185, 1135, 1123. **Mp:** 148–151 °C.

#### CN-NMe_2_-BODIPY

The product was isolated as a purple solid after purification via flash chromatography (3 ×, Interchim, 25 and 40 g, spherical silica gel, different particle sizes) using cyclohexane/EtOAc (0–10%) and one column chromatography using AlOx as the stationary phase and cyclohexane/EtOAc (10%) as an eluent (0.12 g, 0.21 mmol, 21%) starting from 1.0 mmol of CN–SCP-NH-αCOH as the formylated pyrrole and 1.0 mmol of NMe_2_-SCP-NH as the pyrrole. ^**1**^**H NMR (400 MHz, CDCl**_**3**_**)** δ 7.53 (d, *J* = 7.6 Hz, 1H), 7.49–7.39 (m, 3H), 7.30 (s, 1H), 6.55–6.46 (m, 2H), 6.25 (s, 1H), 6.12 (s, 1H), 2.93 (s, 6H), 2.87 (qd, *J* = 6.9, 4.4 Hz, 2H), 2.25 (d, *J* = 2.6 Hz, 6H), 1.72–1.58 (m, 4H), 1.29 (d, *J* = 6.9 Hz, 6H), 0.92 (tdd, *J* = 7.4, 5.5, 1.5 Hz, 6H). ^**13**^**C {**^**1**^**H} NMR (101 MHz, CDCl**_**3**_**)** δ 161.7, 153.3, 151.7, 150.9, 149.4, 138.5, 137.4, 134.8, 133.0, 132.1, 131.1, 128.6, 121.0, 119.9, 119.6, 119.0, 115.6, 113.3, 111.9, 108.9, 40.1, 32.8, 31.4, 31.1, 22.3, 22.0, 21.3, 20.2, 12.1. **HRMS:** (ESI+) *m*/*z* calcd For (M + H)^+^ C_34_H_40_BF_2_N_4_: 552.3350; found: 552.3350. **IR:** ν_max_/cm^–1^ 2963, 2927, 2230, 1681, 1591, 1483, 1398, 1191, 1123. **Mp:** 116–120 °C.

### General Procedure for the Synthesis of Symmetrical BODIPY Derivatives

This reaction was performed under N_2_ atmosphere, following the reported procedure.^45^ Taking the synthesis of **CN-CN-BODIPY** as an example,

CN–SCP-NH-αCOH (0.27 g, 1.0 mmol, 1.0 equiv) was dissolved in DCM/pentane (2/1 mixture, 2.5 mL), and the solution was cooled down to 0 °C. POCl_3_ (0.16 mL, 2.0 mmol, 2.0 equiv) was added dropwise over a period of 3 min. The resulting mixture was stirred at room temperature for 2.5 h. Et_3_N (0.84 mL, 6.0 mmol, 6.0 equiv) was added dropwise over 5 min at room temperature, and the reaction mixture was stirred for 15 min before cooling the solution down to 0 °C. BF_3_·OEt_2_ (1.1 mL, 9.0 mmol, 9.0 equiv) was added dropwise at 0 °C, and the reaction mixture was afterward stirred at room temperature for 30 min. Repeatedly, Et_3_N (0.84 mL, 6.0 mmol, 6.0 equiv) was added dropwise over 5 min at room temperature and the reaction mixture was stirred for 15 min before cooling the solution down to 0 °C. BF_3_·OEt_2_ (1.1 mL, 9.0 mmol, 9.0 equiv) was added dropwise at 0 °C, and the reaction mixture was then stirred at room temperature overnight. The reaction mixture was poured in Et_2_O (100 mL), and the organic phase was washed with H_2_O (3 × 50 mL). The organic phase was dried over Na_2_SO_4_, and the solvent was removed under reduced pressure. The product was purified via column chromatography using SiO_2_ as a stationary phase and hexane/EtOAc 10–20% as an eluent.

#### CN-CN-BODIPY

The product was isolated as an orange solid after purification (32 mg, 0.06 mmol, 12%). ^**1**^**H NMR (400 MHz, CDCl**_**3**_**)** δ 7.51–7.41 (m, 7H), 6.21 (s, 2H), 2.93 (h, *J* = 6.9 Hz, 2H), 2.25 (s, 6H), 1.75–1.62 (m, 4H), 1.32 (d, *J* = 6.9 Hz, 6H), 0.95 (td, *J* = 7.4, 1.4 Hz, 6H). ^**13**^**C {**^**1**^**H} NMR (101 MHz, CDCl**_**3**_**)** δ 155.7, 153.2, 138.2, 137.3, 133.2, 130.6, 128.8, 123.6, 118.7, 117.1, 112.7, 33.1, 31.3, 22.1, 20.1, 12.2. **HRMS:** (ESI+) *m*/*z* calcd For (M + Na)^+^ C_33_H_33_BF_2_N_4_Na: 556.2700; found: 556.2699. **IR:** ν_max_/cm^–1^ 2961, 2923, 2230, 1612, 1589, 1483, 1401, 1220, 1189, 1143, 1123. **Mp:** 178–180 °C.

#### H-H-BODIPY

The product was isolated as an orange solid (79 mg, 0.16 mmol, 38%) after purification via column chromatography using SiO_2_ as a stationary phase and hexane/EtOAc, 10–20%, as an eluent starting with 0.84 mmol of H-SCP-NH-αCOH. ^**1**^**H NMR (400 MHz, CDCl**_**3**_**)** δ 7.46–7.38 (m, 3H), 7.28–7.12 (m, 6H), 6.21 (s, 2H), 2.93 (h, *J* = 6.5 Hz, 2H), 2.25 (s, 6H), 1.69 (pd, *J* = 7.3, 1.7 Hz, 4H), 1.33 (dt, *J* = 6.9, 1.4 Hz, 6H), 0.97 (tdt, *J* = 7.4, 2.1, 1.1 Hz, 6H). ^**13**^**C {**^**1**^**H} NMR (101 MHz, CDCl**_**3**_**)** δ 157.9, 151.8, 136.7, 133.2, 132.9, 129.8, 129.7, 128.8, 125.0, 122.2, 117.5, 33.0, 31.3, 22.2, 20.3, 12.2. **HRMS:** (ESI+) *m*/*z* calcd For (M + NH_4_)^+^ C_31_H_39_BF_2_N_3_: 501.3241; found: 501.3241. **IR:** ν_max_/cm^–1^ 2957, 2916, 2850, 1609, 1593, 1473, 1457, 1393, 1207, 1183, 1136, 1115, 760. **Mp:** 179–181 °C

## Supporting Information Available

Additional spectroscopic and computational data as well as synthetic route toward pyrrole boronic acid and NMR spectra (PDF)
