# Supplementary material for: Effect of the Aza-N-Bridge and Push–Pull Moieties: A Comparative Study between BODIPYs and Aza-BODIPYs
Source: J Org Chem. 2022 Feb 21;87(5):2569–79. doi: 10.1021/acs.joc.1c02525 (PMC8902755; doi:10.1021/acs.joc.1c02525)
Supplement: Supplementary file 1 — jo1c02525_si_001.pdf [file jo1c02525_si_001.pdf]

Supporting Information for:

The effect of the aza-N-bridge and push-pull moieties, a comparative study between BODIPYs and aza-BODIPYs

Clara Schäfer, Jürgen Mony, Thomas Olsson and Karl Börjesson*

Department of Chemistry and Molecular Biology, University of Gothenburg, Kemivägen 10, 41296 Gothenburg, Sweden

karl.borjesson@gu.se

Contents

Supplementary Optical Spectroscopy results S1

Absorption, Excitation and Emission spectra of BODIPY derivatives S1

Absorption, Excitation and Emission spectra of aza-BODIPY derivatives S2

Temperature dependent absorption and emission measurements S3

Transient Absorption S4

Time correlated single photon counting S5

Synthesis of the pyrrole boronic acid S7

Synthesis of the pyrrole boronic acid S7

Synthesis of the pyrrole derivatives S9

References S10

Appendix : ^{1}H and $^{13}C\{^{1}H\}$ spectra of novel compounds S11

Supplementary Optical Spectroscopy results

Absorption, Excitation and Emission spectra of BODIPY derivatives

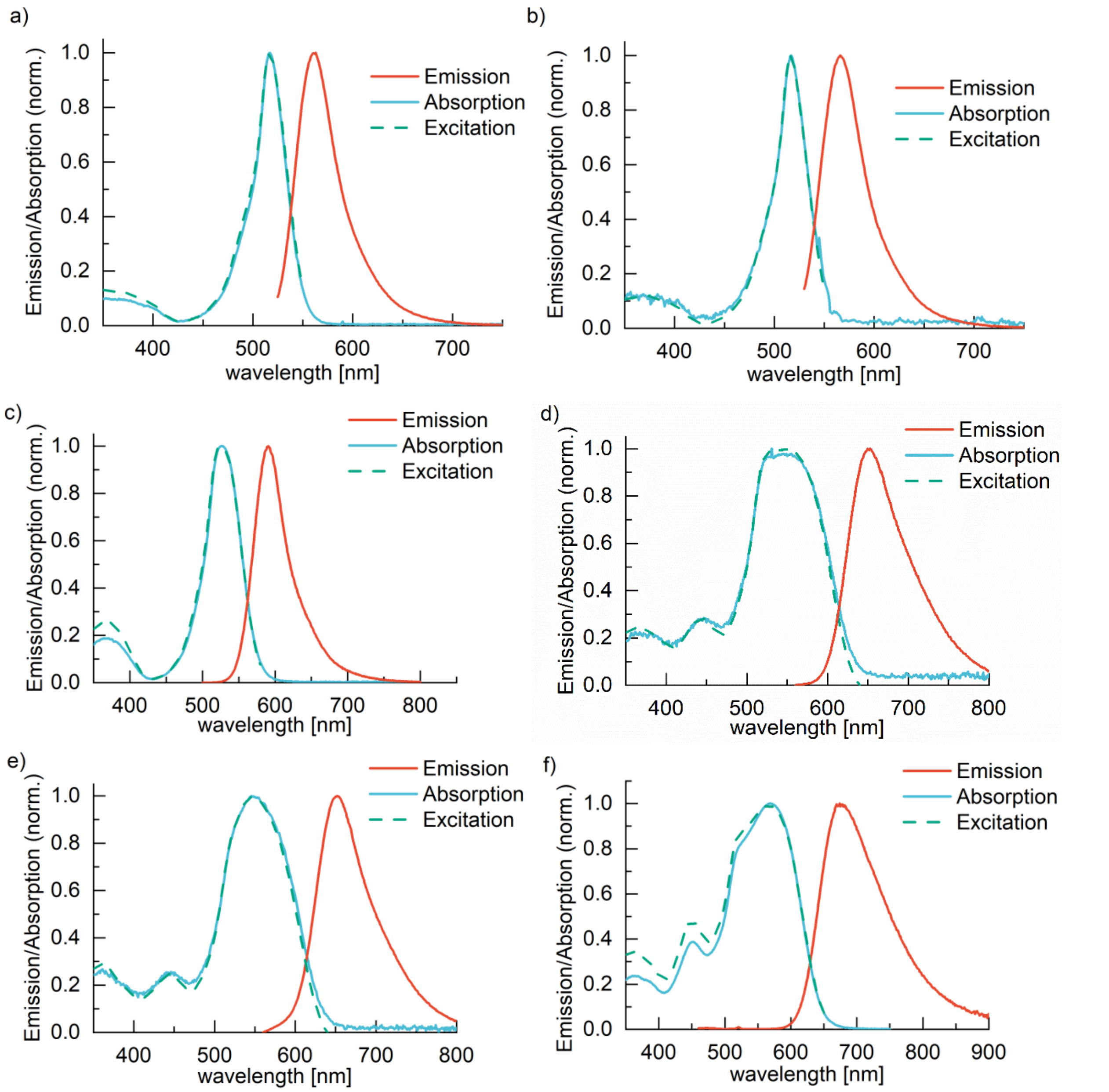

Figure S1. Normalized absorption (blue), excitation (dashed green) and emission (red) of H-H-BODIPY (a), CN-CN-BODIPY (b), OMe-OMe-BODIPY(c), H-NMe2-BODIPY (d), OMe-NMe2-BODIPY (e) and CN-NMe2-BODIPY (f). All spectra were recorded in solution, where toluene was used as solvent.

Absorption, Excitation and Emission spectra of aza-BODIPY derivatives

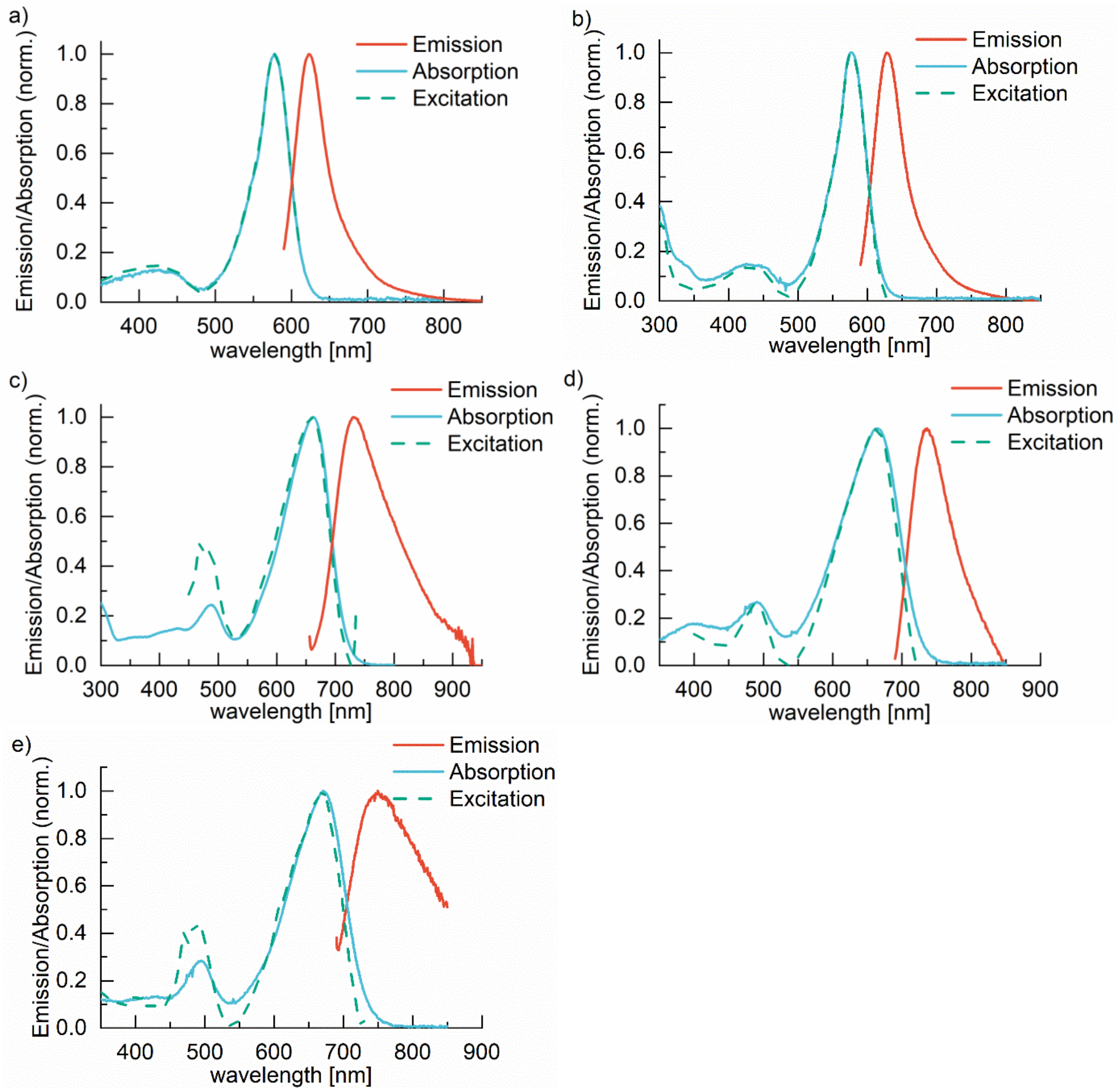

Figure S2. Normalized absorption (blue), excitation (dashed green) and emission (red) of Aza-H-H (a), Aza-CN-CN (b), Aza-H-NMe_2-BODIPY (c), Aza-OMe-NMe_2 (d) and Aza-CN-NMe_2 (e). All spectra were recorded in solution where toluene was used as solvent.

Temperature dependent absorption and emission measurements

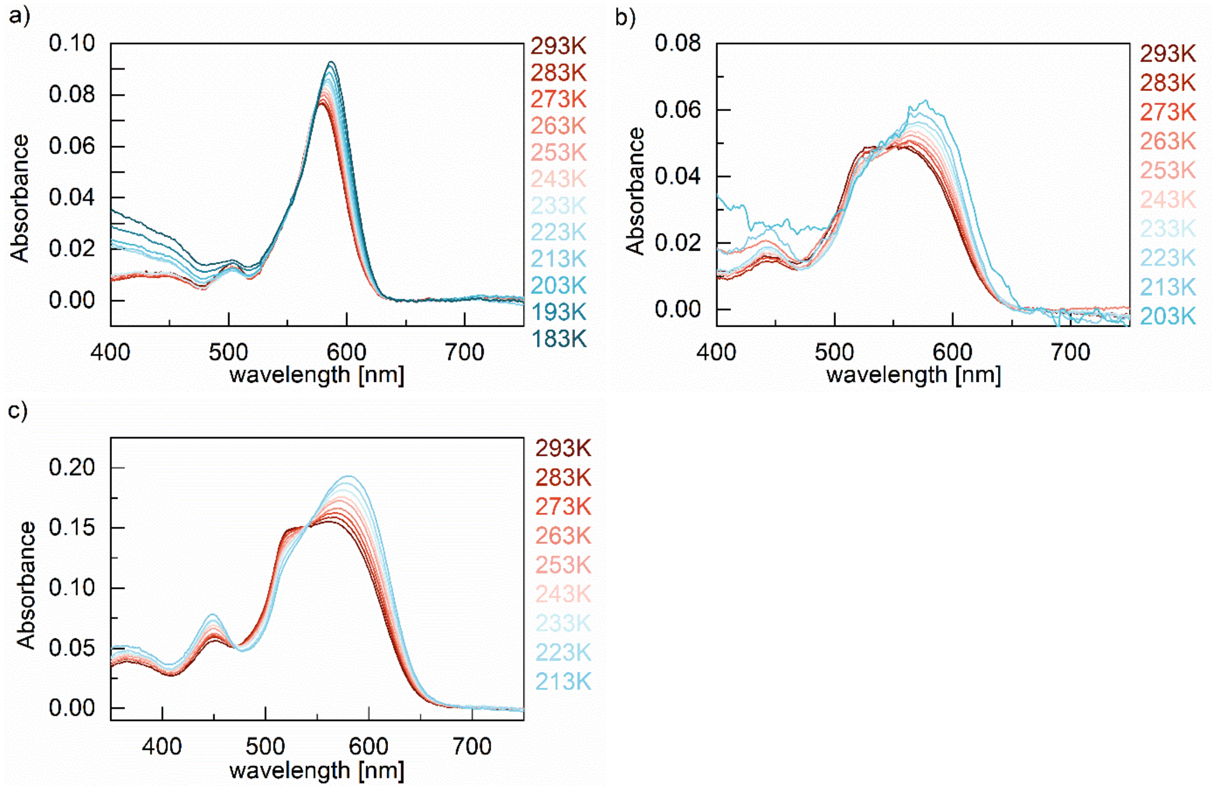

Figure S3. Temperature dependent absorption of Aza-H-H (a), H-NMe$_2$-BODIPY (b) and CN-NMe$_2$-BODIPY (c). The measurements were taken of the dyes in solution, where dry toluene was used as a solvent.

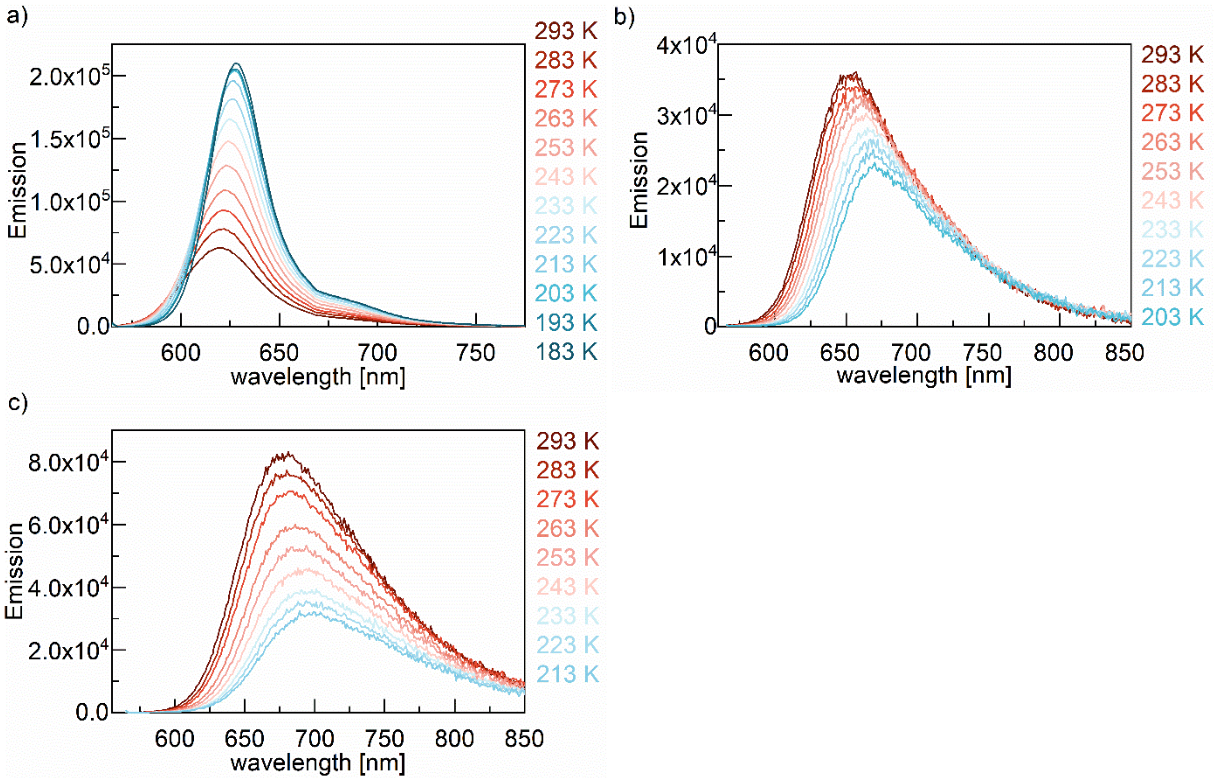

Figure S4. Temperature dependent emission of Aza-H-H (a), H-NMe$_2$-BODIPY (b) and CN-NMe$_2$-BODIPY (c). The measurements were taken of the dyes in solution, where dry toluene was used as a solvent.

Transient Absorption

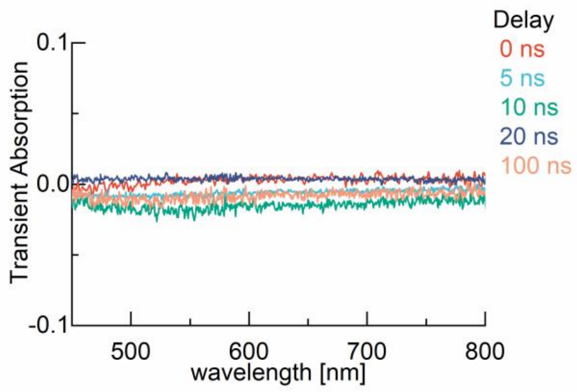

Figure S5. Transient absorption of Aza-H-H in solution, where degassed and dry toluene was used as a solvent and the sample was prepared in a glovebox. Neither ground state bleach nor triplet state absorption could be seen after the pump pulse had declined.

Time correlated single photon counting

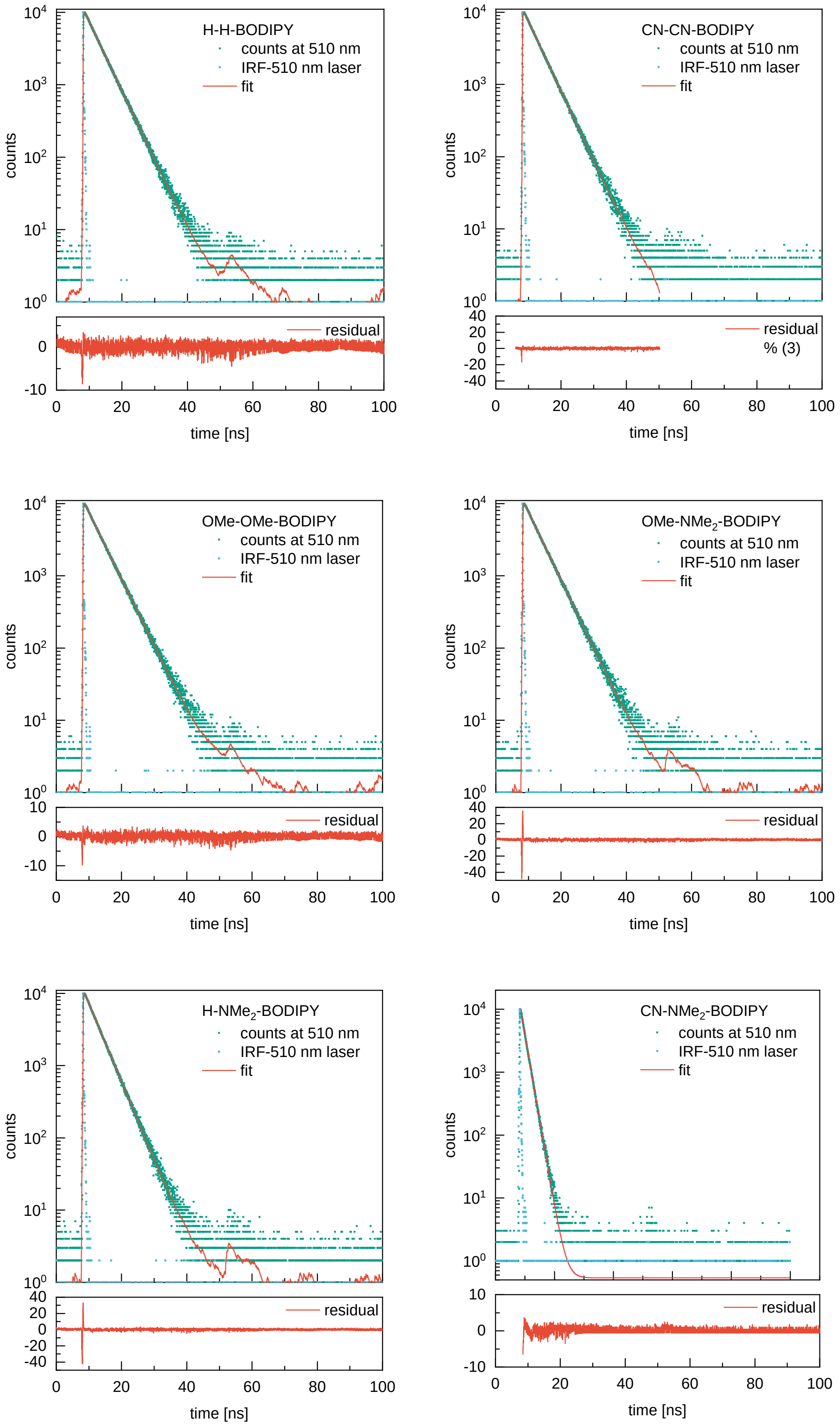

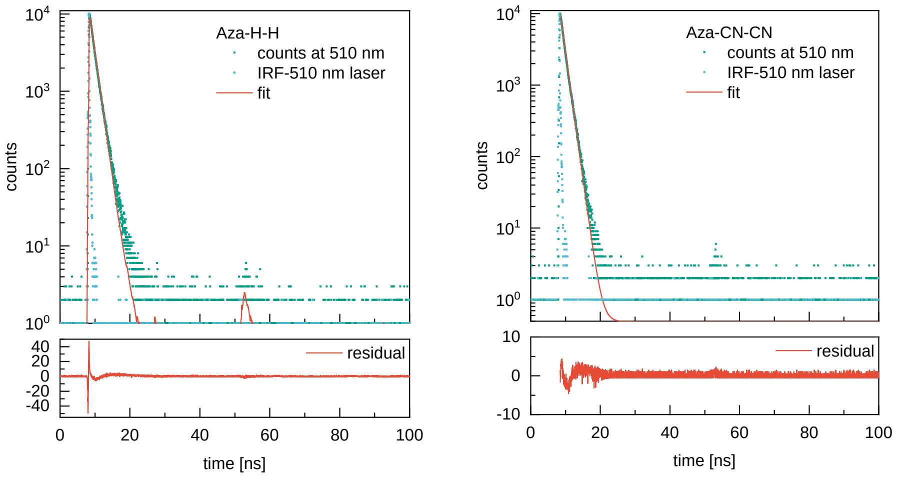

Figure S6. Emission lifetime measurements of BODIPYs and symmetrical aza-BODIPYs in green, the complementary instrument response function (IRF) in blue and the monoexponential fittings in red. The fitted lifetimes are reported in Table 1 in the main manuscript. All measurements were performed in toluene solution.

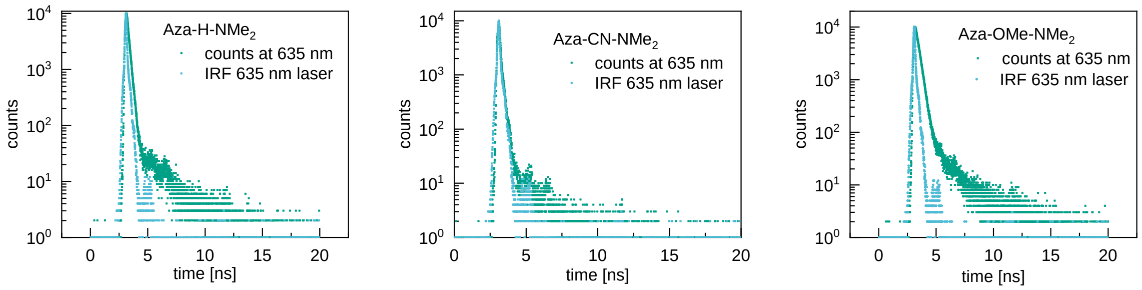

Figure S7. Emission lifetime measurements of the unsymmetrical aza-BODIPY dyes in green and the complementary IRF in blue. Experimental date could not be fitted with the reconvolution method since the IRF of the laser is too similar to the decay. Therefore, the lifetime could not be determined due to a too short-lived excited state.

Synthesis of the pyrrole boronic acid

Synthesis of the pyrrole boronic acid

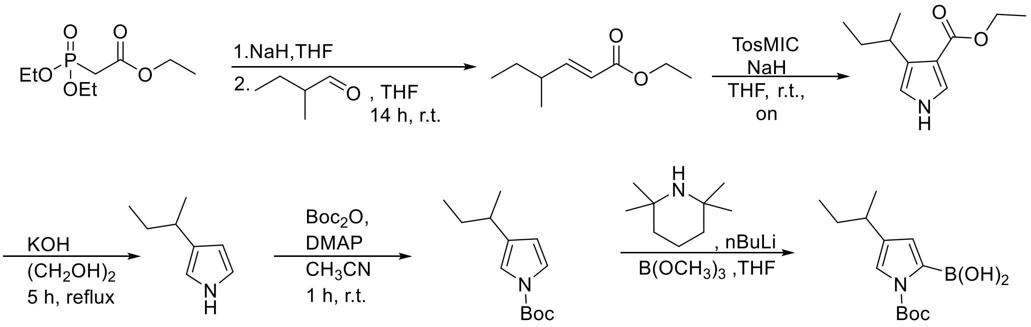

Scheme S1. Synthesis of pyrrole boronic acid.

Ethyl (*E)*-4-methylhex-2-enoate [2]

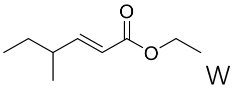

Wittig-reaction 156.23 g/mol

This reaction was performed under N_2 atmosphere. Triethyl phosphonoacetate (14.1 mL, 70 mmol, 1.4 eq) was added dropwise to a stirred solution of NaH (60% dispersion in mineral oil, 2.39 g, 60 mmol, 1.2 eq) in dry THF (15 mL) at room temperature. The resulting solution was stirred for 60 min before 2-methylbutyraldehyde (5.36 mL, 50 mmol, 1.0 eq) in THF (15 mL) was added dropwise over 1 h at room temperature. The solution was stirred for 2 h at ambient temperature. The reaction was quenched with NH_4Cl (5 mL) followed by extraction with diethyl ether (3 x 50 mL). The combined organic phases were washed with brine (2 x 50 mL) and dried over Na_2SO_4. Subsequently the solvent was removed under reduced pressure. Column chromatography on silica using 20% diethyl ether in hexane as an eluent afforded the product as a colourless oily liquid (7.41 g, 47.4 mmol, 95%). Recorded ^{1}H-NMR spectrum is in agreement with the literature. [3]

^{1}H NMR (400 MHz, $CDCl_3$) δ 6.86 (dd, *J* = 15.7, 7.8 Hz, 1H), 5.77 (dd, *J* = 15.7, 1.2 Hz, 1H), 4.18 (q, *J* = 7.1 Hz, 2H), 2.21 (m, 1H), 1.45 – 1.35 (m, 2H), 1.28 (t, *J* = 7.1 Hz, 3H), 1.04 (d, *J* = 6.7 Hz, 3H), 0.87 (t, *J* = 7.4 Hz, 3H).

Ethyl 4-(*sec*-butyl)-1H-pyrrole-3-carboxylate [4]

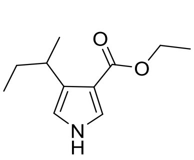

Van-Leusen-reaction 195.26 g/mol

This reaction was performed under N_2 atmosphere. A mixture of ethyl (E)-4-methylhex-2-enoate (3.90 g, 25 mmol, 1.0 eq) and TosMIC (5.06 g, 26 mmol, 1.0 eq) in dry diethyl ether/DMSO (50 mL/25 mL) was added dropwise to a stirred suspension of NaH (60% dispersion in mineral oil, 1.61 g, 42 mmol, 1.7 eq) in diethyl ether at room temperature. The resulting suspension was stirred at room temperature for 5 h. Ice water (75 mL) was added to the reaction mixture. The aqueous phase was extracted with diethyl ether (3 x 75 mL). The combined organic phases were washed with brine (1 x 100 mL), dried over Na_2SO_4 and the solvent was subsequently removed under reduced pressure. The crude product was purified using column chromatography on silica with 10 – 30% ethyl acetate/hexane. The product was afforded as an off white solid (4.52 g, 23.15 mmol, 92 %).

^{1}H NMR (400 MHz, $CDCl_3$) δ 8.23 (s, 1H), 7.39 (dd, *J* = 3.1, 2.2 Hz, 1H), 6.54 (td, *J* = 2.4, 0.8 Hz, 1H), 4.26 (qd, *J* = 7.1, 0.7 Hz, 2H), 3.28 – 3.18 (m, 1H), 1.69 (dqd, *J* = 13.3, 7.5, 5.9 Hz, 1H), 1.46 (dp, *J* = 13.3, 7.5 Hz, 1H), 1.33 (t, *J* = 7.1 Hz, 3H), 1.19 (d, *J* = 6.9 Hz, 3H), 0.90 (t, *J* = 7.4 Hz, 3H).

^{13}C {^{1}H} NMR (101 MHz, $CDCl_3$) δ 124.4, 115.1, 59.3, 31.7, 30.6, 20.7, 14.5, 12.0.

M.p.: 65-67 °C

IR: ν_{max}/cm^{-1} 3325, 2960, 2927, 2878, 1677, 1518, 1371, 1322, 1241, 1197, 1150, 1059, 785, 600.

HRMS: (ESI+) m/z calcd. For $(M+H)^+$ $C_{11}H_{18}NO_2$: 196.1338; found: 196.1345.

3-(*sec*-butyl)-1H-pyrrole [5, 6]

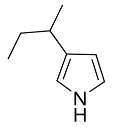

123.12 g/mol

Ethyl 4-(*sec*-butyl)-1H-pyrrole-3-carboxylate (3.90 g, 20.0 mmol, 1.0 eq) and KOH (5.62 g, 100 mmol, 5.0 eq) were put in a flask prior to evacuation and refilling with nitrogen. Ethylen glycol (40 mL) was added and the reaction mixture was heated to reflux for 5 h. After cooling down to room temperature the reaction mixture was extracted with $CHCl_3$ (3 x 50 mL). The combined organic phases were washed with brine, dried over Na_2SO_4 and the solvent was removed under reduced pressure. The product was obtained as a brown oil and used without any further purification (2.19 g, 17.8 mmol, 89 %).

^{1}H NMR (400 MHz, $CDCl_3$) δ 7.98 (s, 1H), 6.74 (td, *J* = 2.7, 2.1 Hz, 1H), 6.58 (dtt, *J* = 2.1, 1.6, 0.8 Hz, 1H), 6.13 (tdd, *J* = 2.7, 1.6, 0.4 Hz, 1H), 2.61 (h, *J* = 6.9 Hz, 1H), 1.68 – 1.44 (m, 2H), 1.22 (d, *J* = 6.9 Hz, 3H), 0.90 (t, *J* = 7.4 Hz, 3H).

^{13}C {^{1}H} NMR (101 MHz, $CDCl_3$) δ 130.4, 117.5, 113.9, 107.1, 33.4, 31.3, 21.5, 12.1.

3-(*sec*-butyl)-N-Boc-pyrrole [7, 8]

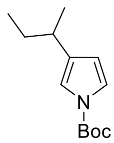

223.32 g/mol

3-(*sec*-butyl)-1H-pyrrole (45.6 mmol, 5.61 g, 1.0 eq) together with Et_3N (91.2 mmol, 12.7 mL, 2.0 eq) was put in DCM (25 mL). Boc-anhydride (55 mmol, 12.6 mL, 1.2 eq), followed by DMAP (4.56 mmol, 562 mg, 0.1 eq) was added to the solution. The reaction mixture was stirred at room temperature until full conversion (approximately 24 h). The solvent was removed under reduced pressure. The crude product was purified using column chromatography on SiO_2 (Hexane:EtOAc 1-3%). The product was isolated as an off white liquid (9.05 g, 40.5 mmol, 89%).

^{1}H NMR (400 MHz, $CDCl_3$) δ 7.18 – 7.14 (m, 1H), 6.97 (t, *J* = 2.0 Hz, 1H), 6.10 (dd, *J* = 3.3, 1.7 Hz, 1H), 2.51 (h, *J* = 6.9 Hz, 1H), 1.58 (s, 9H), 1.57 – 1.47 (m, 2H), 1.17 (d, *J* = 6.9 Hz, 3H), 0.87 (t, *J* = 7.4 Hz, 3H).

^{13}C {^{1}H} NMR (101 MHz, $CDCl_3$) δ 149.0, 133.6, 119.9, 115.6, 111.5, 83.1, 33.4, 30.5, 28.0, 20.9, 11.9.

IR: ν_{max}/cm^{-1} 2963, 2930, 1740, 1559, 1488, 1457, 1370, 1351, 1153, 972, 772.

GC/MS: m/z calcd. for $C_{13}H_{21}NO_2$ (M^+, 100 %): 223.16; found: 223.2.

(1-(tert-butoxycarbonyl)-4-(sec-butyl)-1H-pyrrol-2-yl)boronic acid [9]

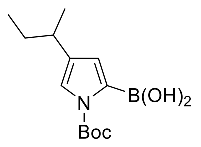

267.13 g/mol

This reaction was performed under N_2 atmosphere. A solution of 2,2,6,6-tetramethylpiperidin (13.7 mmol, 2.3 mL, 1.2 eq) in dry THF (40 mL) was cooled to -78 ˚C. To the cooled solution, n-BuLi (2.5 M in hexanes) (14.8 mmol, 5.9 mL, 1.3 eq) was added dropwise over 30 min. The mixture was warmed to 0 ˚C. After stirring at this temperature for 30 min the mixture was cooled down to -78 ˚C again. 3-(*sec*-butyl)-N-Boc-pyrrole (11.4 mmol, 2.54 g, 1.0 eq) in dry THF (20 mL) was added dropwise over 30 min and the reaction was left to stir at -78 ˚C for 2 h. Trimethylborate (57.0 mmol, 6.4 mL, 5.0 eq) was added to the reaction mixture over 30 min and the resulting solution was left to warm up to room temperature where it was left to stir over night. The reaction mixture was afterwards diluted with EtOAc (100 mL). The organic phase was washed with H_2O (1 x 100 mL) and brine (2 x 50 mL) and subsequently dried over Na_2SO_4 and the solvent was removed under reduced pressure. The crude product was recrystallized in hexane giving the product as a white solid (1.42 g, 5.32 mmol, 47 %).

^{1}H NMR (400 MHz, DMSO-*d*$_6$) δ 7.98 (s, 2H), 7.02 (dd, J = 1.7, 0.8 Hz, 1H), 6.33 (d, J = 1.7 Hz, 1H), 2.47 – 2.38 (m, 1H), 1.49 (s, 9H), 1.49 – 1.32 (m, 2H), 1.07 (d, J = 6.9 Hz, 3H), 0.77 (t, J = 7.4 Hz, 3H).

^{13}C {^{1}H} NMR (101 MHz, DMSO-*d*$_6$) δ 150.4, 133.0, 120.7, 119.1, 84.1, 32.9, 30.4, 27.9, 21.4, 12.3.

IR: ν_{max}/cm^{-1} 3356, 2961, 2926, 1715, 1371, 1330, 1283, 1251, 1159, 774.

M.p.: 118–120 °C

Synthesis of the pyrrole derivatives

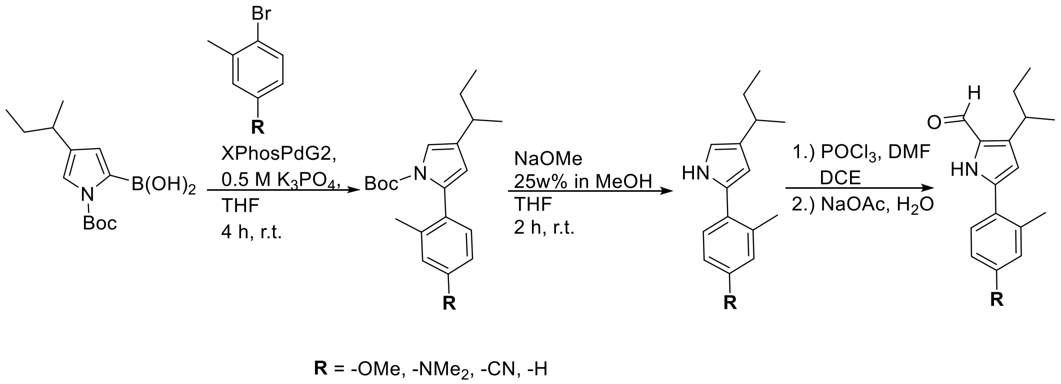

Scheme S2. Synthesis to the Pyrrole boronic acid, followed by Suzuki coupling between pyrrole boronic acid and arylbromide, followed by Boc-deprotection and then a Vilsmeier-Haack type formylation, which is performed in case of synthesis of BODIPY derivatives.

References

1. Frisch, M. J.; Trucks, G. W.; Schlegel, H. B.; Scuseria, G. E.; Robb, M. A.; Cheeseman, J. R.; Scalmani, G.; Barone, V.; Petersson, G. A.; Nakatsuji, H.; Li, X.; Caricato, M.; Marenich, A. V.; Bloino, J.; Janesko, B. G.; Gomperts, R.; Mennucci, B.; Hratchian, H. P.; Ortiz, J. V.; Izmaylov, A. F.; Sonnenberg, J. L.; Williams; Ding, F.; Lipparini, F.; Egidi, F.; Goings, J.; Peng, B.; Petrone, A.; Henderson, T.; Ranasinghe, D.; Zakrzewski, V. G.; Gao, J.; Rega, N.; Zheng, G.; Liang, W.; Hada, M.; Ehara, M.; Toyota, K.; Fukuda, R.; Hasegawa, J.; Ishida, M.; Nakajima, T.; Honda, Y.; Kitao, O.; Nakai, H.; Vreven, T.; Throssell, K.; Montgomery Jr., J. A.; Peralta, J. E.; Ogliaro, F.; Bearpark, M. J.; Heyd, J. J.; Brothers, E. N.; Kudin, K. N.; Staroverov, V. N.; Keith, T. A.; Kobayashi, R.; Normand, J.; Raghavachari, K.; Rendell, A. P.; Burant, J. C.; Iyengar, S. S.; Tomasi, J.; Cossi, M.; Millam, J. M.; Klene, M.; Adamo, C.; Cammi, R.; Ochterski, J. W.; Martin, R. L.; Morokuma, K.; Farkas, O.; Foresman, J. B.; Fox, D. J. *Gaussian 16 Rev. C.01*, Wallingford, CT, 2016.
2. Banerjee, S.; Nayek, A.; Sinha, S.; Bhaumik, T.; Ghosh, S., Alkoxy group facilitated ring closing metathesis (RCM) of acyclic 1,6-dienes: Facile synthesis of non-racemic highly substituted cyclopentenols. *J. Mol. Catal. A: Chem.* **2006,** *254* (1-2), 85-92.
3. Castelani, P.; Comasseto, J. V., Diastereoselective synthesis of alpha,beta-unsaturated systems. *Tetrahedron* **2005,** *61* (9), 2319-2326.
4. Jin, Y. Z.; Fu, D. X.; Ma, N.; Li, Z. C.; Liu, Q. H.; Xiao, L.; Zhang, R. H., Synthesis and Biological Evaluation of 3-Substituted-indolin-2-one Derivatives Containing Chloropyrrole Moieties. *Molecules* **2011,** *16* (11), 9368-9385.
5. Liu, D.; Lash, T. D., Conjugated Macrocycles Related to the Porphyrins. 25. Proton NMR Spectroscopic Evidence for a Preferred [18]Annulene Substructure in Carbaporphyrins from the Magnitude of Selected 4JH,H CHCCH3 Coupling Constants. *J. Org. Chem.* **2003,** *68* (5), 1755-1761.
6. Maity, A.; Ghosh, U.; Giri, D.; Mukherjee, D.; Maiti, T. K.; Patra, S. K., A water-soluble BODIPY based 'OFF/ON' fluorescent probe for the detection of Cd2+ ions with high selectivity and sensitivity. *Dalton Transactions* **2019,** *48* (6), 2108-2117.
7. Grehn, L.; Ragnarsson, U., A Convenient Method for the Preparation of 1-(tert-Butyloxycarbonyl) pyrroles. *Angewandte Chemie International Edition in English* **1984,** *23* (4), 296-301.
8. Fraile, J. M.; Le Jeune, K.; Mayoral, J. A.; Ravasio, N.; Zaccheria, F., CuO/SiO2 as a simple, effective and recoverable catalyst for alkylation of indole derivatives with diazo compounds. *Organic & Biomolecular Chemistry* **2013,** *11* (26), 4327-4332.
9. Kancharla, P.; Kelly, J. X.; Reynolds, K. A., Synthesis and Structure–Activity Relationships of Tambjamines and B-Ring Functionalized Prodiginines as Potent Antimalarials. *J. Med. Chem.* **2015,** *58* (18), 7286-7309.

Appendix : ^{1}H and $^{13}C\{^{1}H\}$ spectra of novel compounds

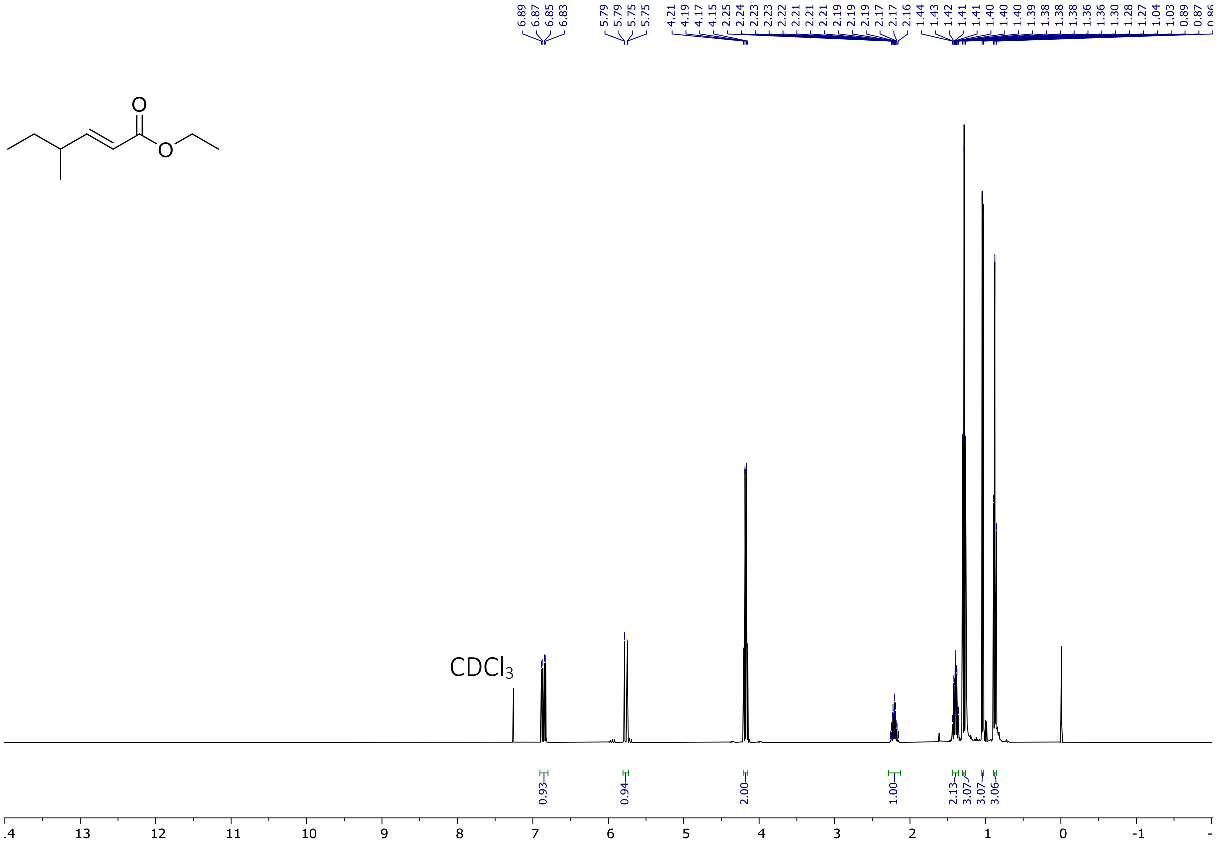

Figure S8. ^{1}H NMR spectrum (400 MHz, $CDCl_3$) of Ethyl (*E*)-4-methylhex-2-enoate.

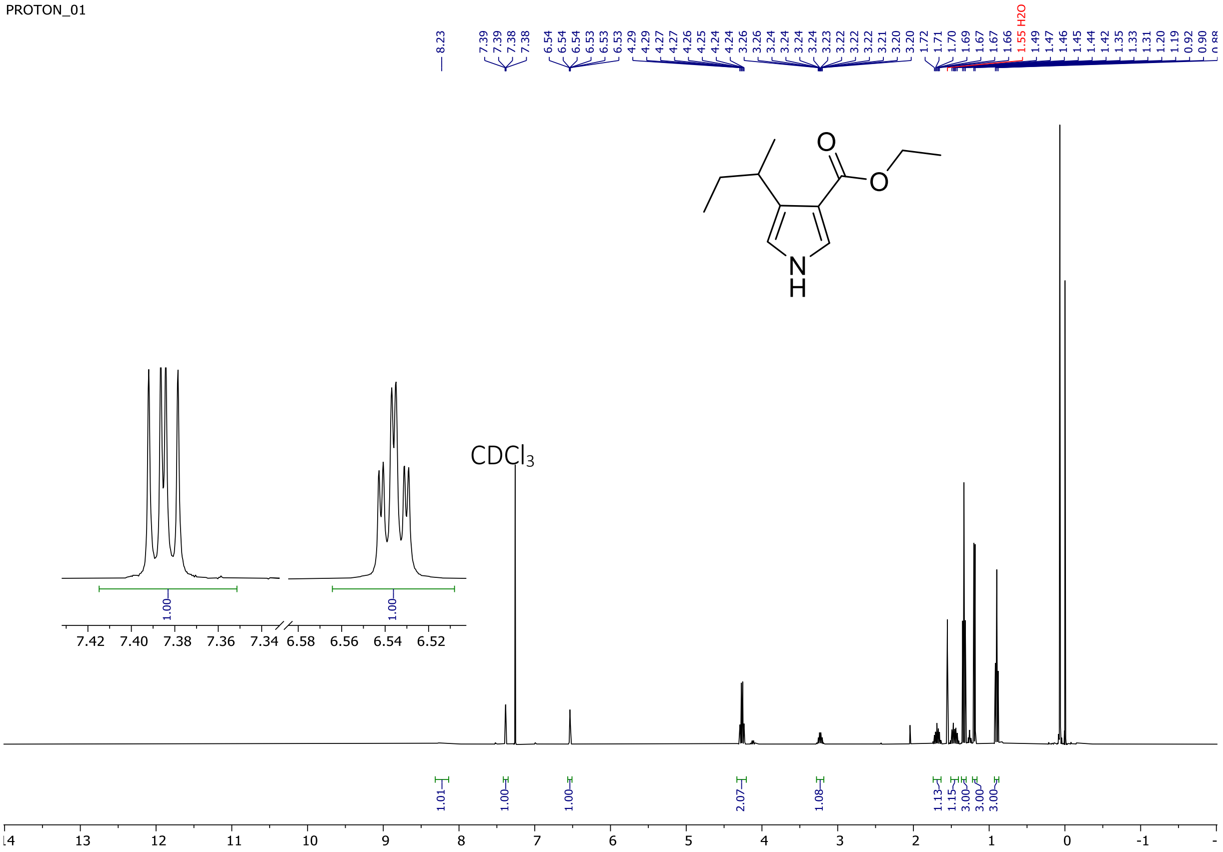

Figure S9. ^{1}H NMR spectrum (400 MHz, $CDCl_3$) of Ethyl 4-(*sec*-butyl)-1H-pyrrole-3-carboxylate.

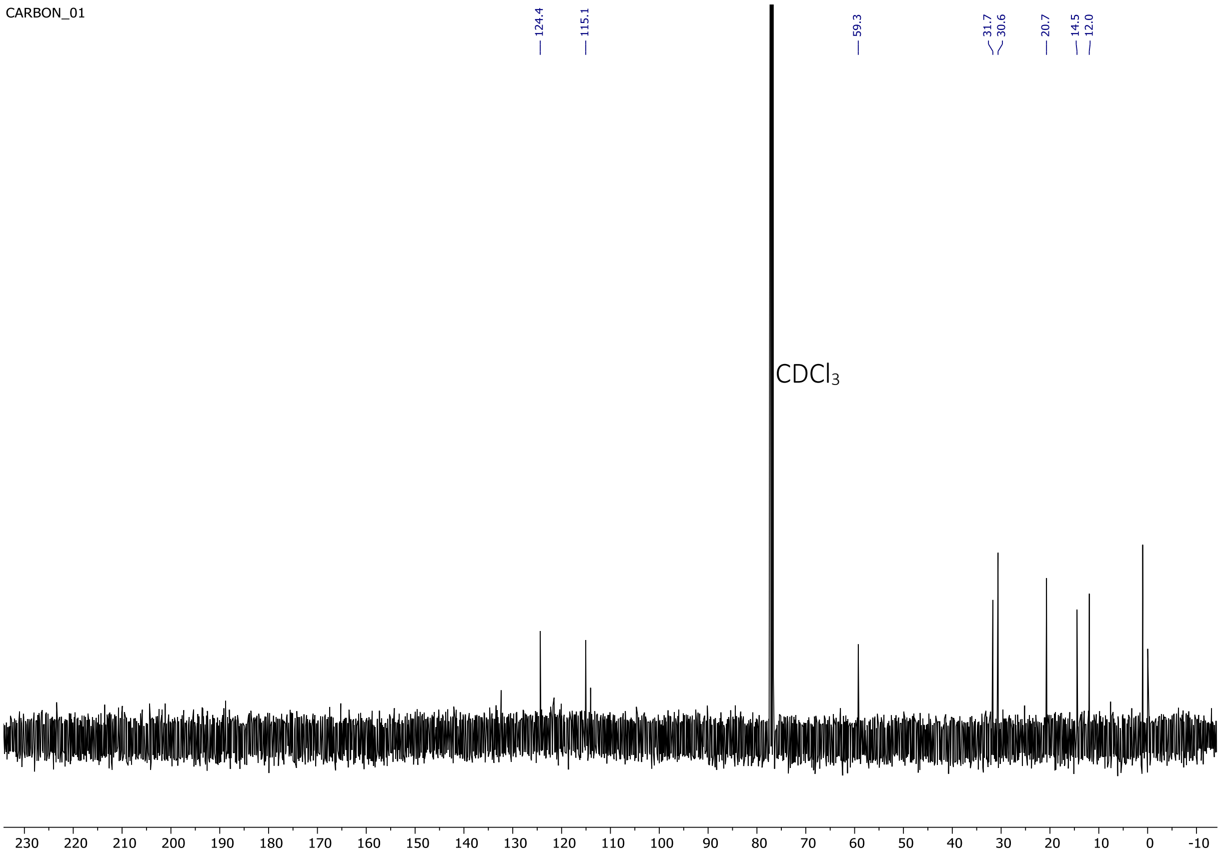

Figure S10. $^{13}C\ \{^{1}H\}$ NMR spectrum (101 MHz, $CDCl_3$) of Ethyl 4-(*sec*-butyl)-1H-pyrrole-3-carboxylate.

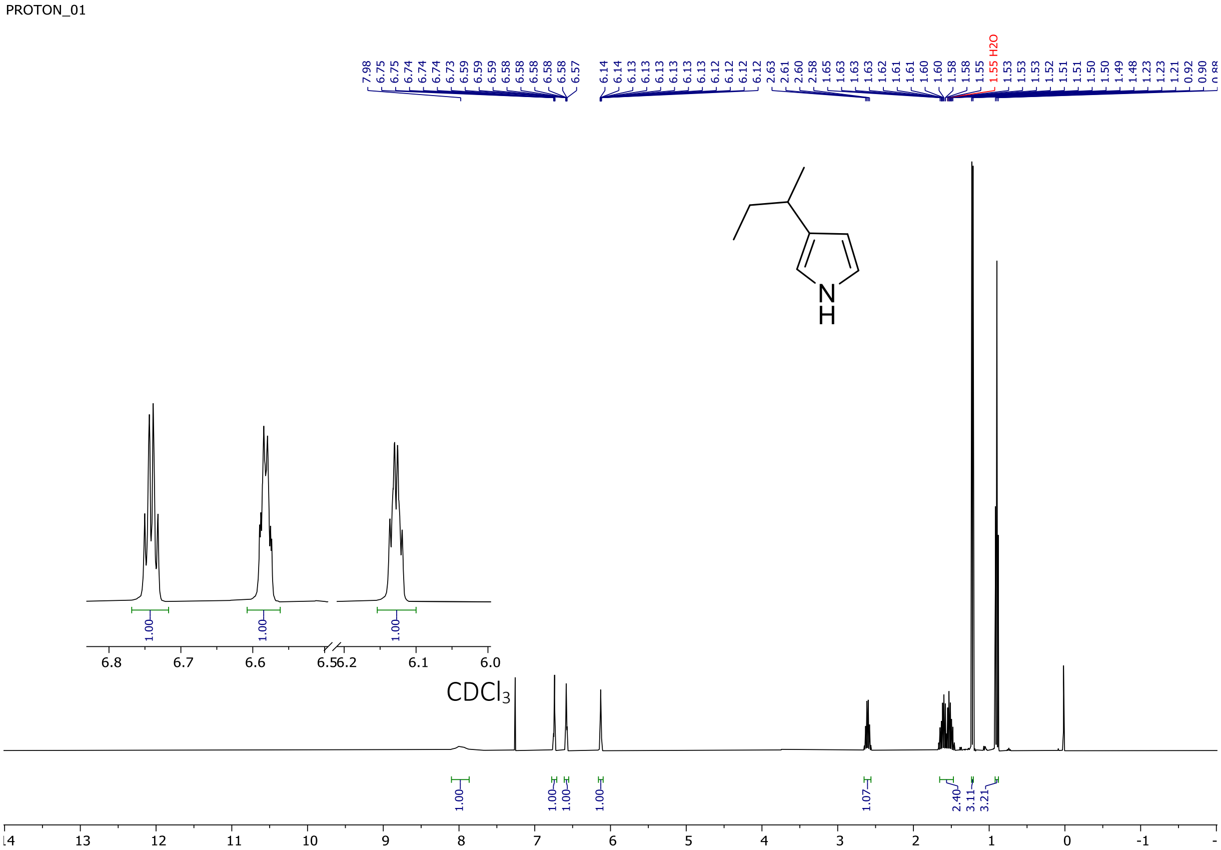

Figure S11. ^{1}H NMR spectrum (400 MHz, $CDCl_3$) of 3-(*sec*-butyl)-1H-pyrrole.

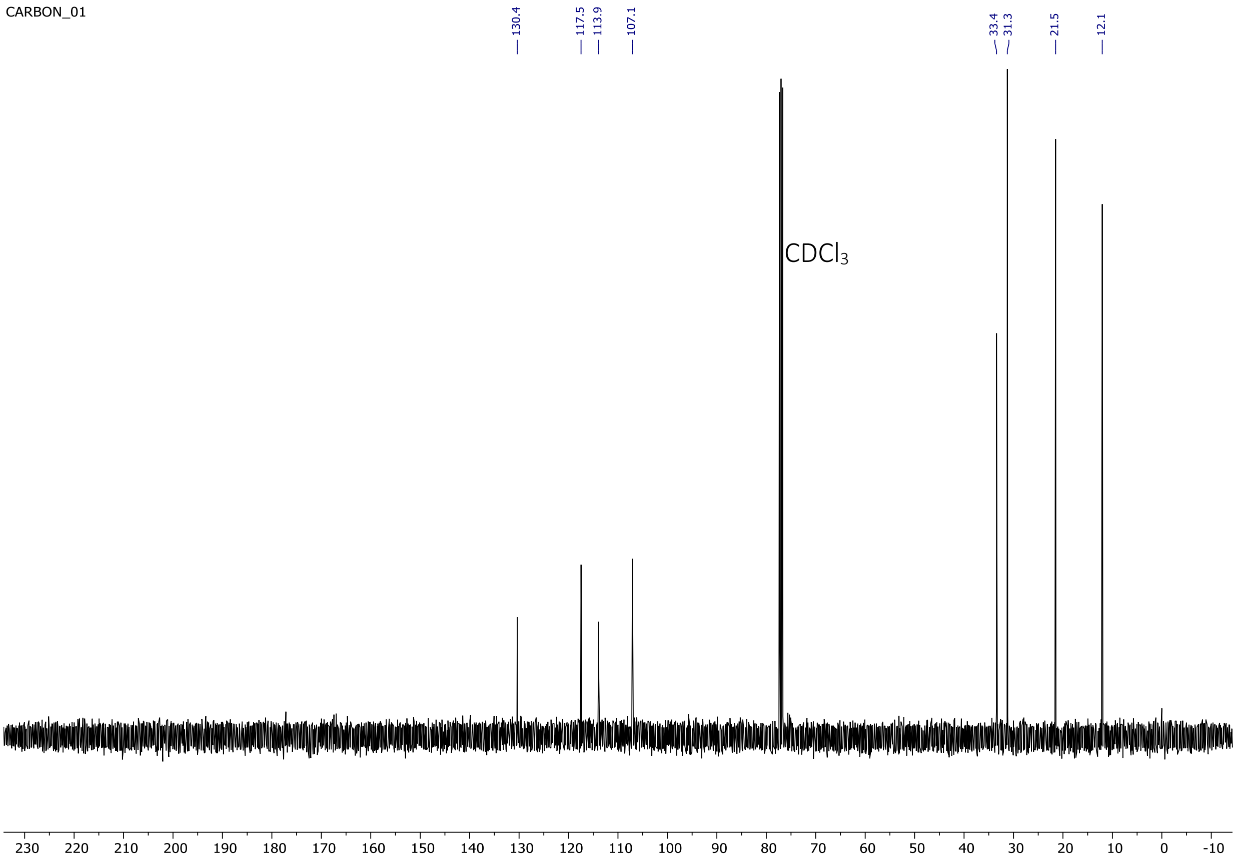

Figure S12. ^{13}C {^{1}H} NMR spectrum (101 MHz, $CDCl_3$) of 3-(*sec*-butyl)-1H-pyrrole.

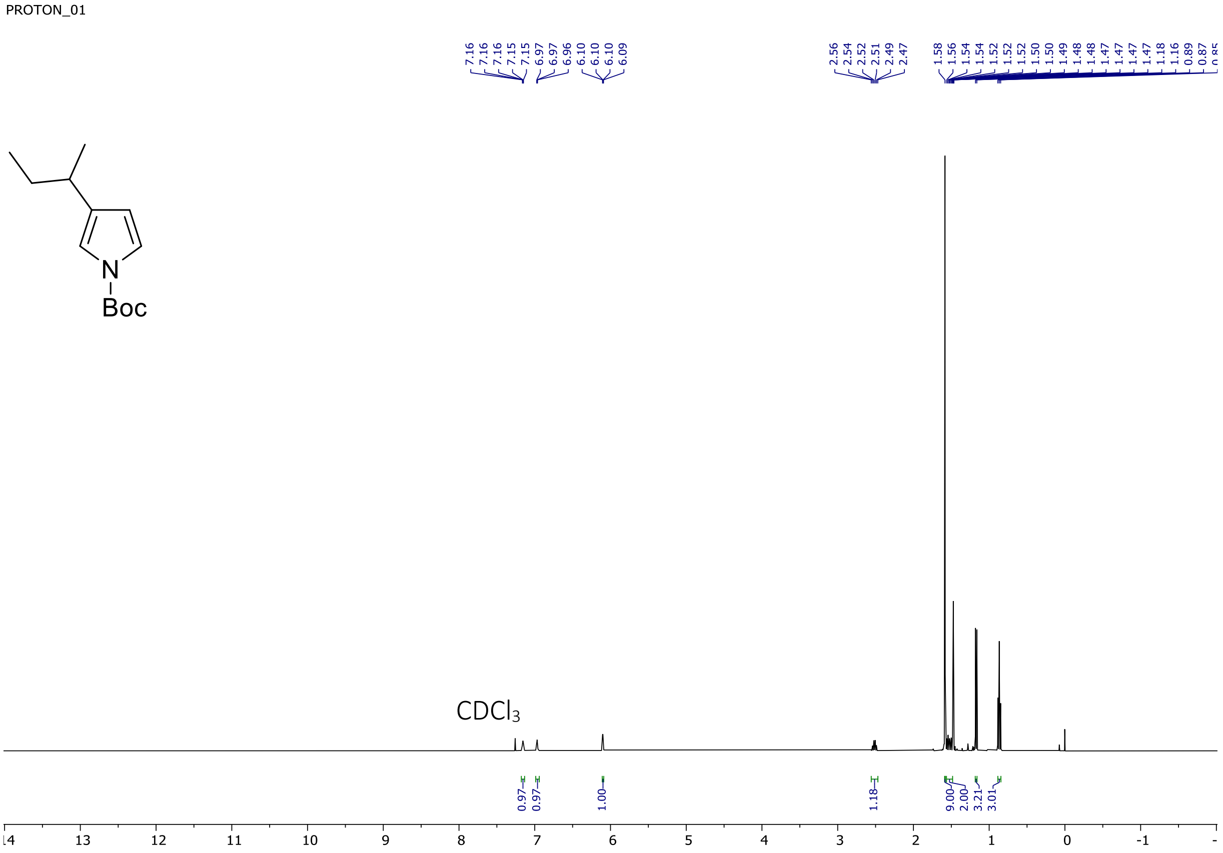

Figure S13 ^{1}H NMR spectrum (400 MHz, $CDCl_3$) of 3-(sec-butyl)-N-Boc-pyrrole.

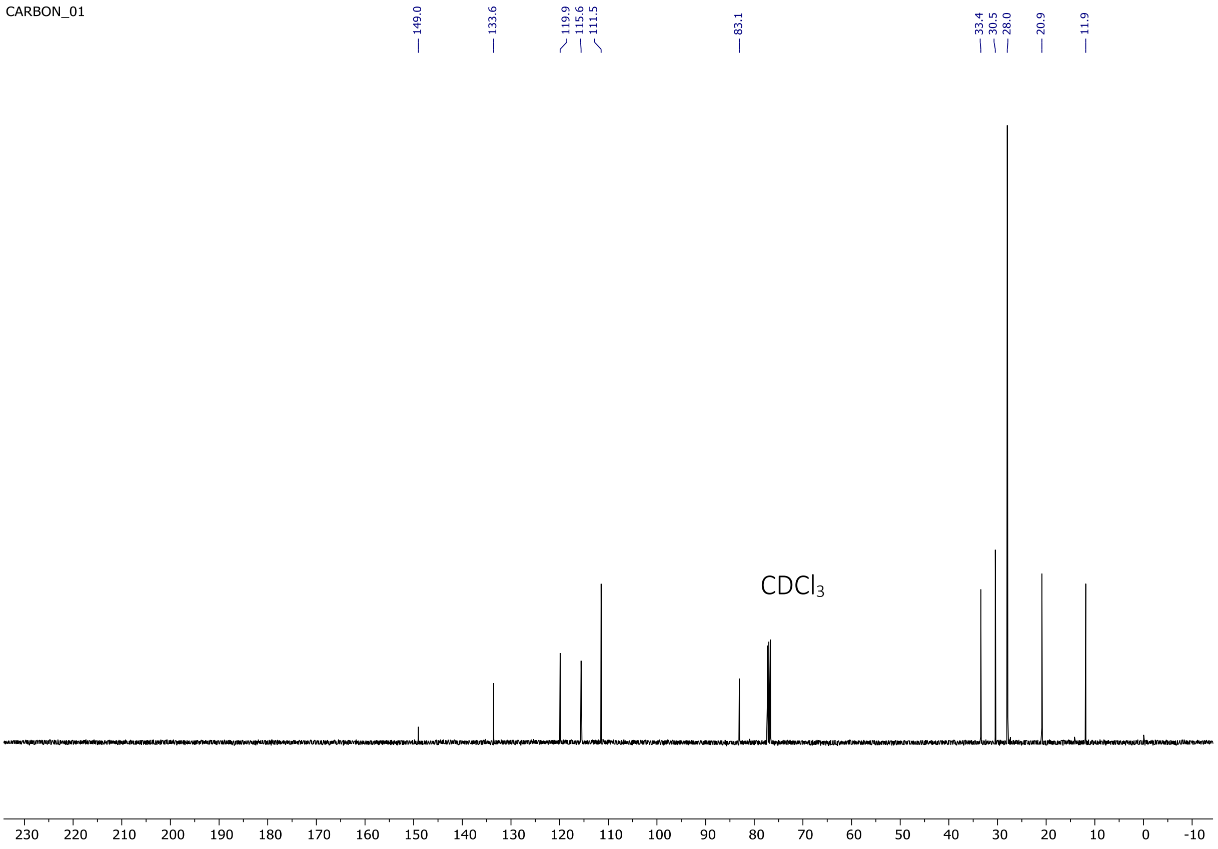

Figure S14. ^{13}C {^{1}H} NMR spectrum (101 MHz, $CDCl_3$) of 3-(sec-butyl)-N-Boc-pyrrole.

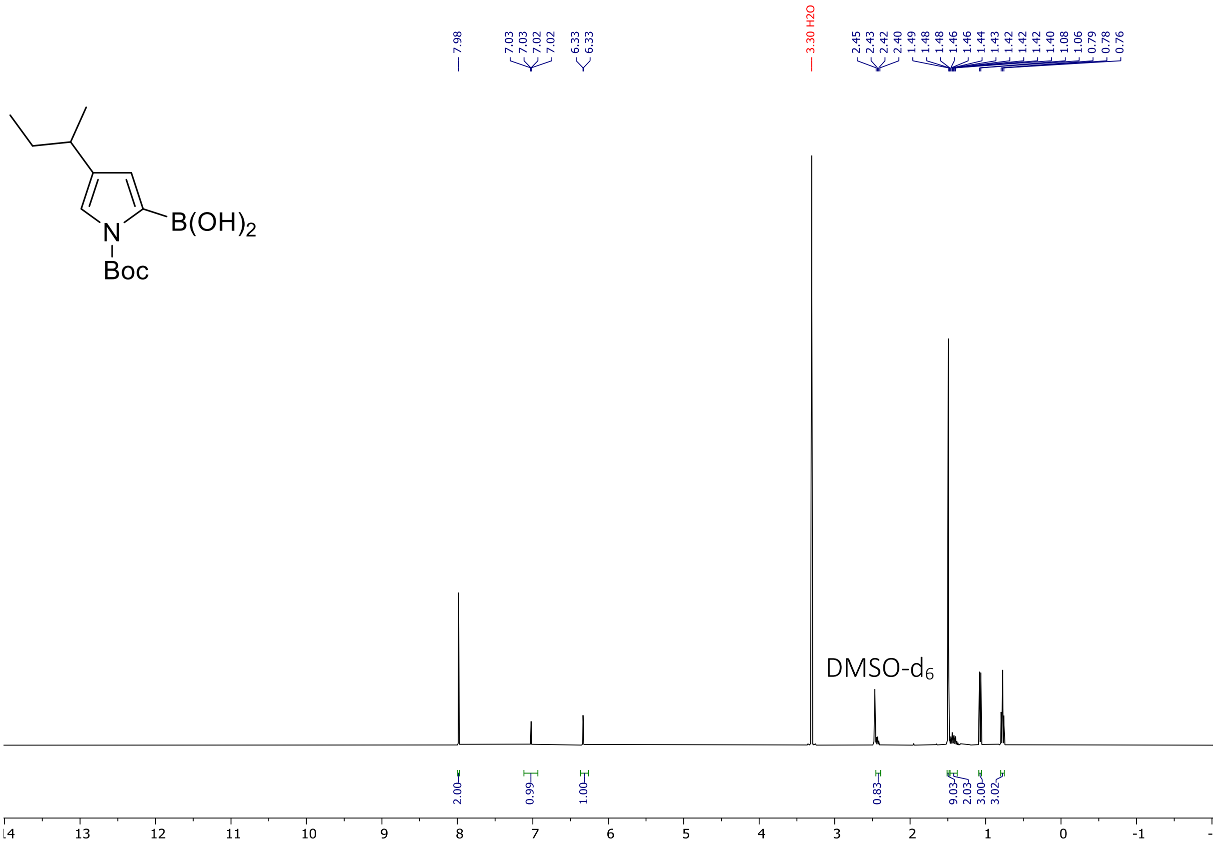

Figure S15. ^{1}H NMR spectrum (400 MHz, DMSO-d_6) of (1-(tert-butoxycarbonyl)-4-(sec-butyl)-1H-pyrrol-2-yl)boronic acid.

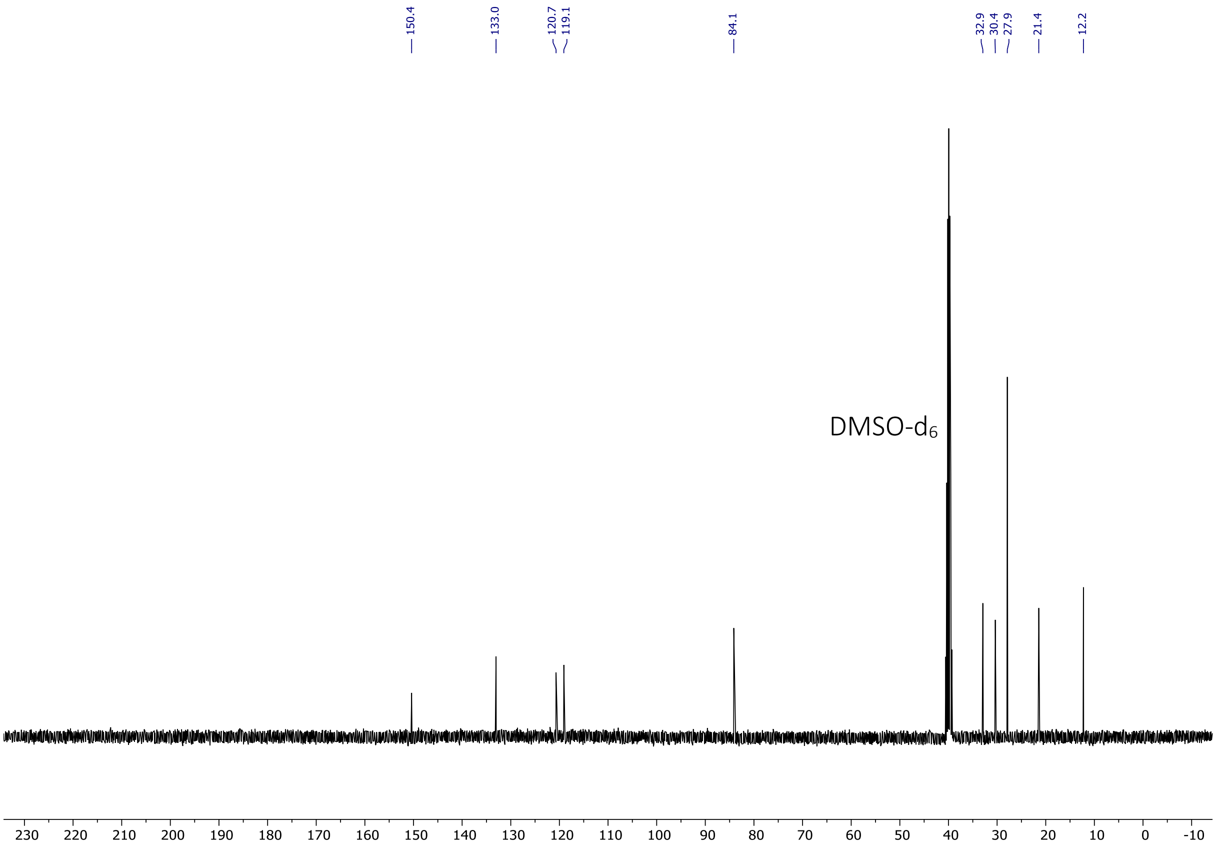

Figure S16. ^{13}C {^{1}H} NMR spectrum (101 MHz, DMSO-d_6) of (1-(tert-butoxycarbonyl)-4-(sec-butyl)-1H-pyrrol-2-yl)boronic acid.

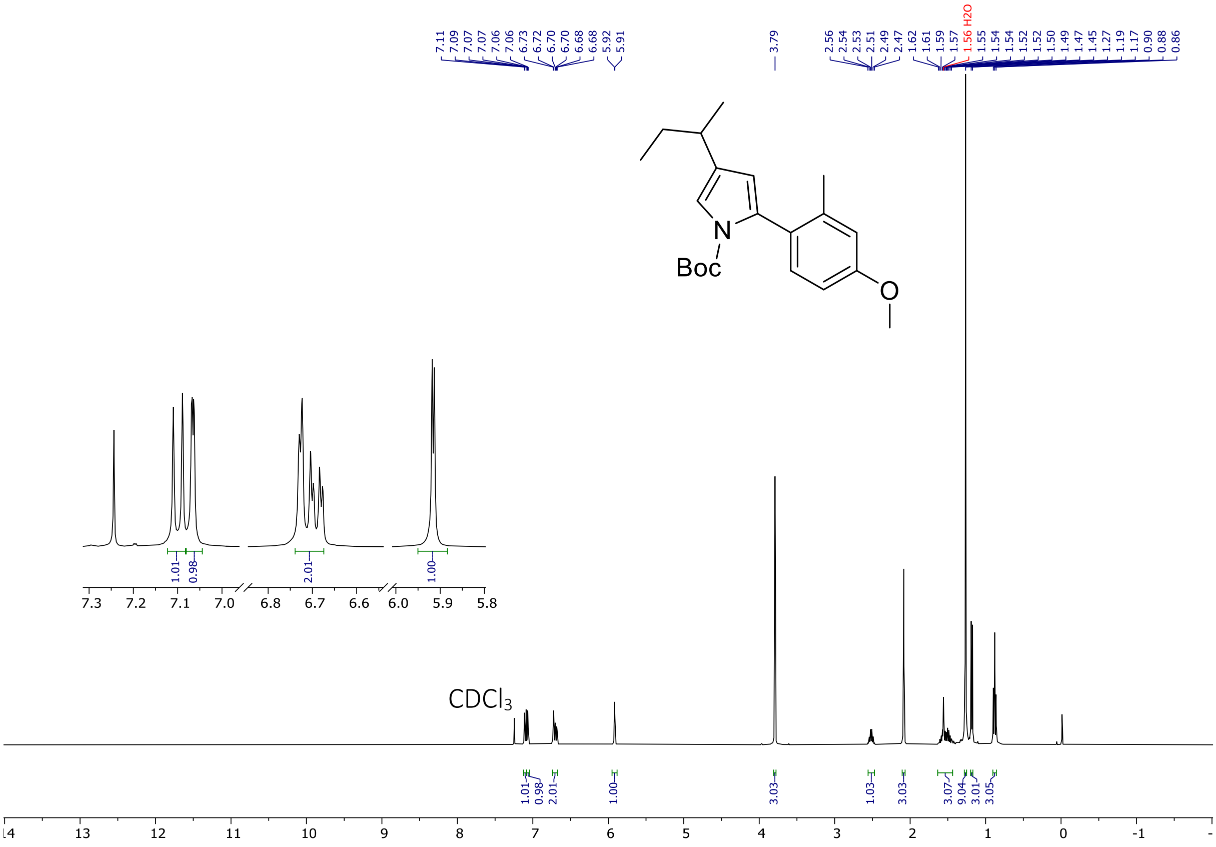

Figure S17. ^{1}H NMR spectrum (400 MHz, $CDCl_3$) of OMe-SCP-NBoc.

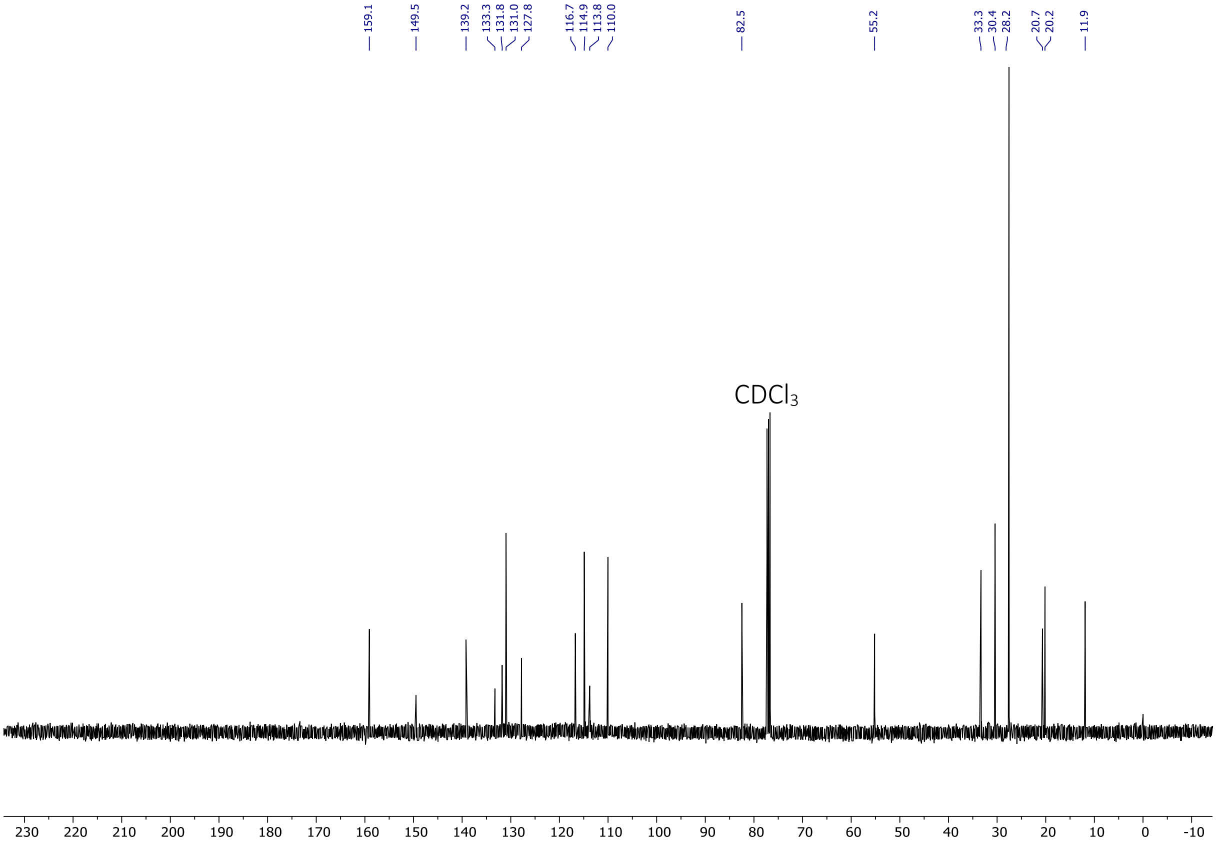

Figure S18. $^{13}C\ \{^{1}H\}$ NMR spectrum (101 MHz, $CDCl_3$) of OMe-SCP-NBoc.

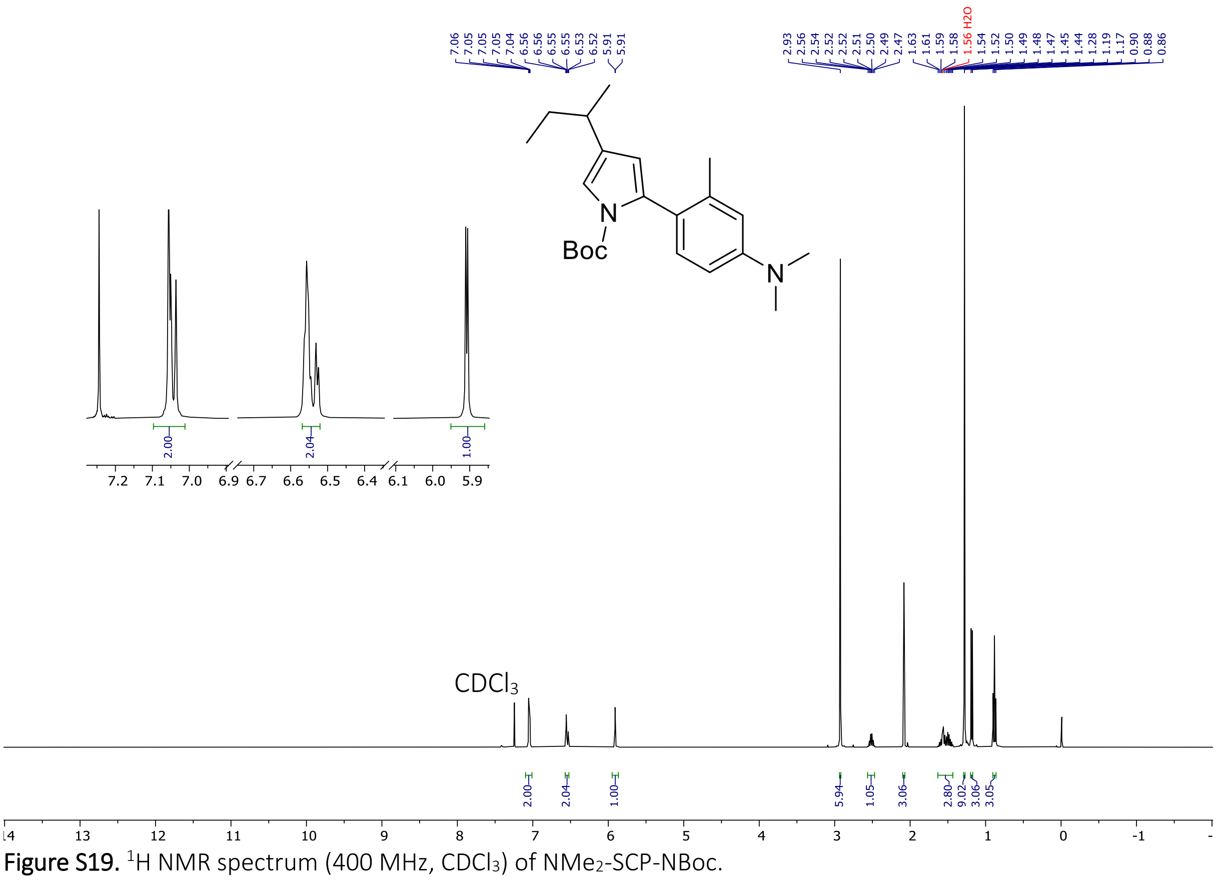

Figure S19. ^{1}H NMR spectrum (400 MHz, $CDCl_3$) of NMe_2-SCP-NBoc.

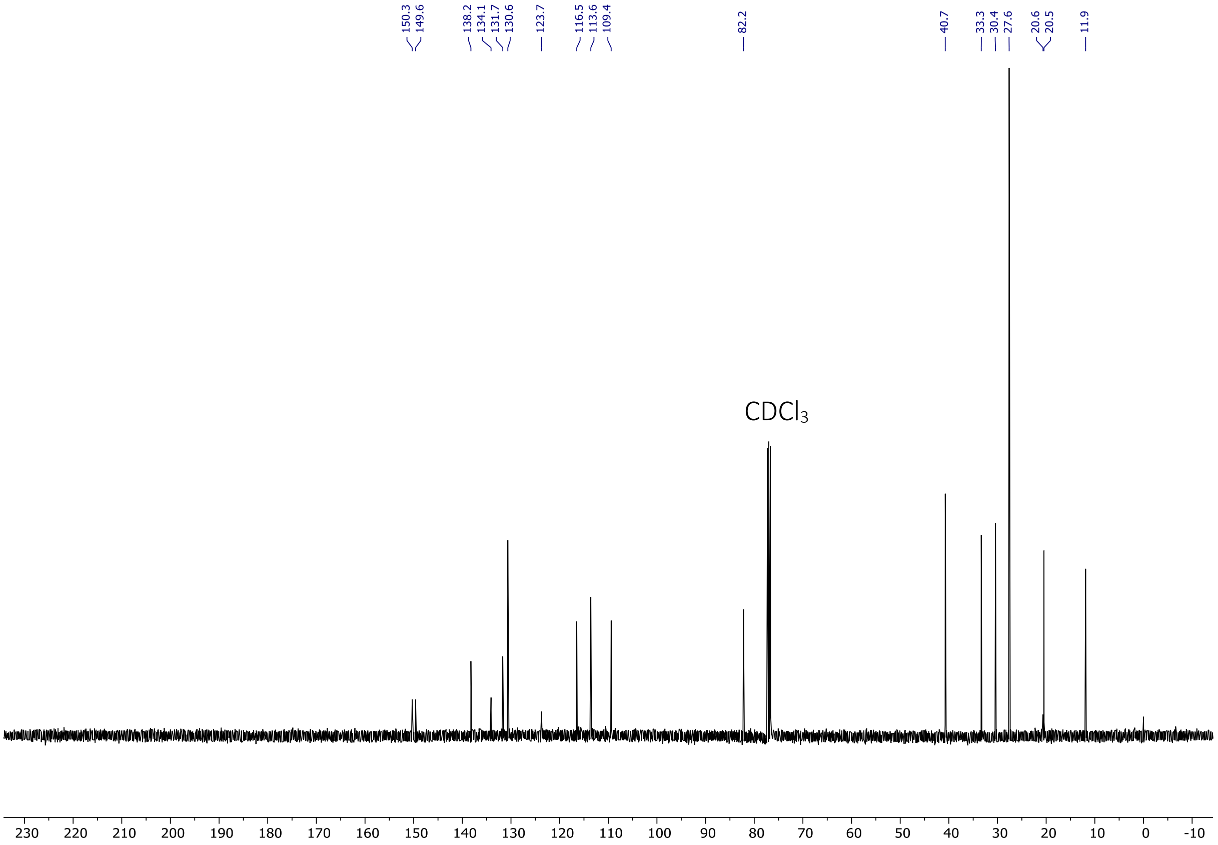

Figure S20. $^{13}C\{^1H\}$ NMR spectrum (101 MHz, $CDCl_3$) of NMe_2-SCP-NBoc.

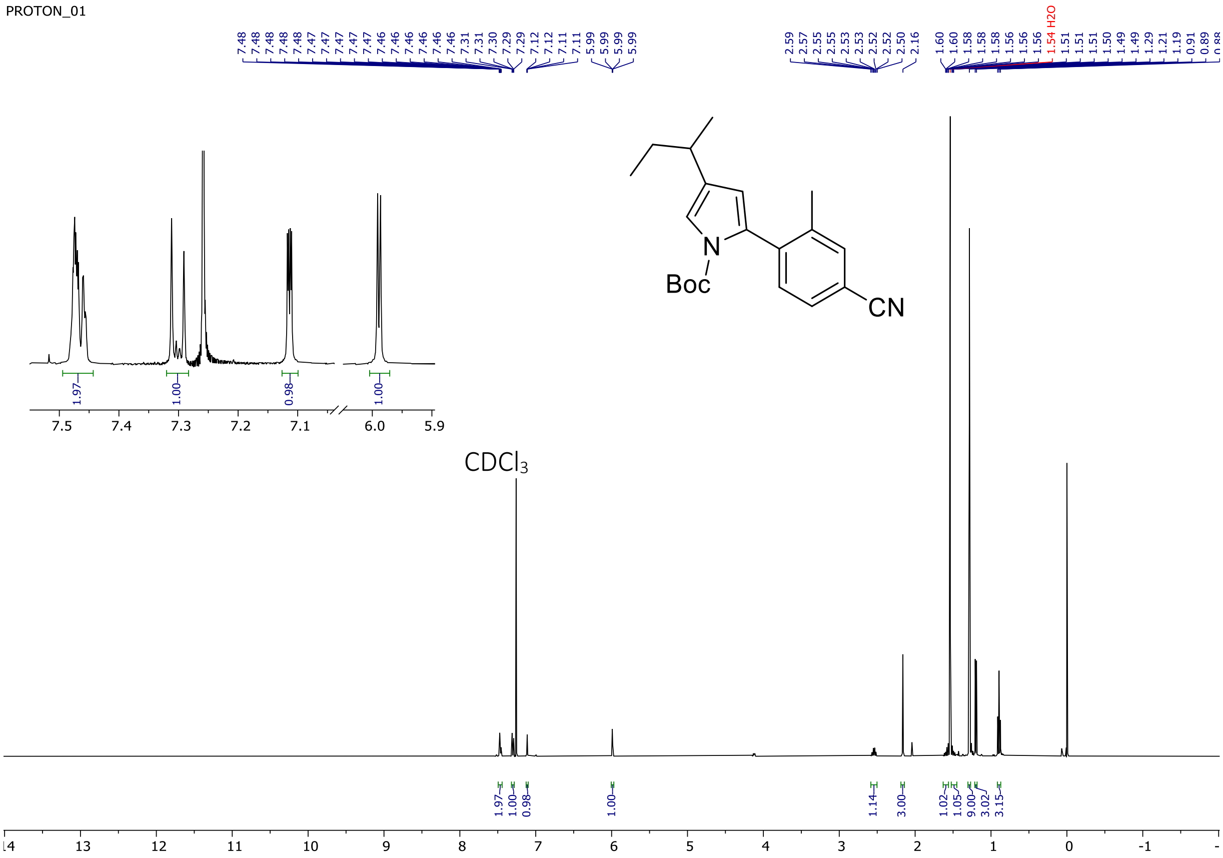

Figure S21. ^1H NMR spectrum (400 MHz, $CDCl_3$) of CN-SCP-NBoc.

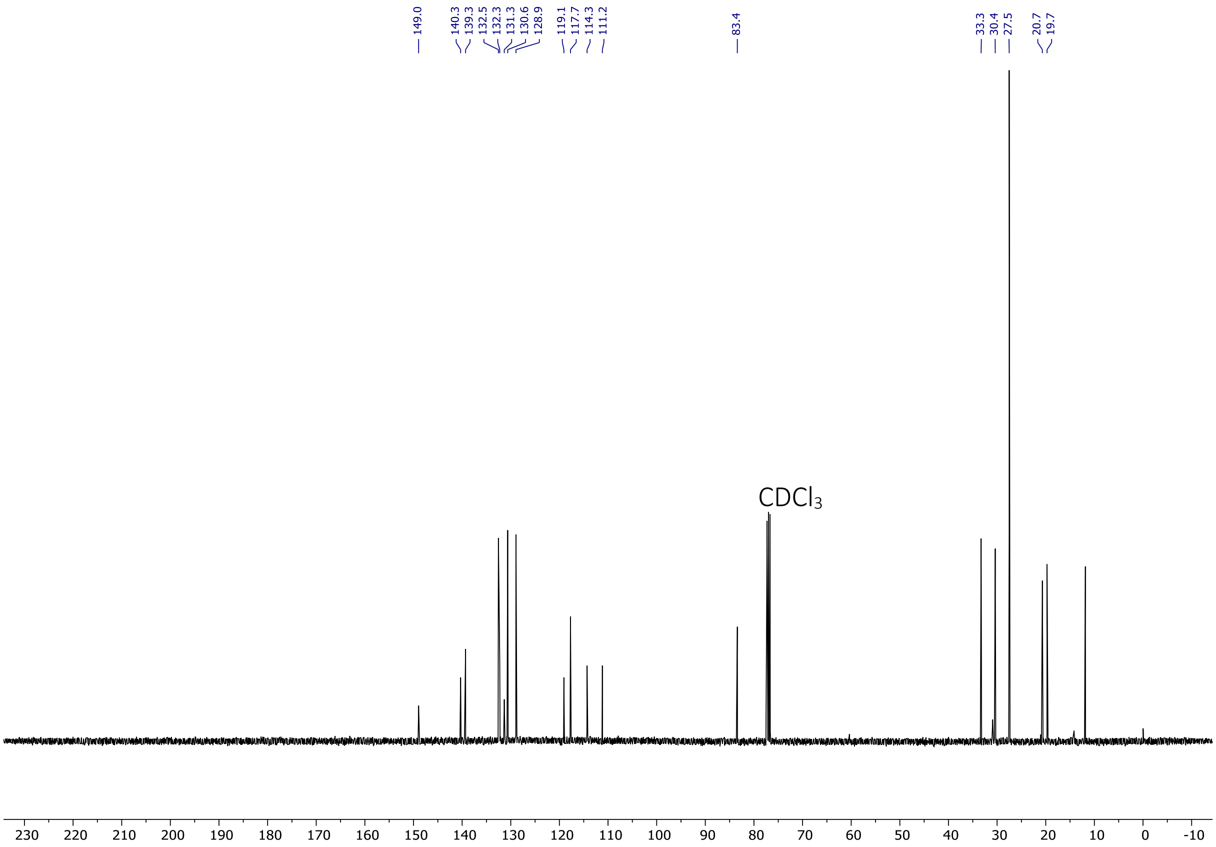

Figure S22. $^{13}C\{^{1}H\}$ NMR spectrum (101 MHz, $CDCl_3$) of CN-SCP-NBoc.

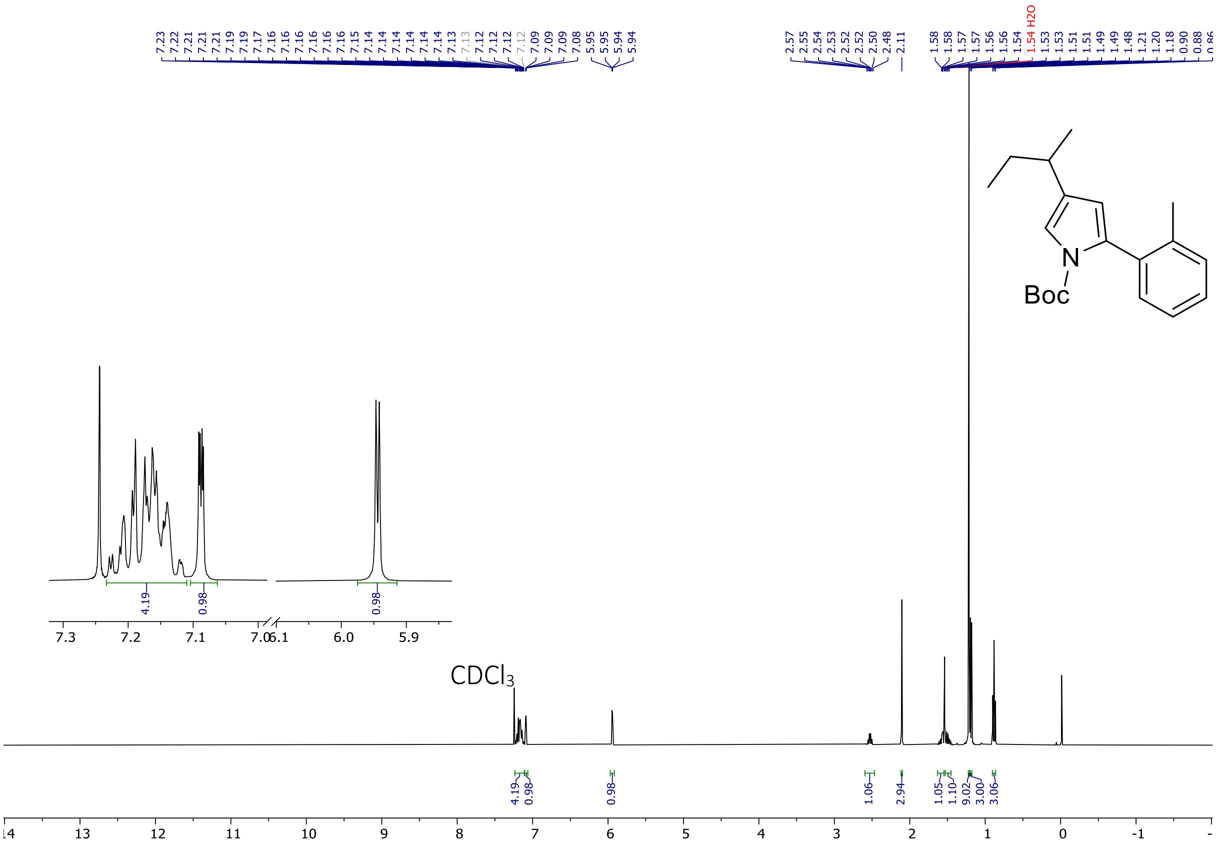

Figure S23. ^{1}H NMR spectrum (400 MHz, $CDCl_3$) of H-SCP-NBoc.

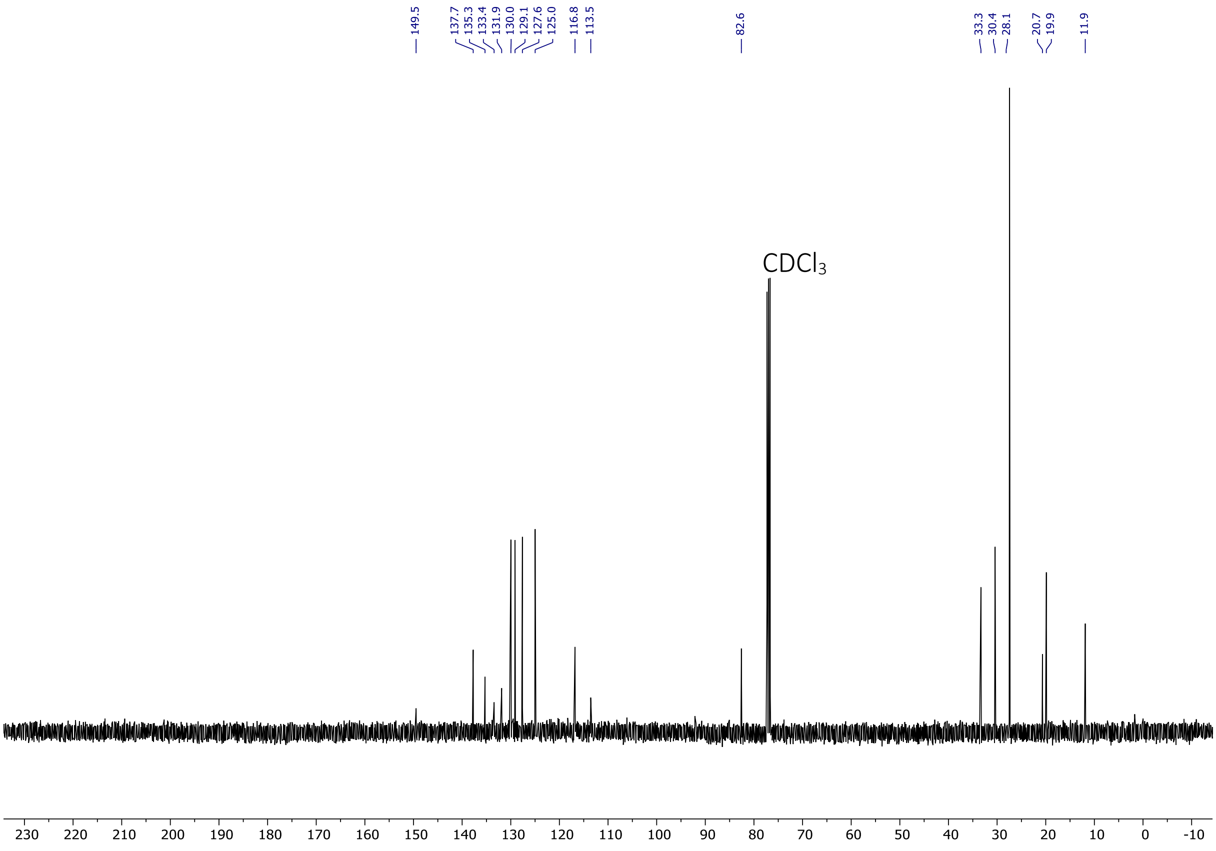

Figure S24. ^{13}C {^{1}H} NMR spectrum (101 MHz, $CDCl_3$) of H-SCP-NBoc.

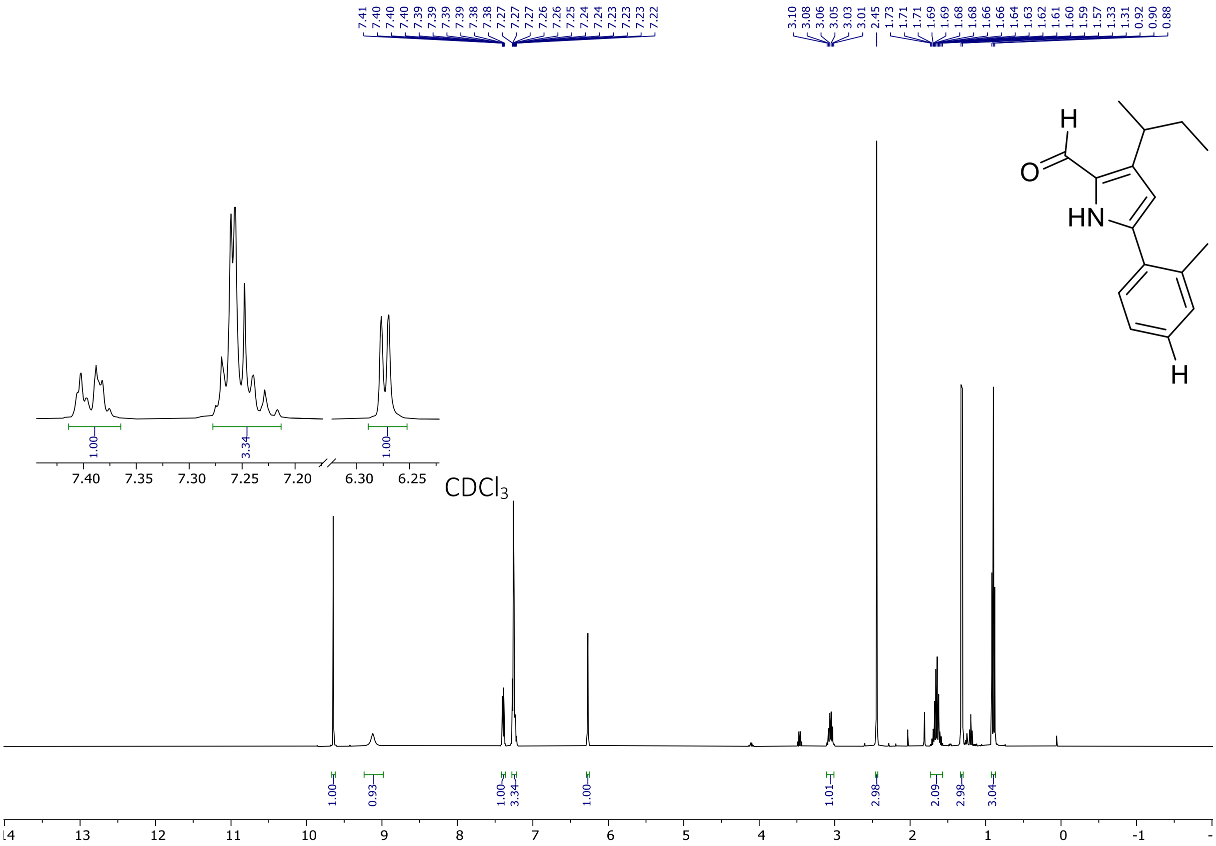

Figure S25. ^{1}H NMR spectrum (400 MHz, $CDCl_3$) of H-SCP-NH-αCOH.

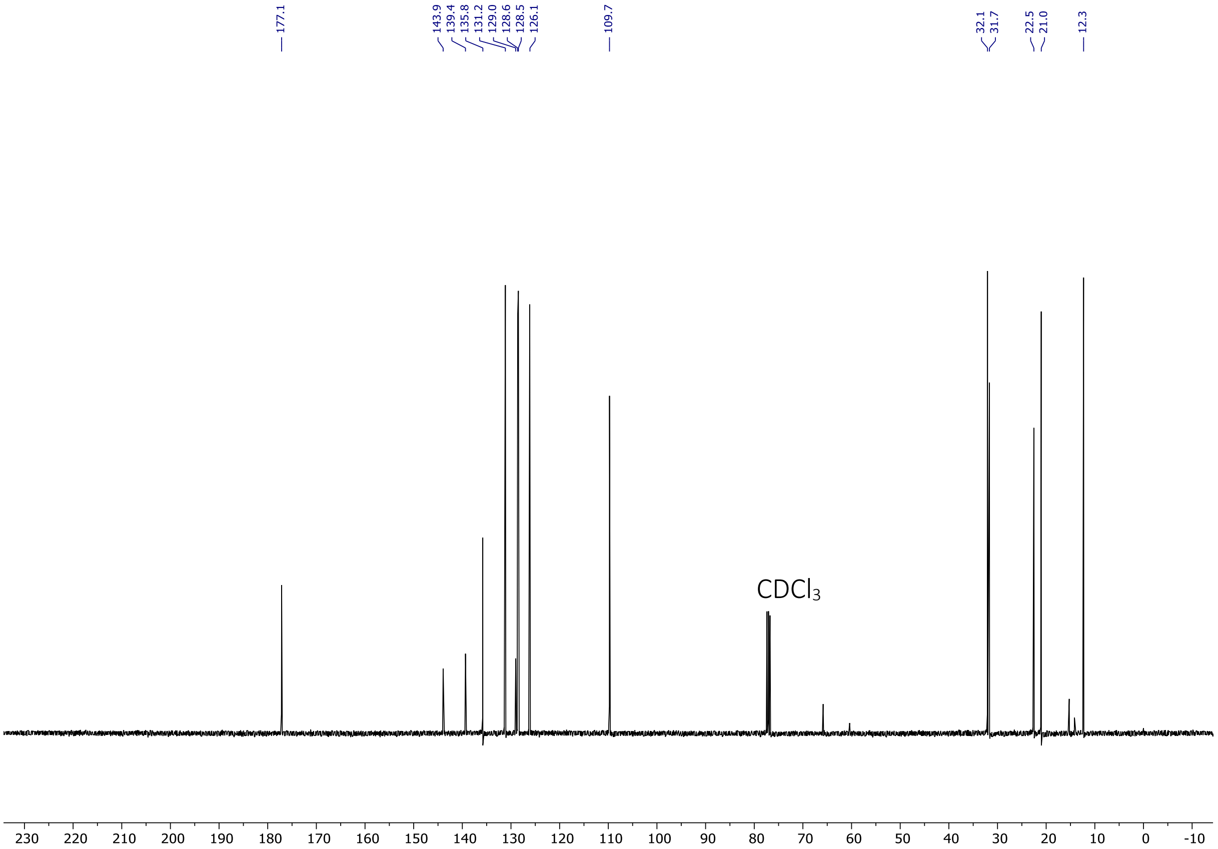

Figure S26. $^{13}C\ \{^{1}H\}$ NMR spectrum (101 MHz, $CDCl_3$) of H-SCP-NH-αCOH.

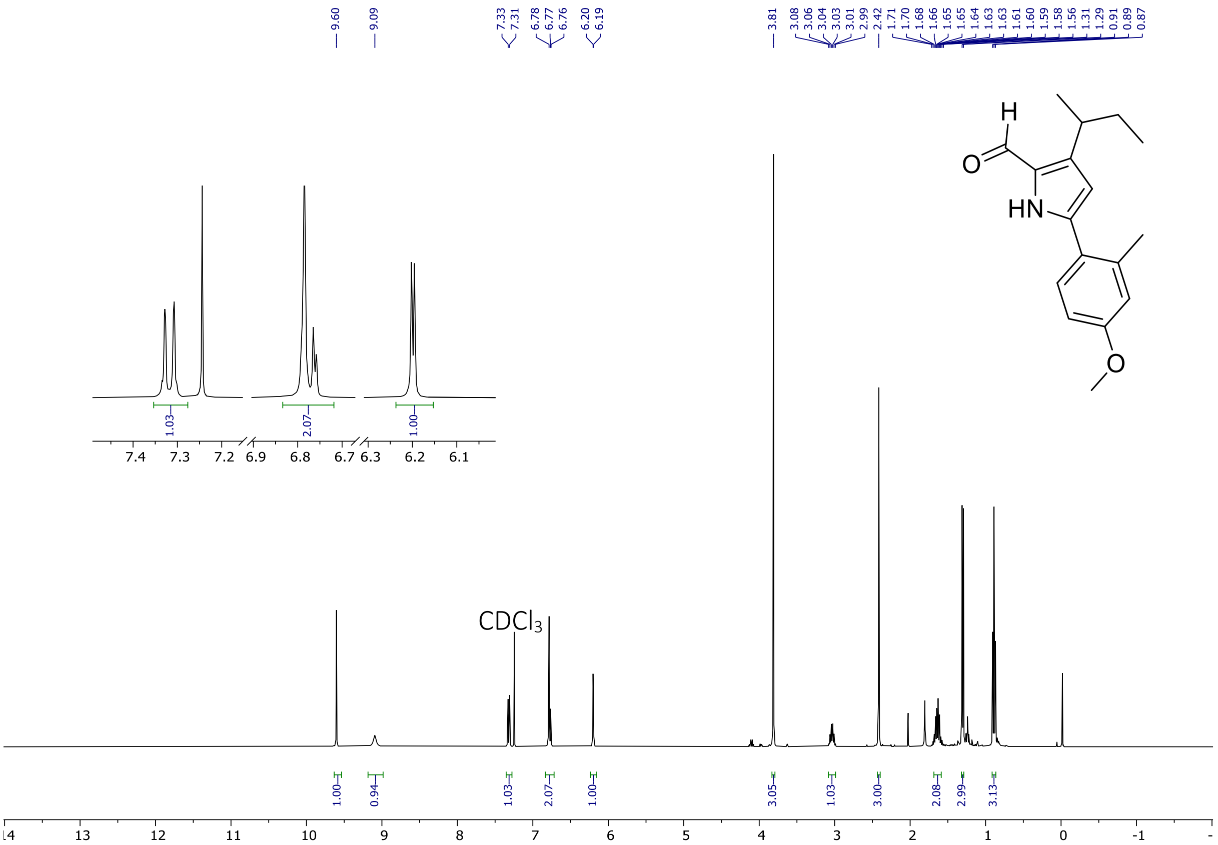

Figure S27. ^{1}H NMR spectrum (400 MHz, $CDCl_3$) of OMe-SCP-NH-αCOH.

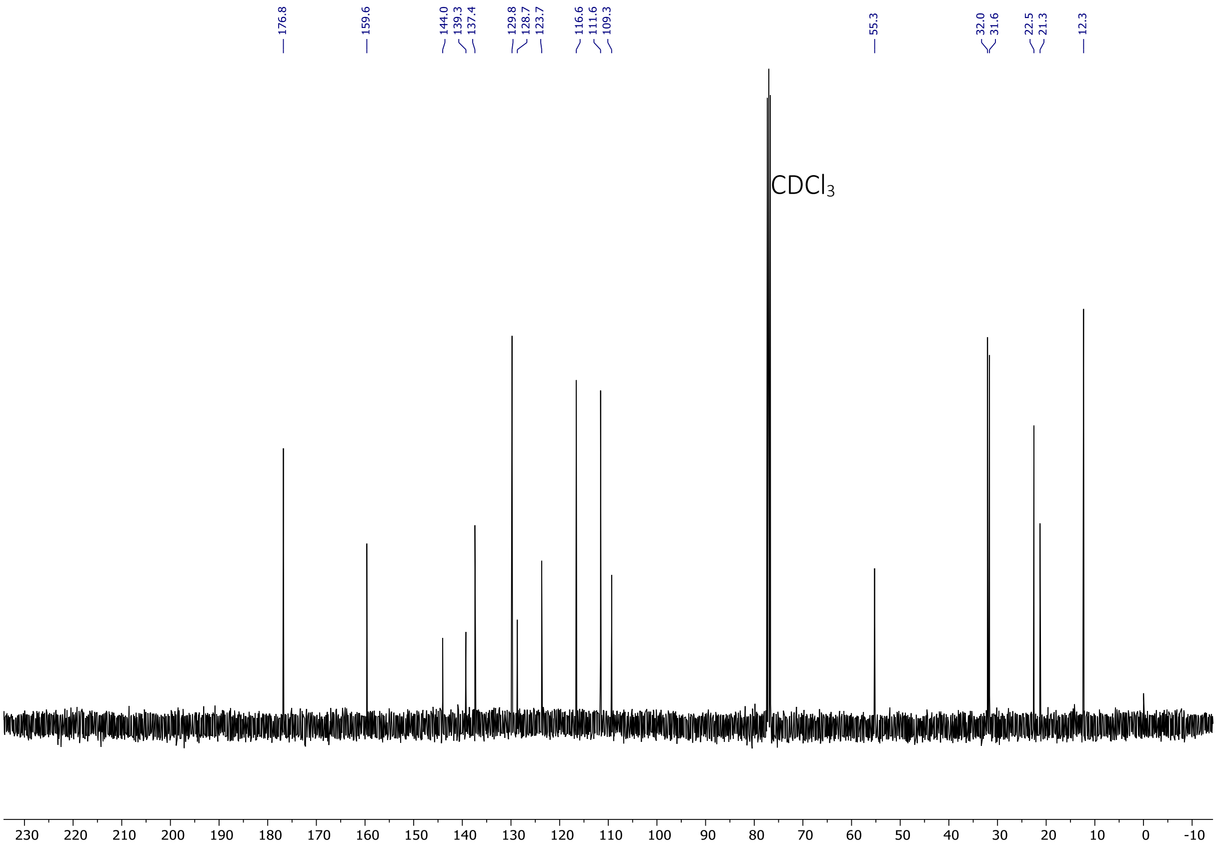

Figure S28. ^{13}C {^{1}H} NMR spectrum (101 MHz, $CDCl_3$) of OMe-SCP-NH-αCOH.

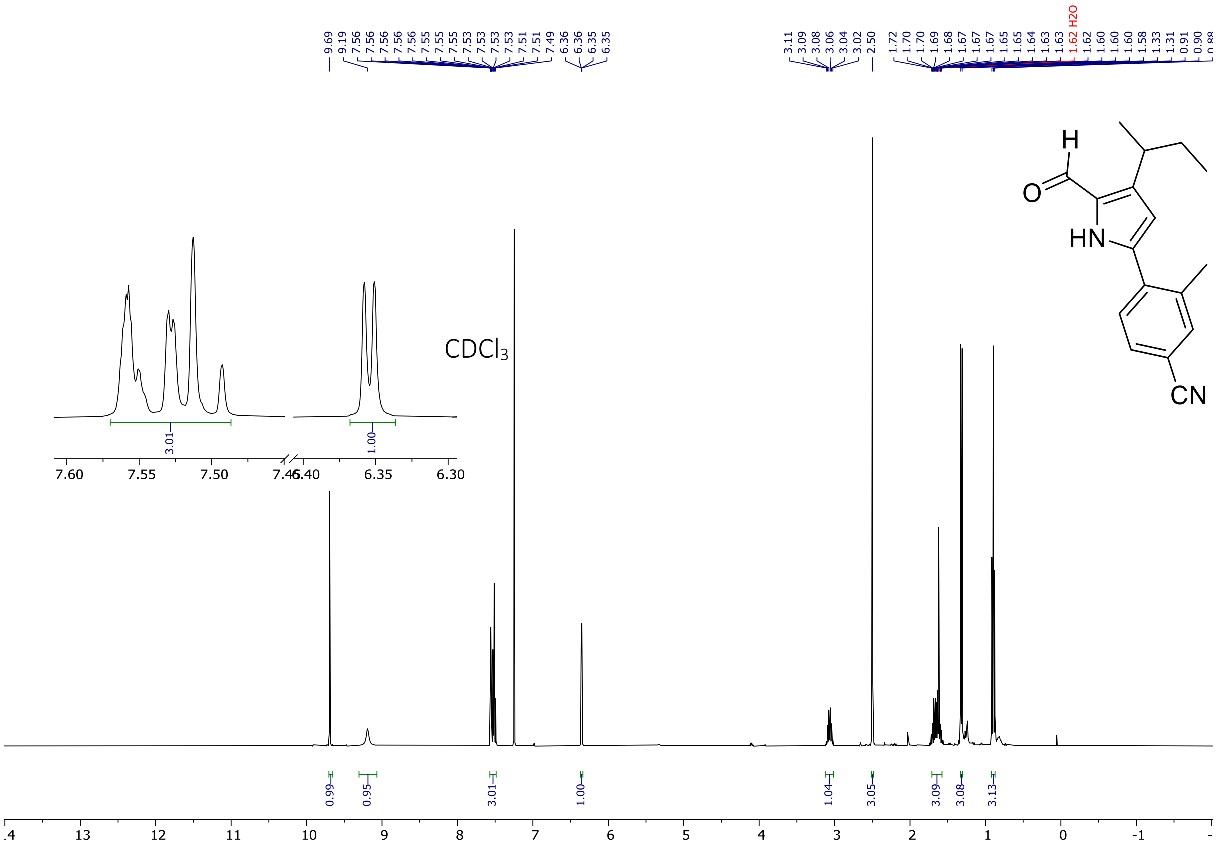

Figure S29. ^{1}H NMR spectrum (400 MHz, $CDCl_3$) of CN-SCP-NH-αCOH.

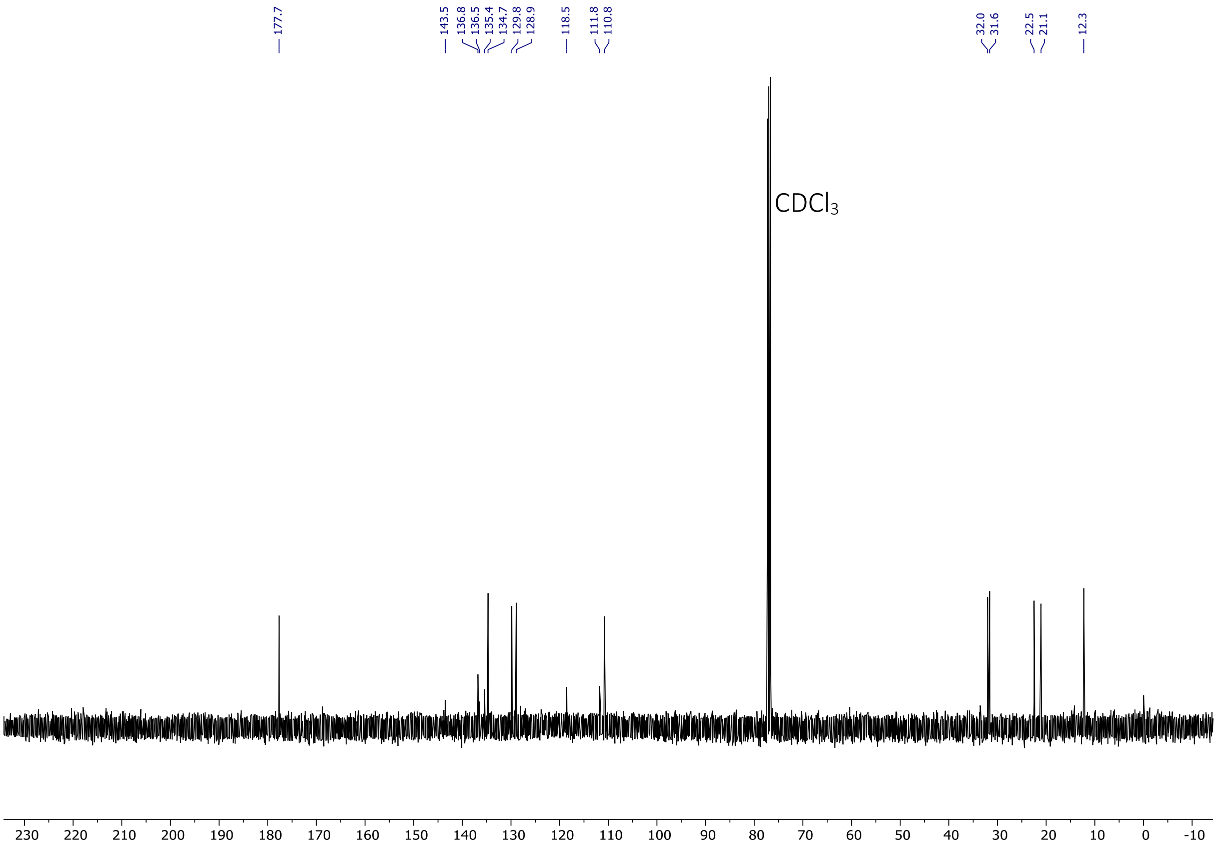

Figure S30. $^{13}C\{^1H\}$ NMR spectrum (101 MHz, $CDCl_3$) of CN-SCP-NH-αCOH.

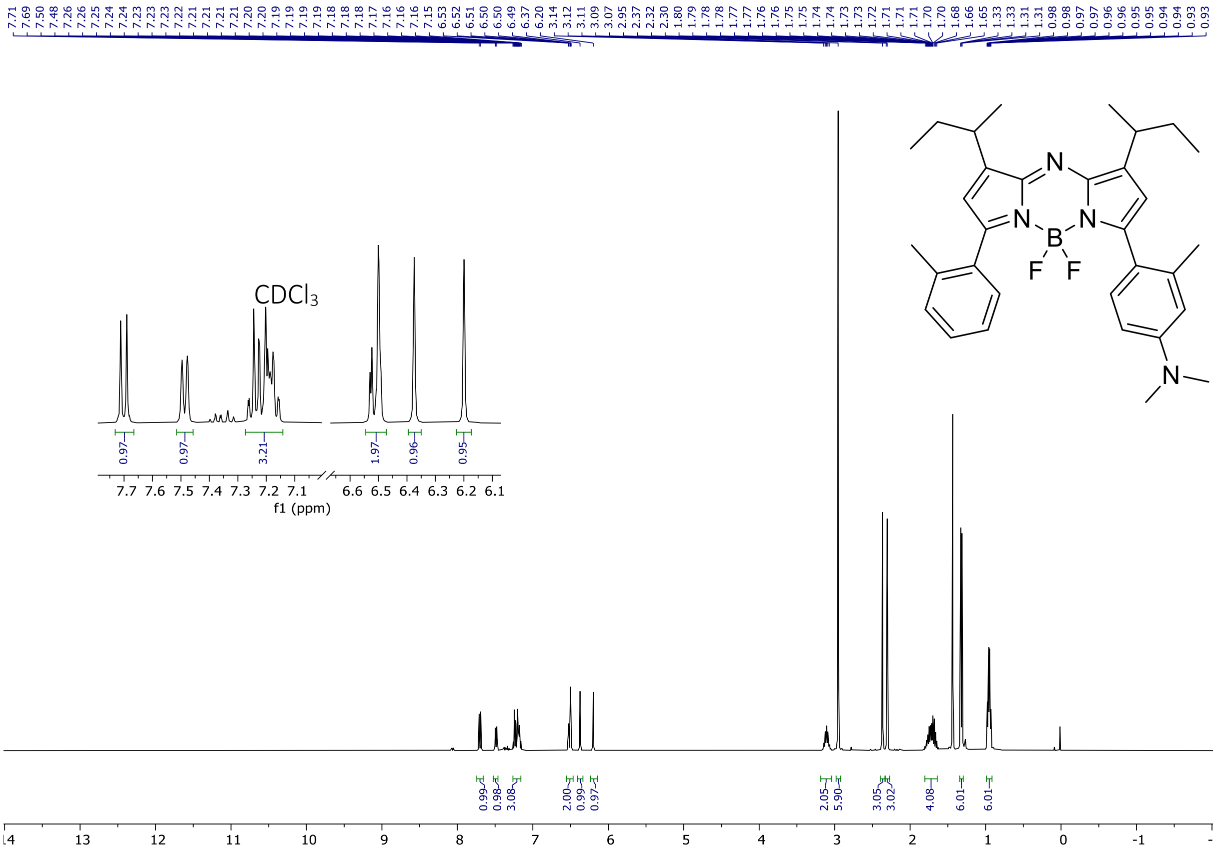

Figure S31. ^1H NMR spectrum (400 MHz, $CDCl_3$) of Aza-H-NMe_2.

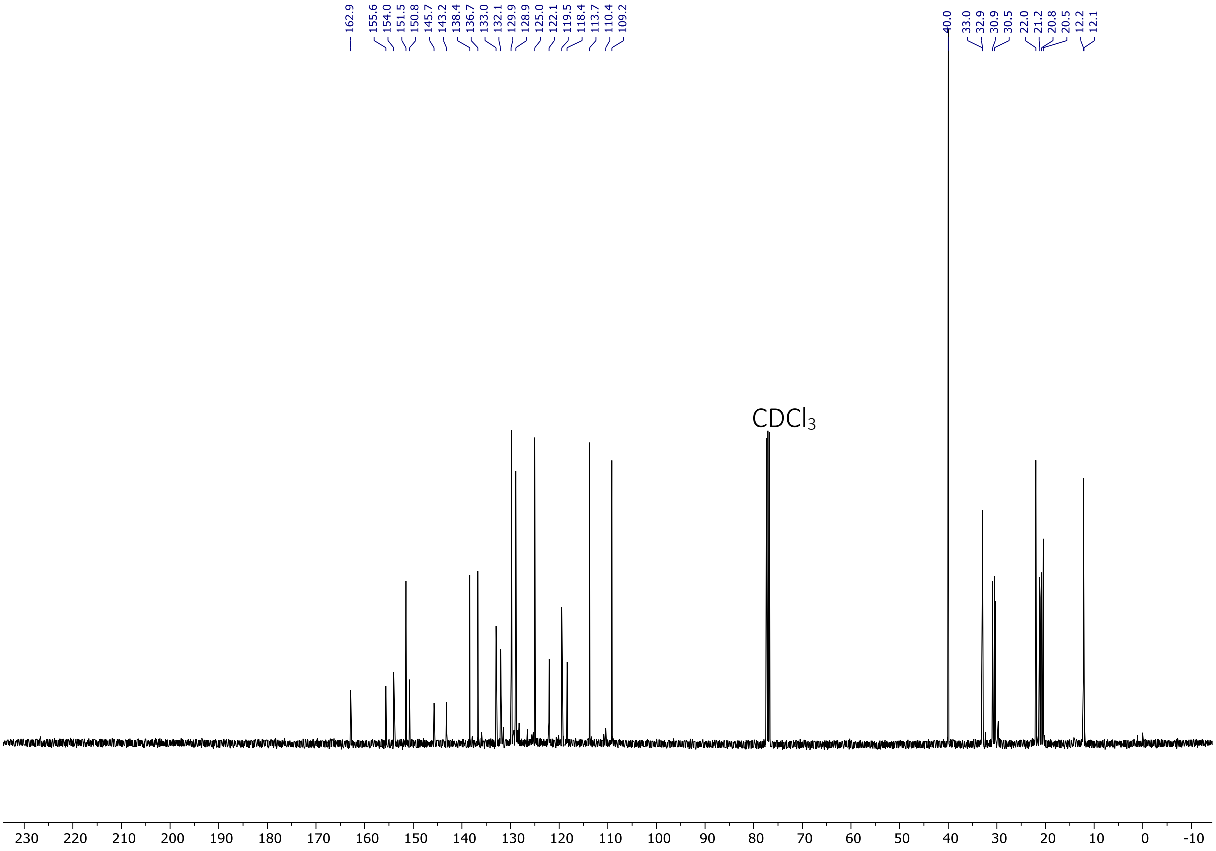

Figure S32. ^{13}C {^{1}H} NMR spectrum (101 MHz, $CDCl_3$) of Aza-H-NMe_2.

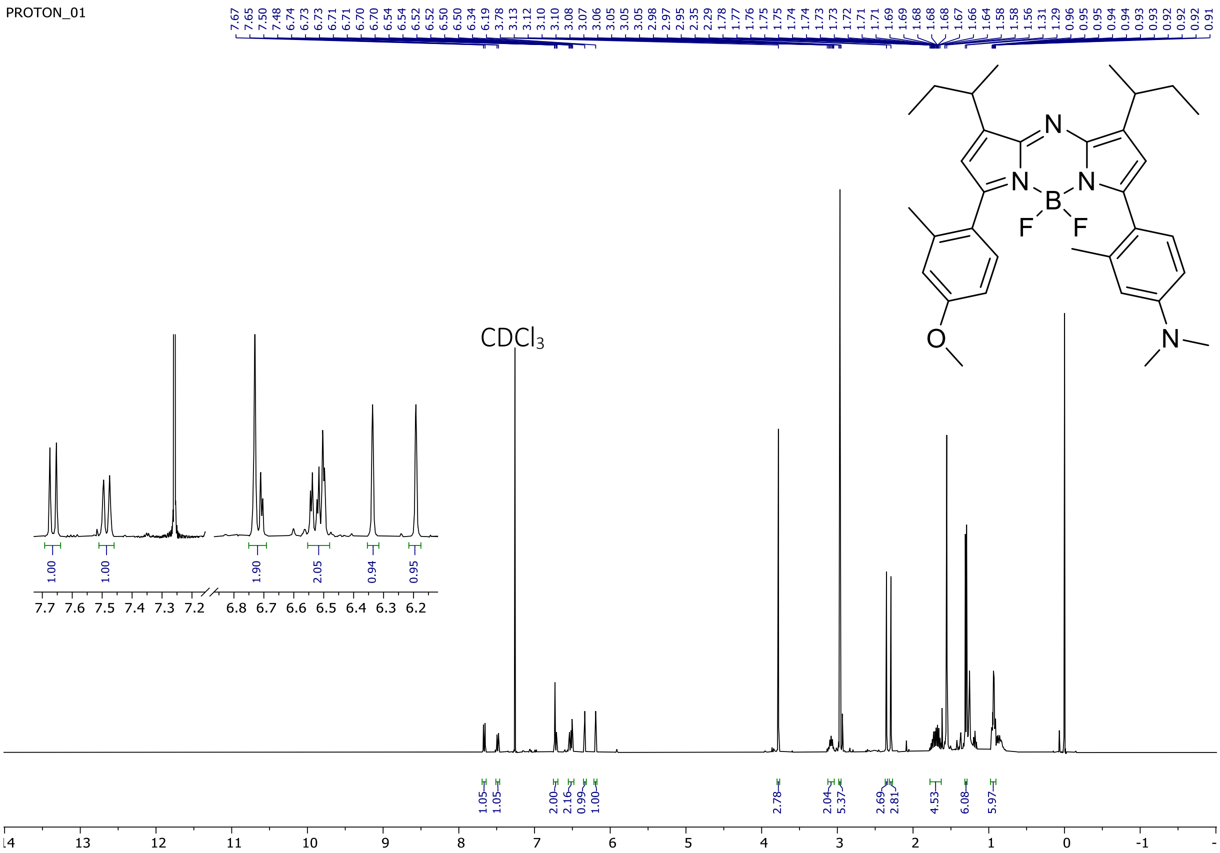

Figure S33. ^{1}H NMR spectrum (400 MHz, $CDCl_3$) of Aza-OMe-NMe_2.

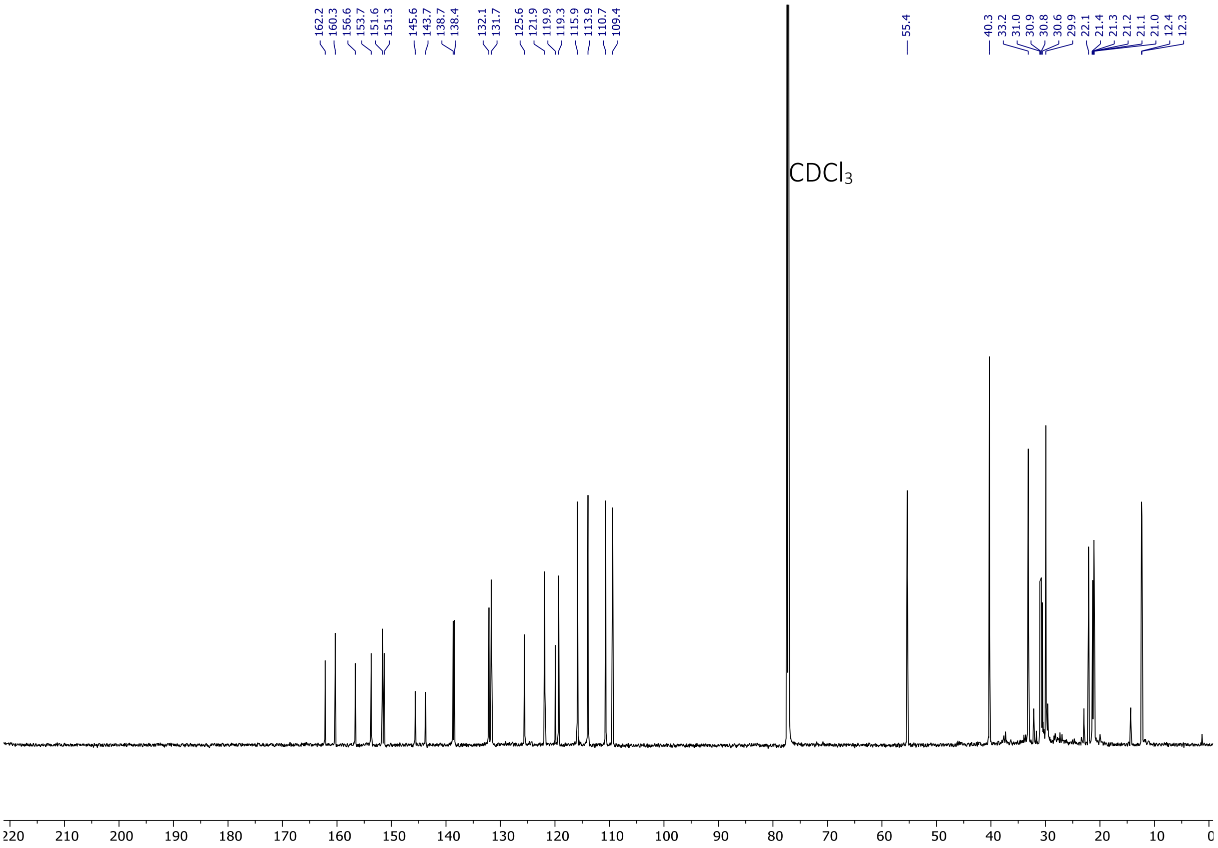

Figure S34. ^{13}C {^{1}H} NMR spectrum (201 MHz, $CDCl_3$) of Aza-OMe-NMe₂.

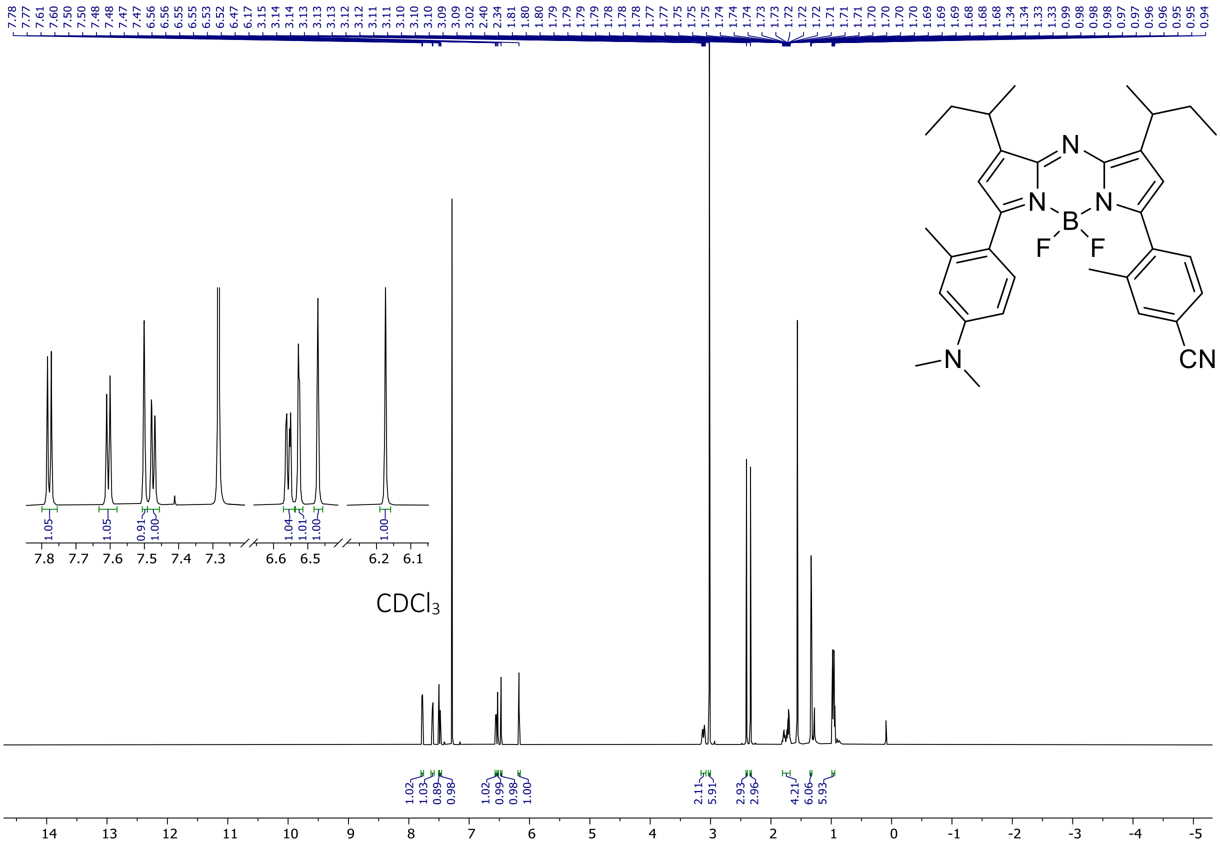

Figure S35. ^{1}H NMR spectrum (800 MHz, $CDCl_3$) of Aza-CN-NMe₂.

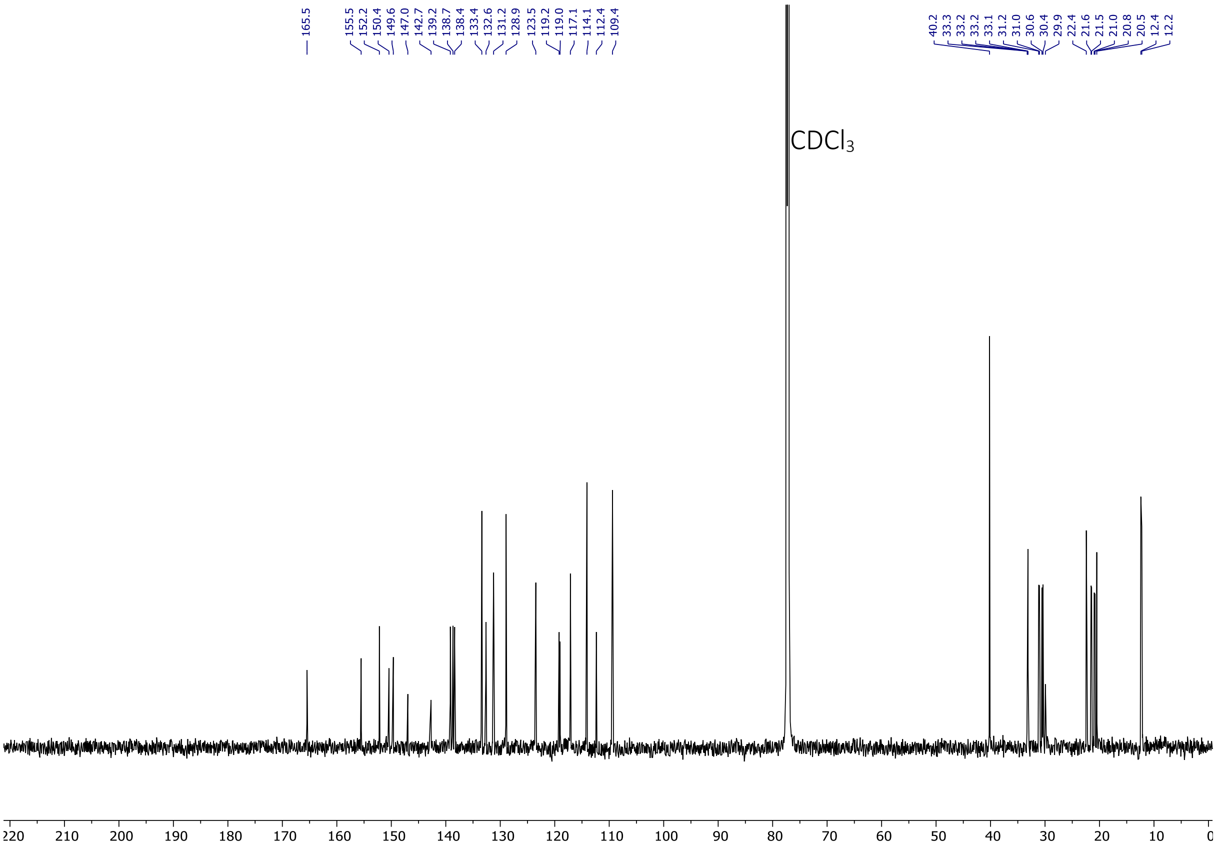

Figure S36. $^{13}C\{^{1}H\}$ NMR spectrum (201 MHz, $CDCl_3$) of Aza-CN-NMe$_2$.

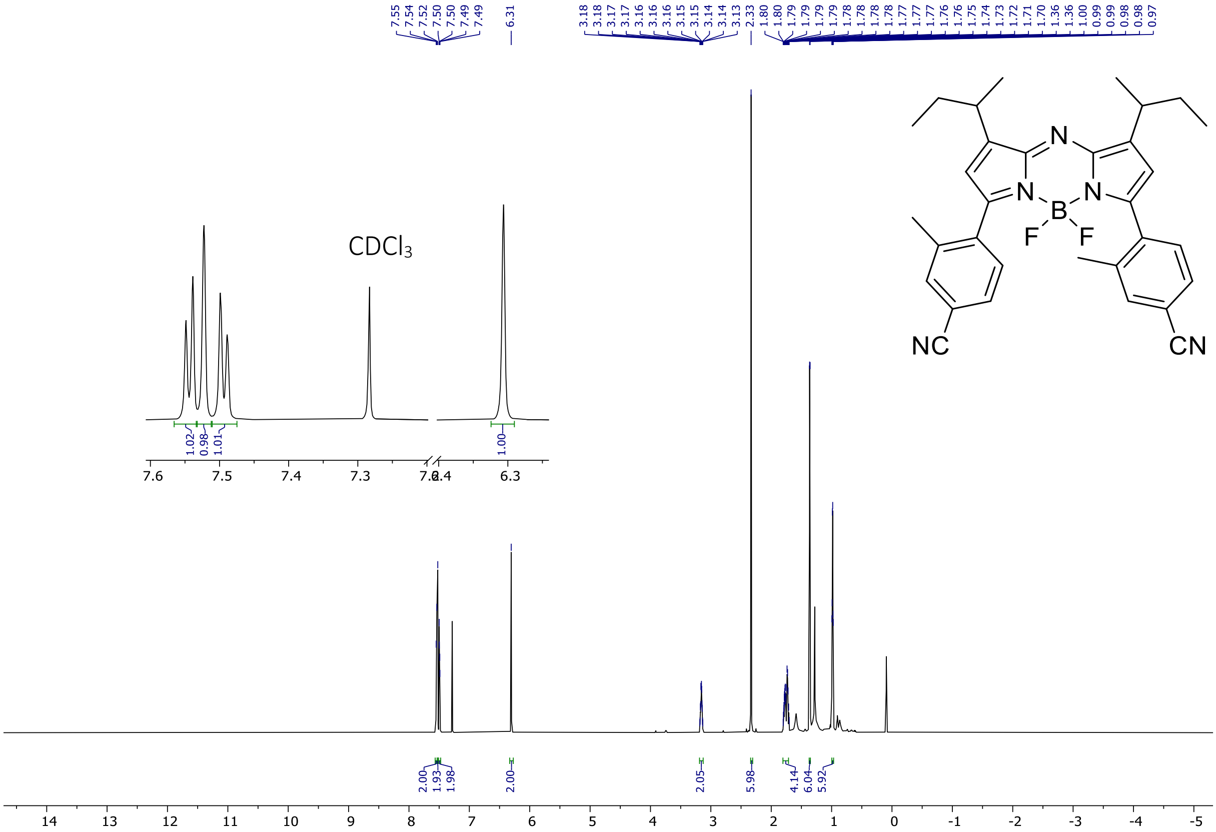

Figure S37. ^{1}H NMR spectrum (800 MHz, $CDCl_3$) of Aza-CN-CN.

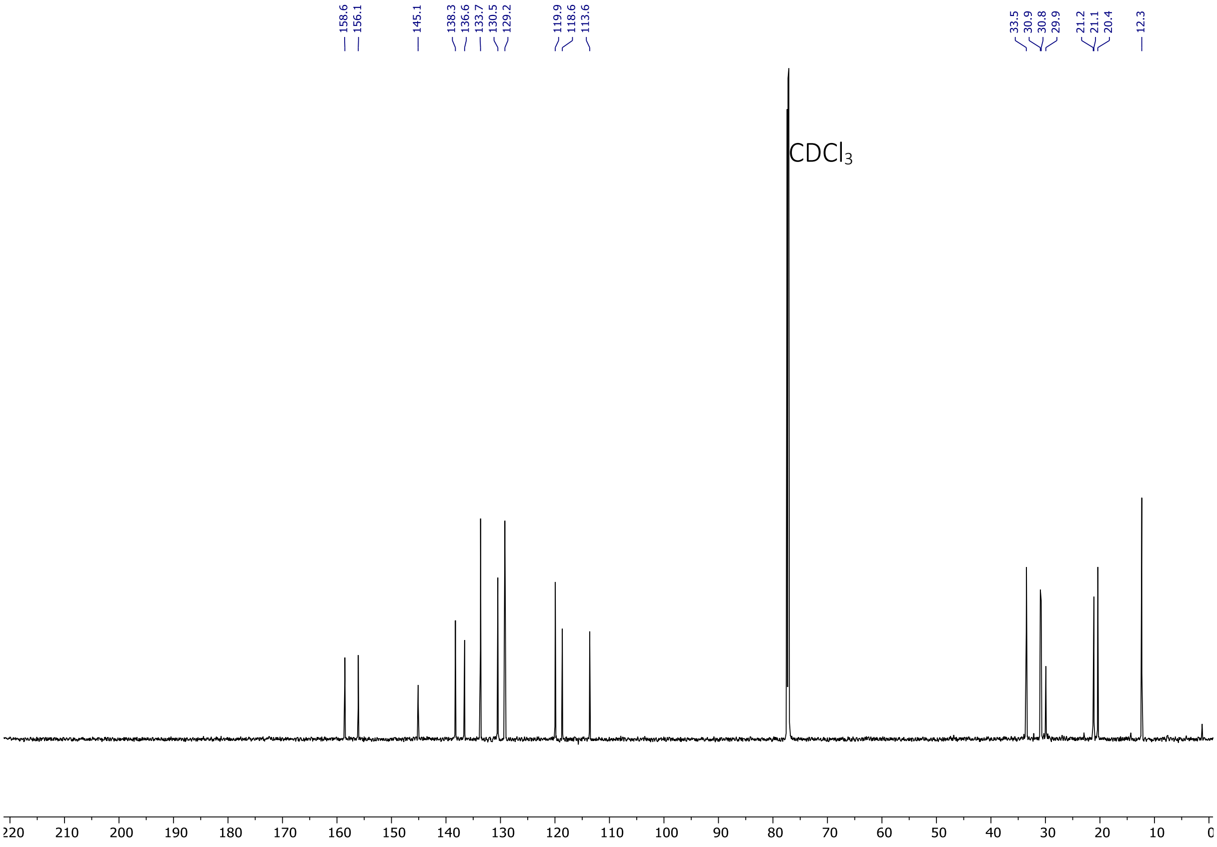

Figure S38. ^{13}C {^{1}H} NMR spectrum (201 MHz, $CDCl_3$) of Aza-CN-CN.

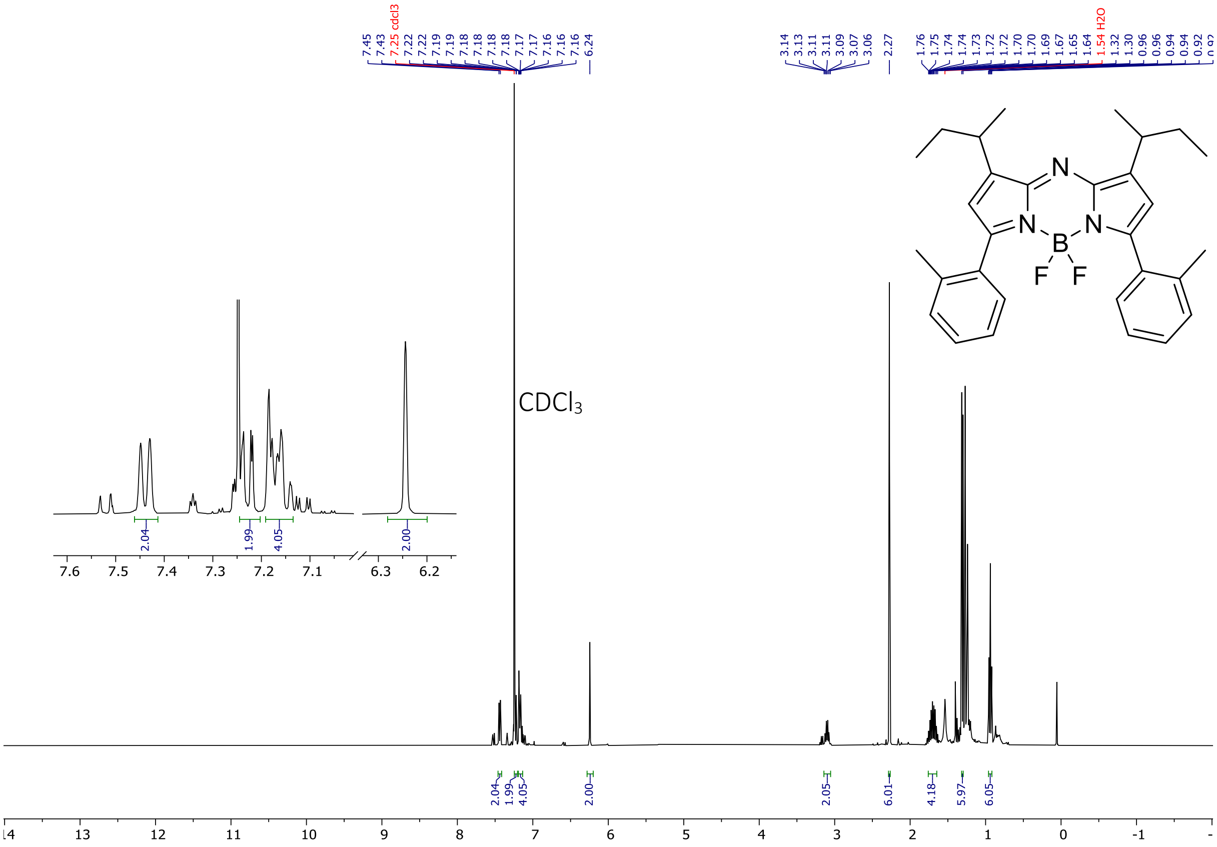

Figure S39. ^{1}H NMR spectrum (400 MHz, $CDCl_3$) of Aza-H-H.

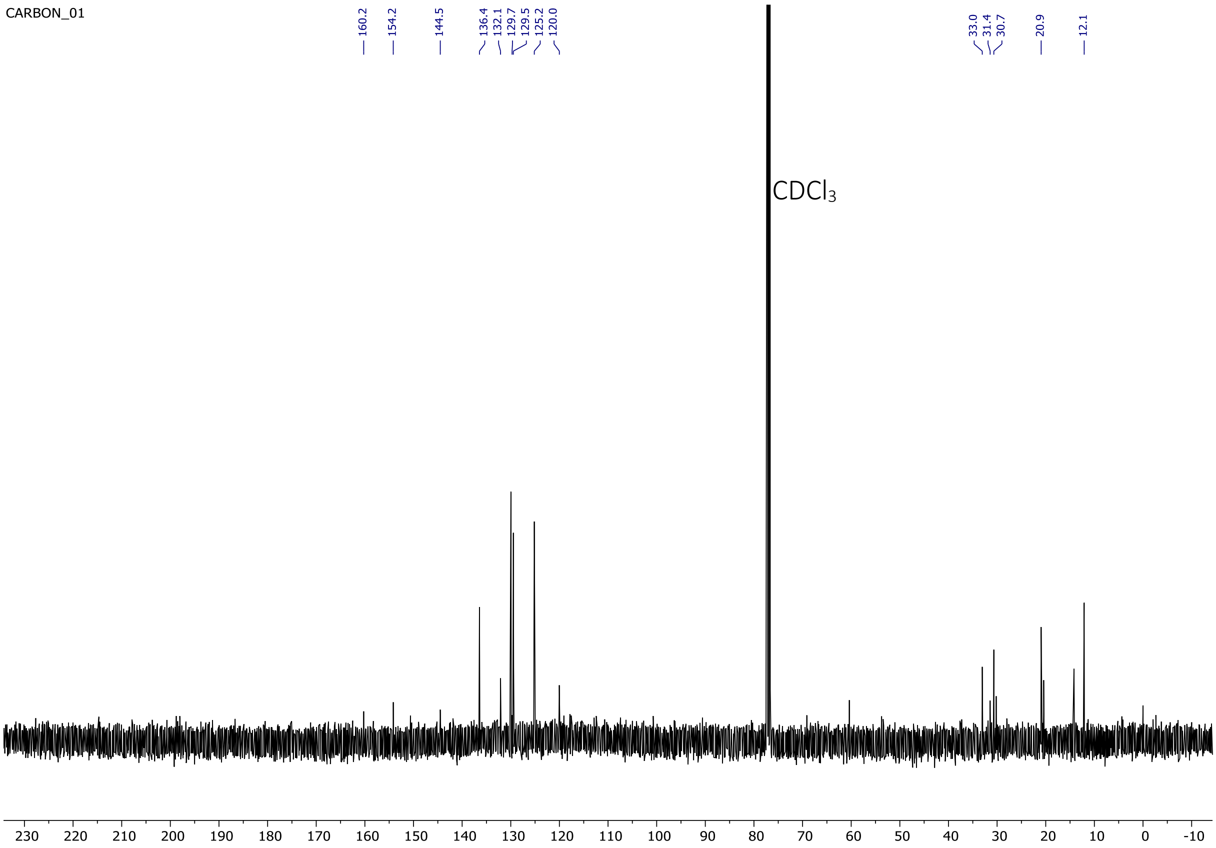

Figure S40. ^{13}C {^{1}H} NMR spectrum (101 MHz, $CDCl_3$) of Aza-H-H.

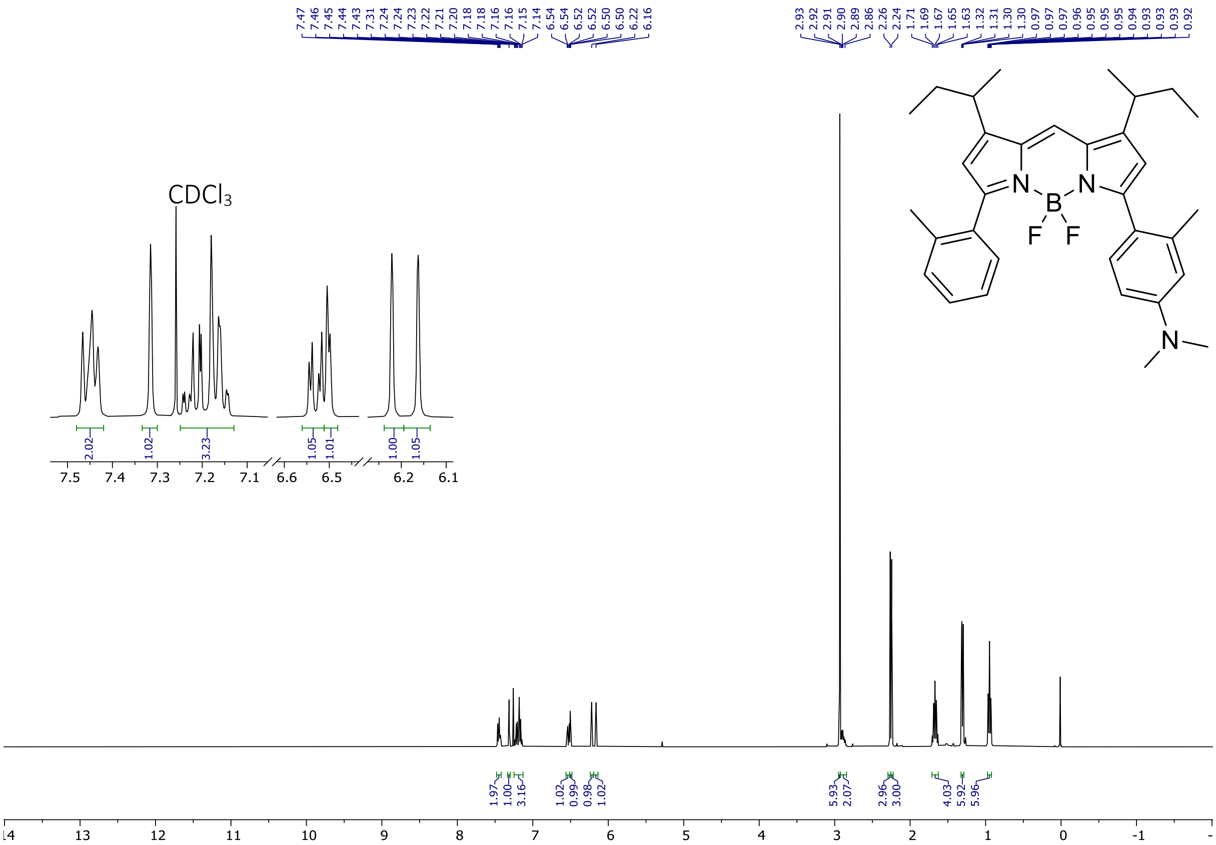

Figure S41. ^{1}H NMR spectrum (400 MHz, $CDCl_3$) of H-NMe$_2$-BODIPY.

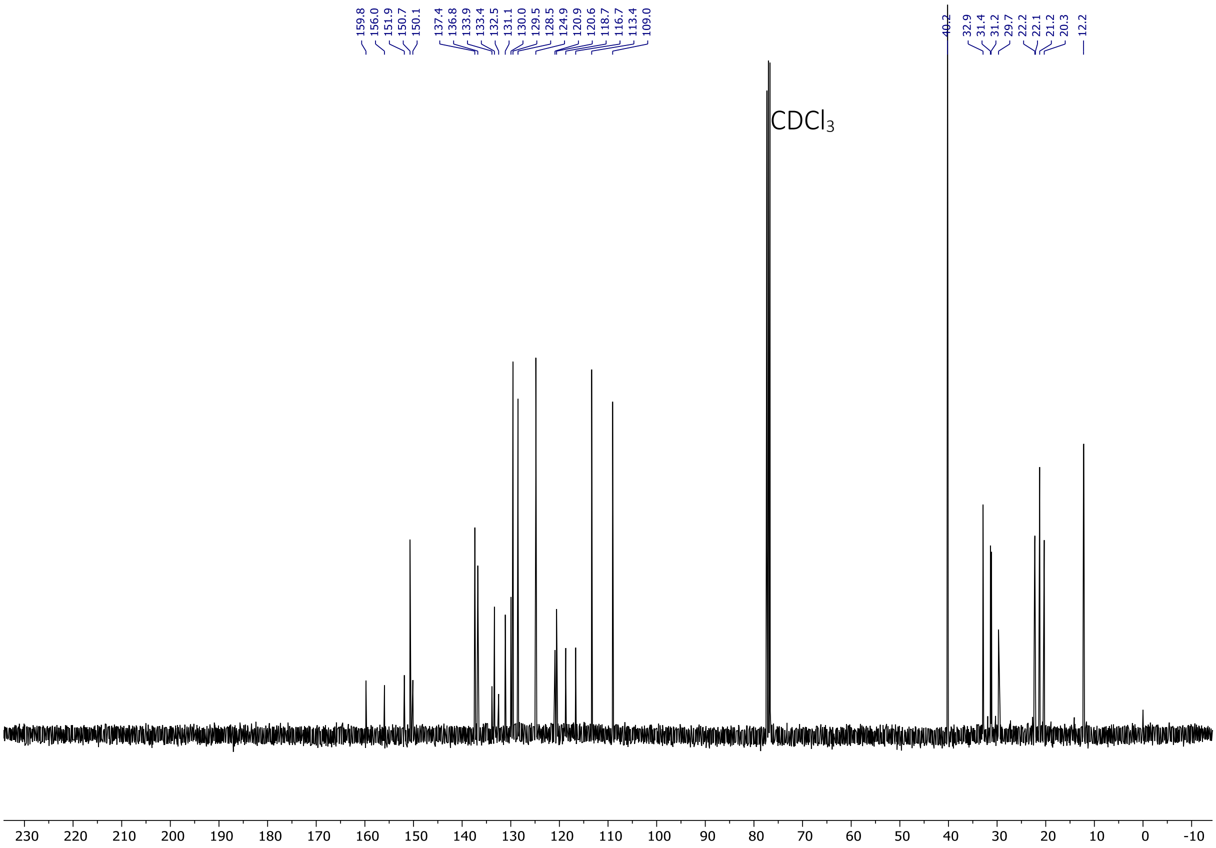

Figure S42. $^{13}C\ \{^{1}H\}$ NMR spectrum (101 MHz, $CDCl_3$) of H-NMe$_2$-BODIPY.

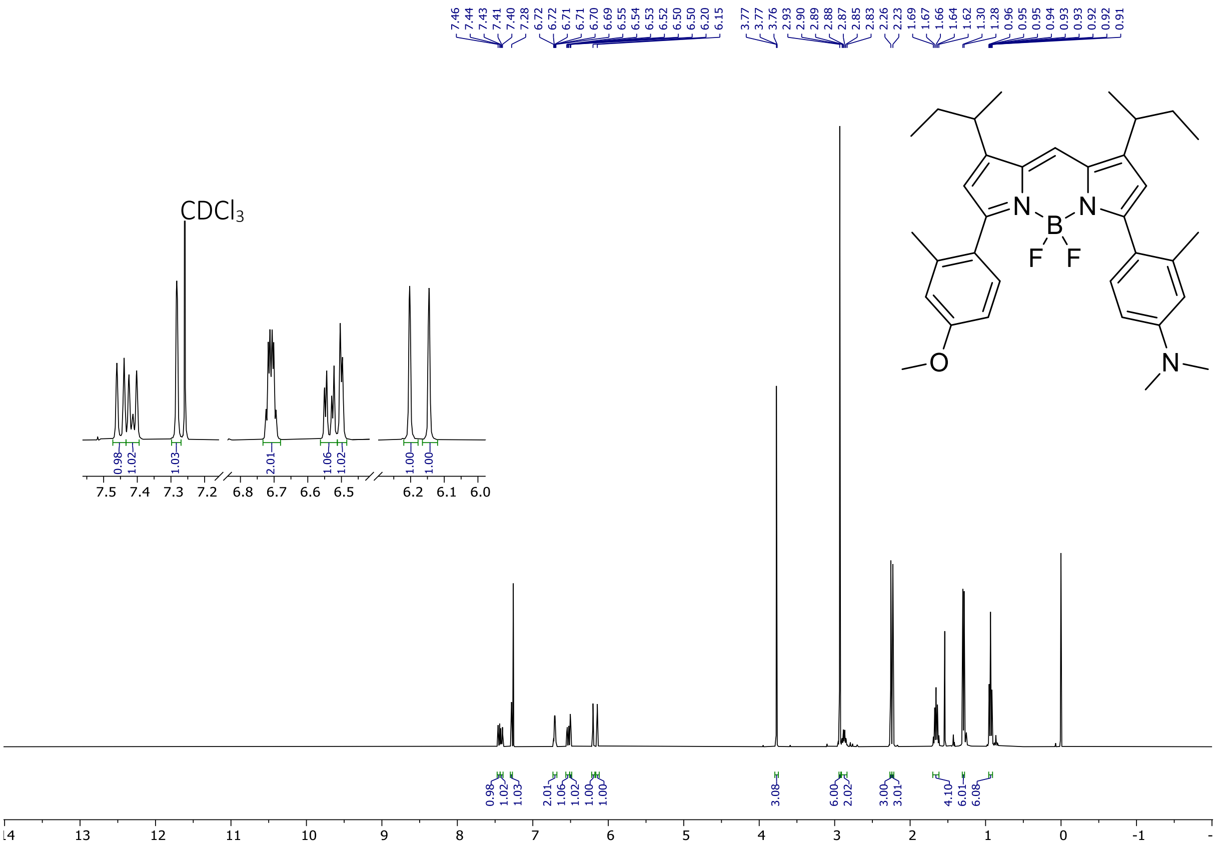

Figure S43. ^{1}H NMR spectrum (400 MHz, $CDCl_3$) of OMe-NMe$_2$-BODIPY.

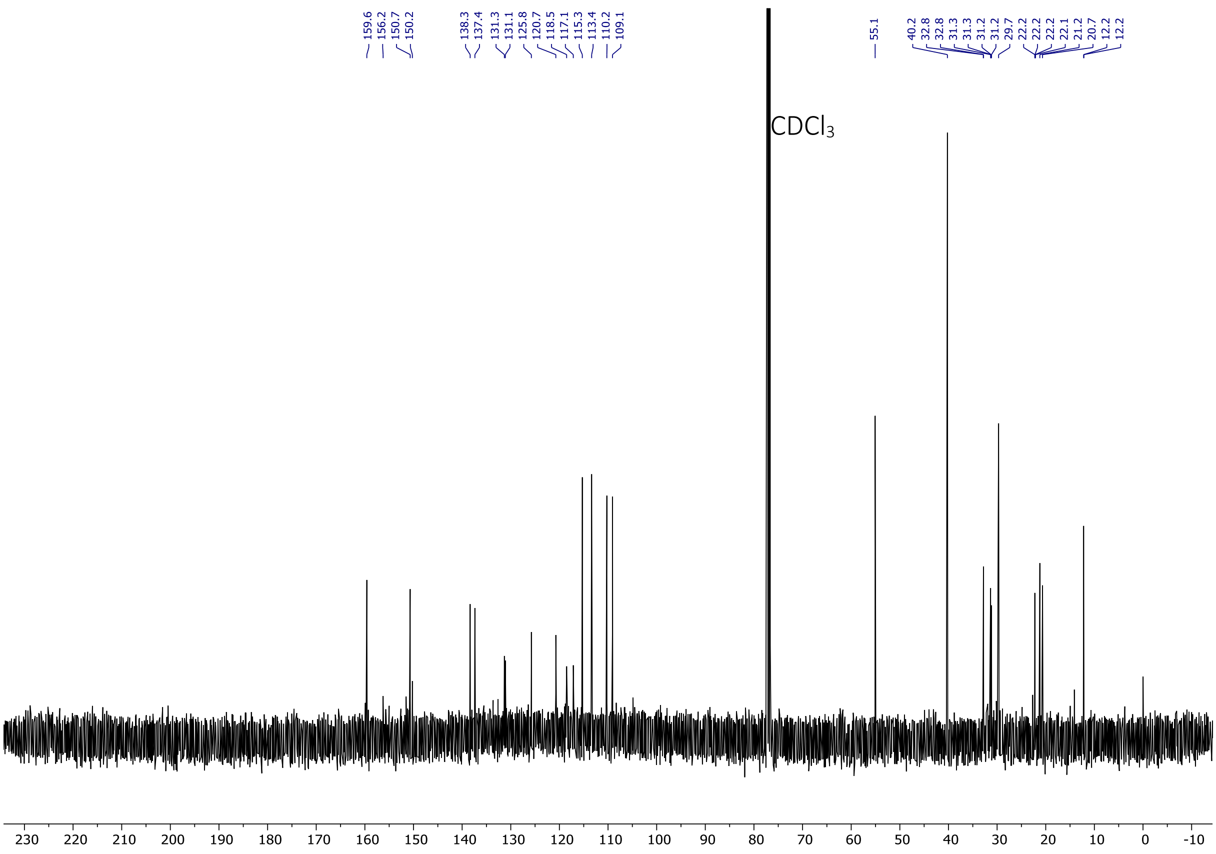

Figure S44. $^{13}C\{^{1}H\}$ NMR spectrum (101 MHz, $CDCl_3$) of OMe-NMe$_2$-BODIPY.

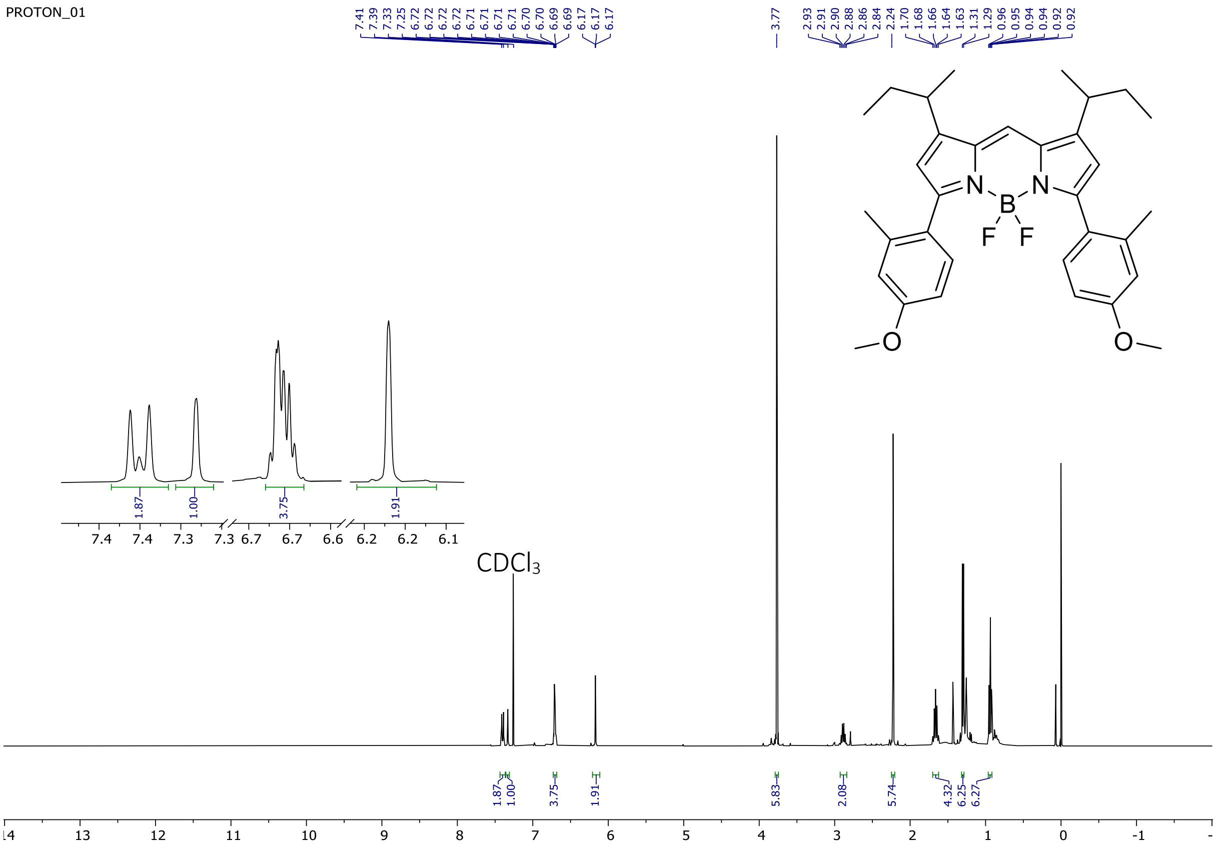

Figure S45. ^{1}H NMR spectrum (400 MHz, $CDCl_3$) of OMe-OMe-BODIPY.

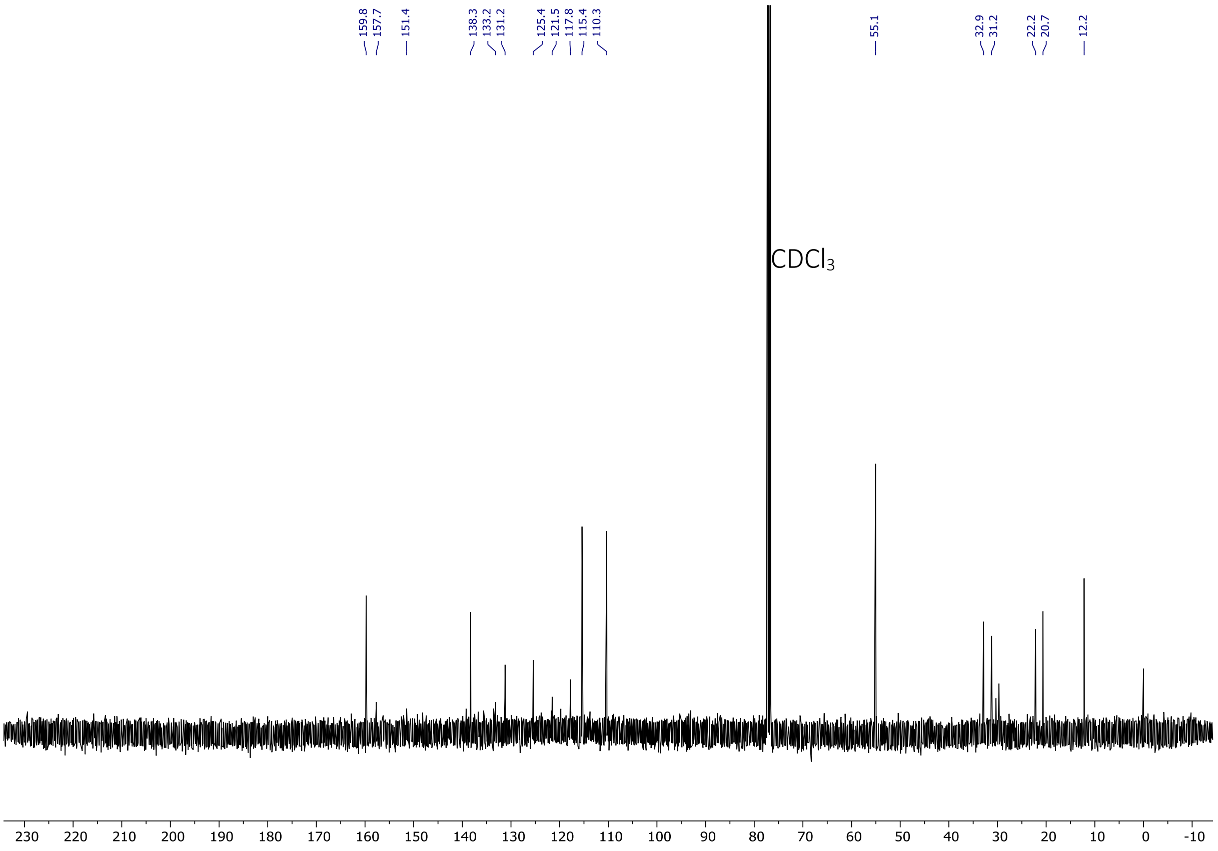

Figure S46. ^{13}C {^{1}H} NMR spectrum (101 MHz, $CDCl_3$) of OMe-OMe-BODIPY.

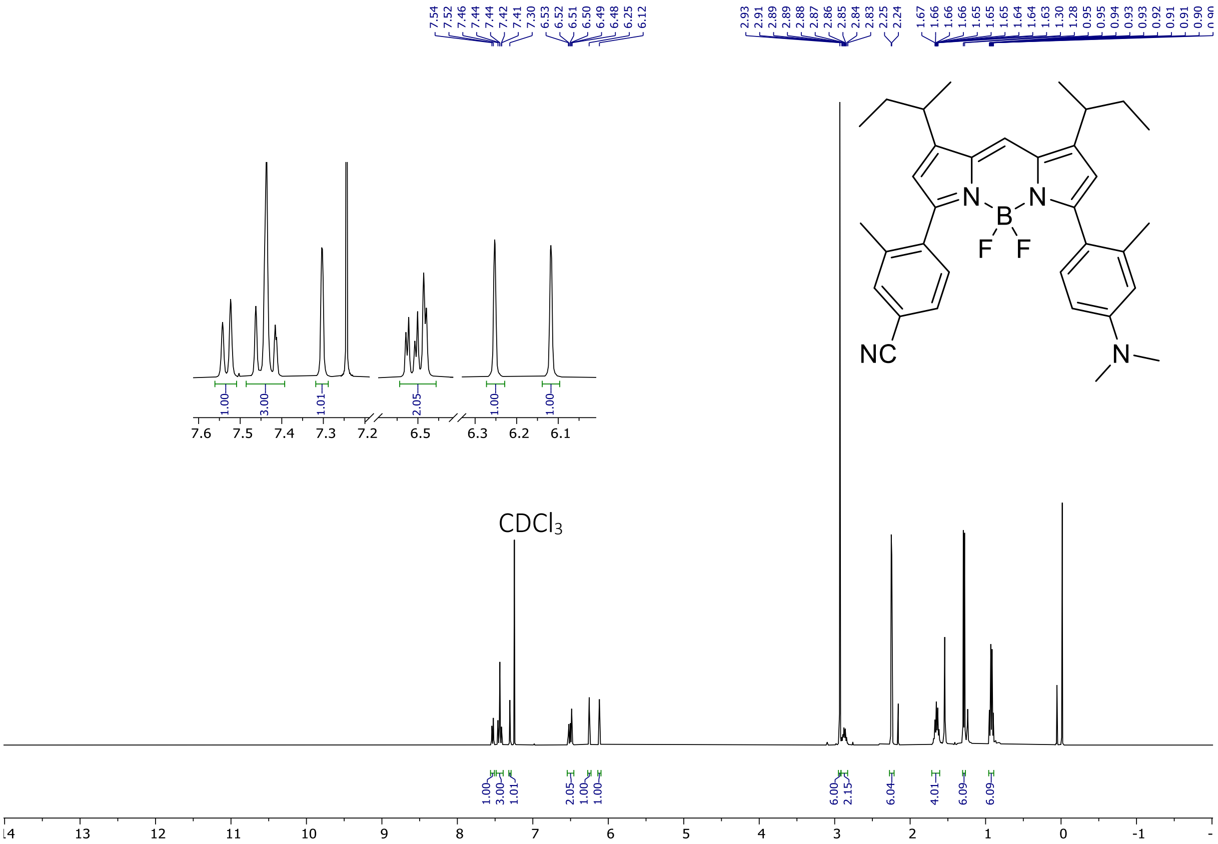

Figure S47. ^{1}H NMR spectrum (400 MHz, $CDCl_3$) of CN-NMe_2-BODIPY.

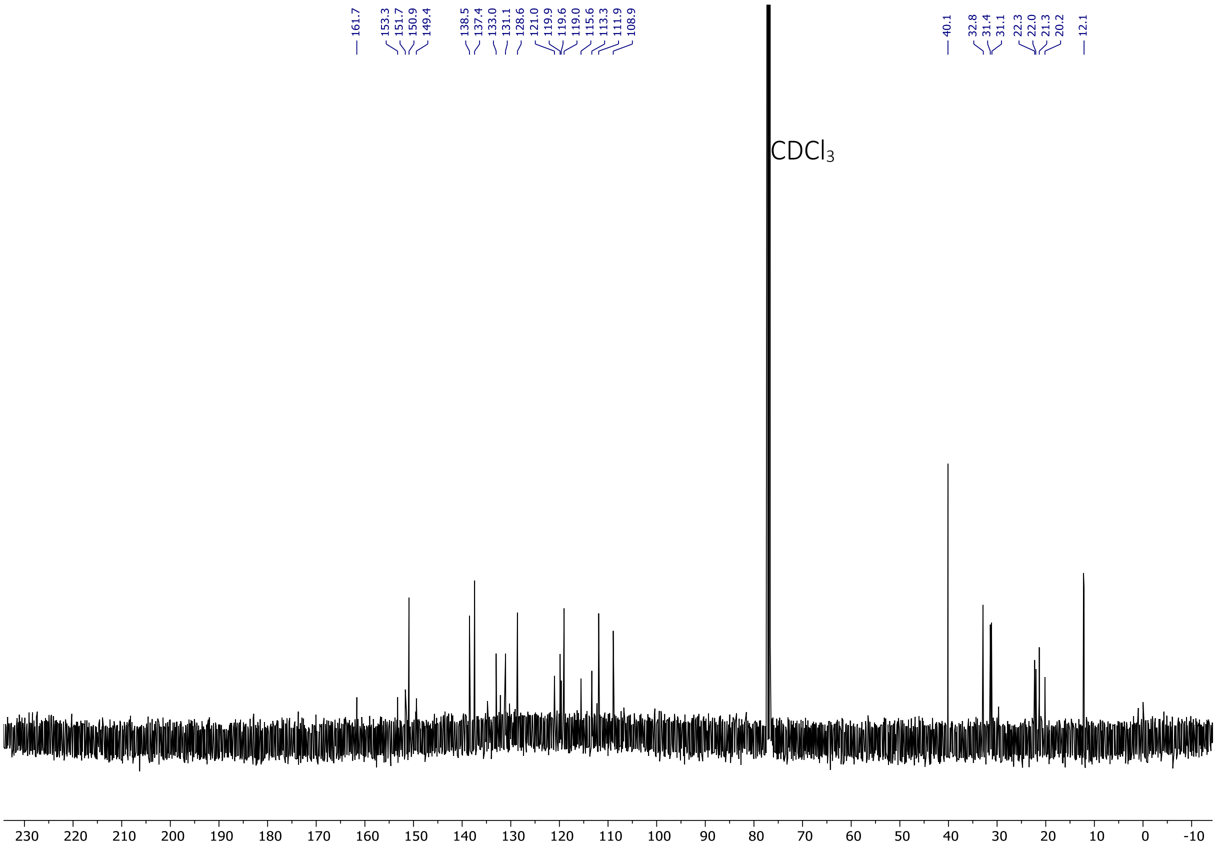

Figure S48. $^{13}C\ \{^{1}H\}$ NMR spectrum (101 MHz, $CDCl_3$) of CN-NMe$_2$-BODIPY.

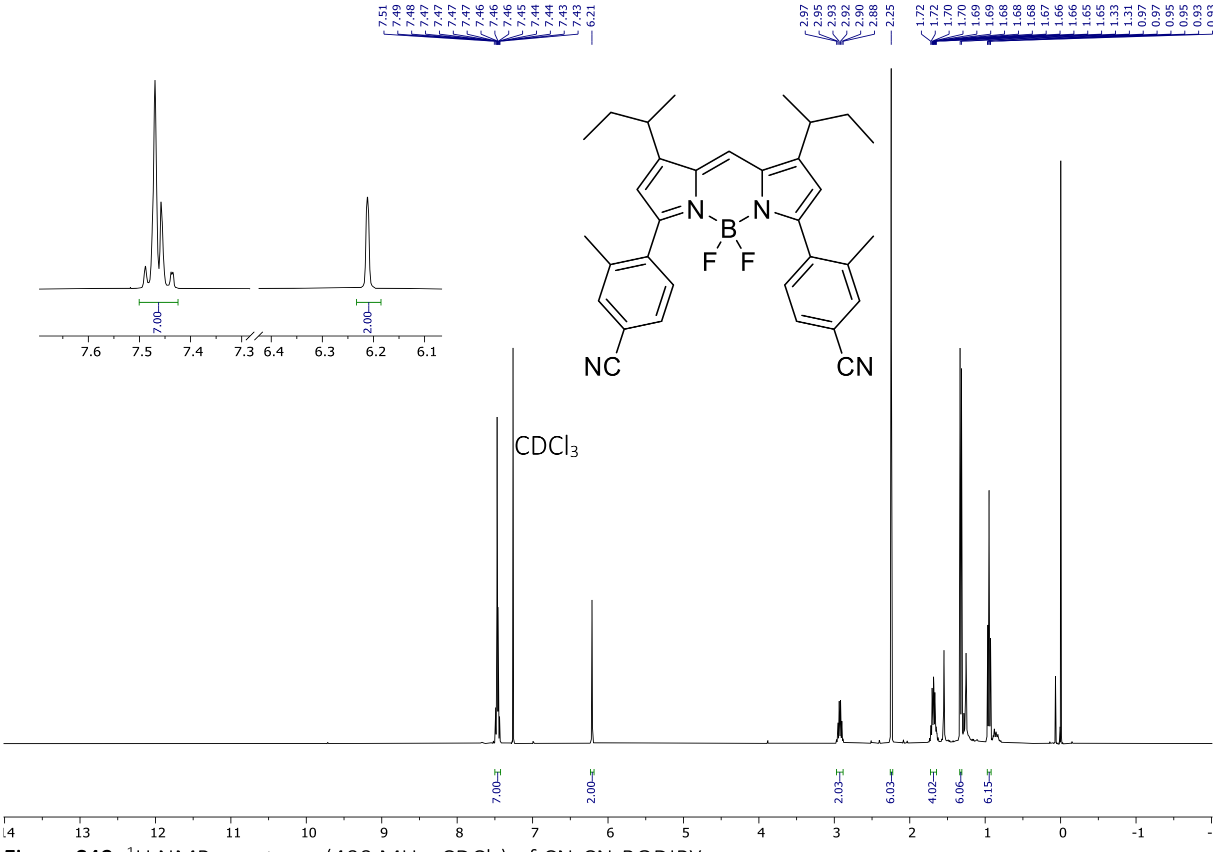

Figure S49. ^{1}H NMR spectrum (400 MHz, $CDCl_3$) of CN-CN-BODIPY.

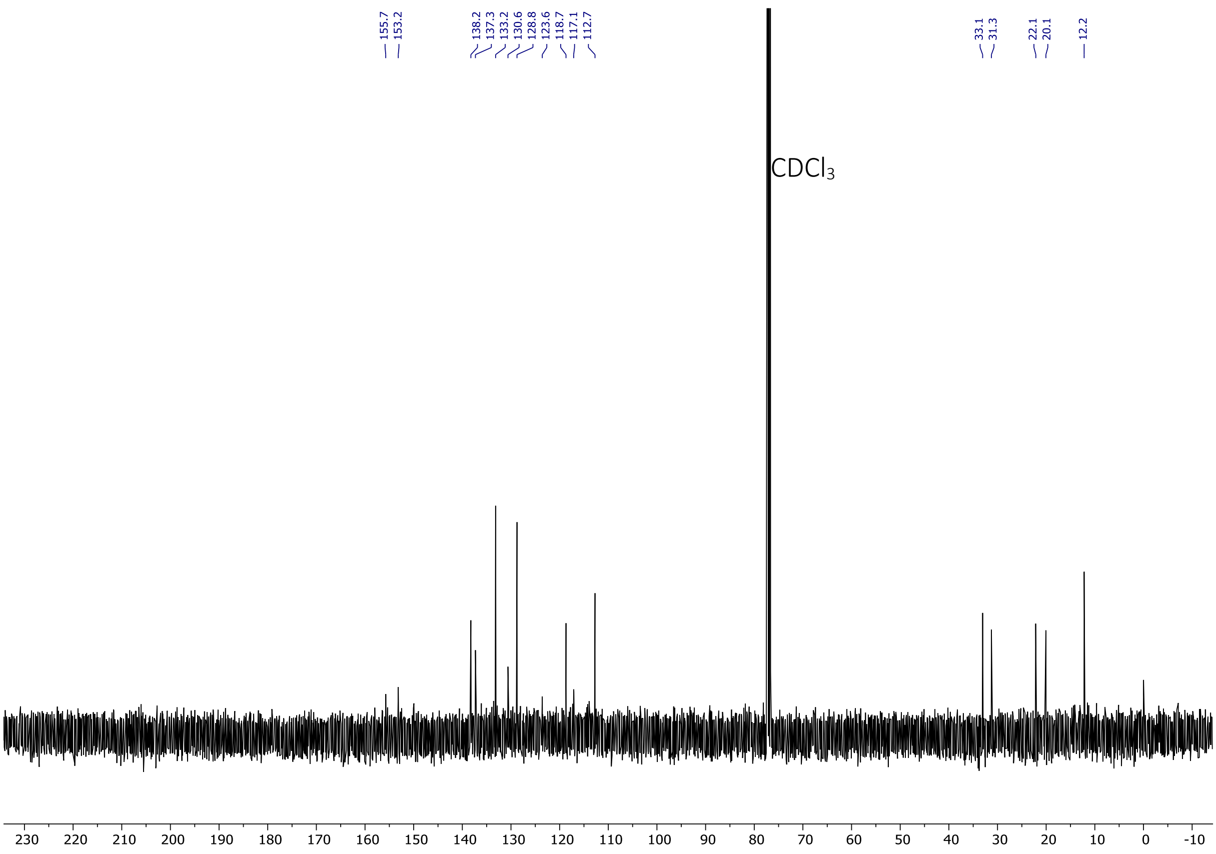

Figure S50. ^{13}C {^{1}H} NMR spectrum (101 MHz, $CDCl_3$) of CN-CN-BODIPY.

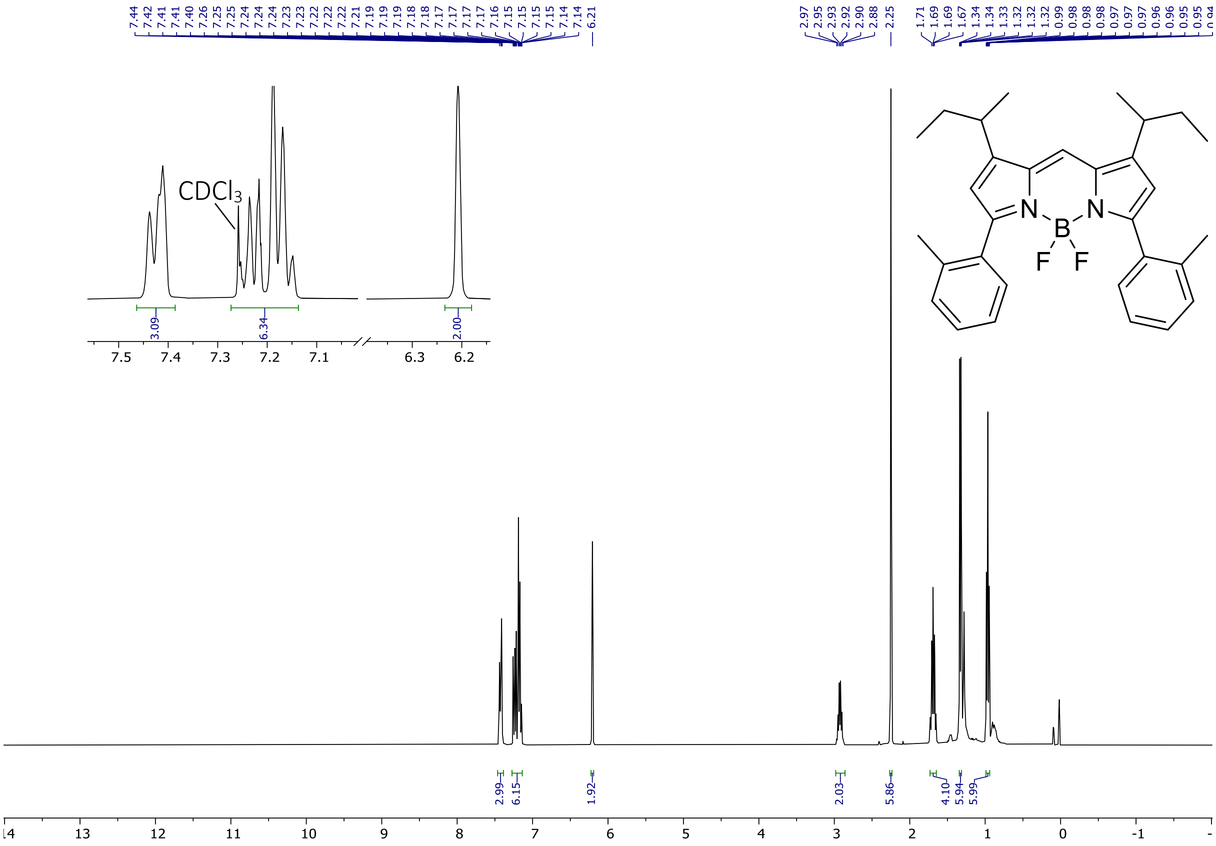

Figure S51. ^{1}H NMR spectrum (400 MHz, $CDCl_3$) of H-H-BODIPY.

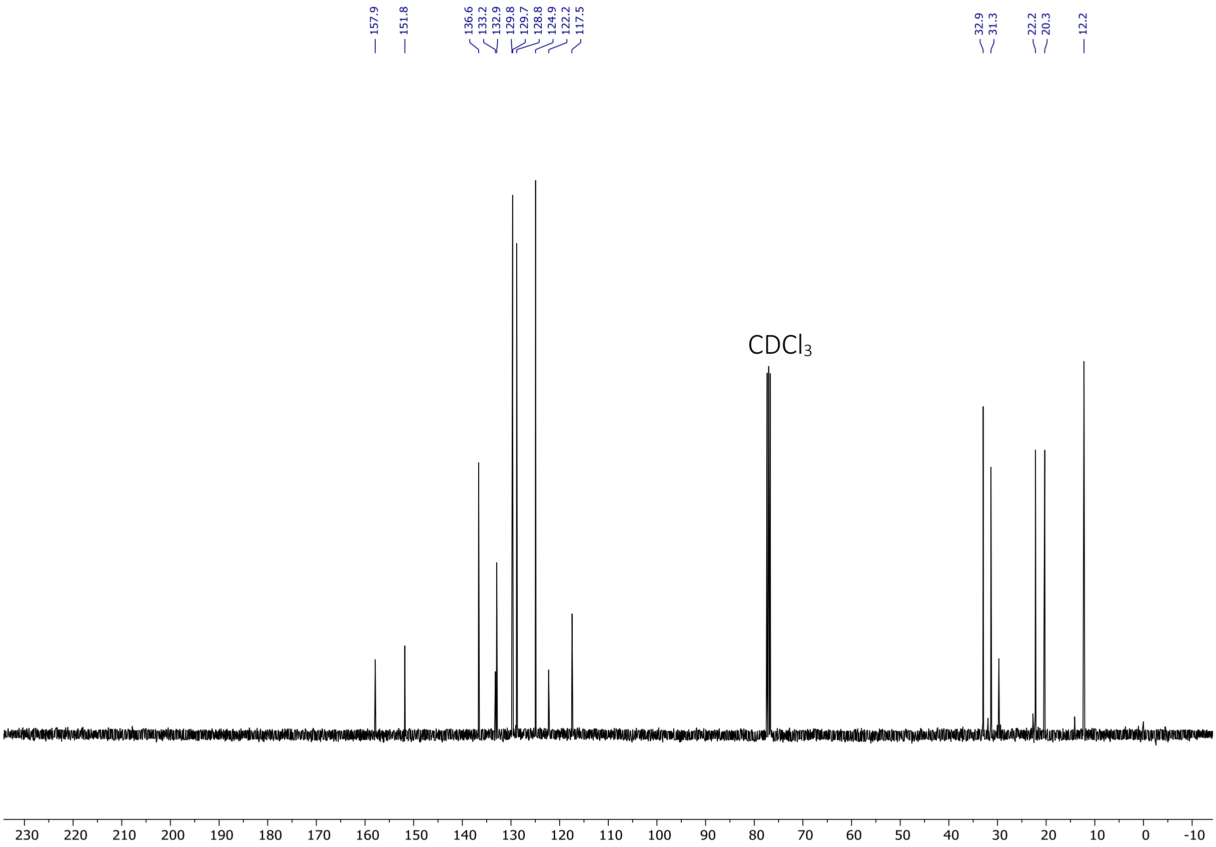

Figure S52. $^{13}C\ \{^1H\}$ NMR spectrum (101 MHz, $CDCl_3$) of H-H-BODIPY.
